# A novel algorithm for the virtual screening of extensive small molecule libraries against ERCC1/XPF protein-protein interaction for the identification of resistance-bypassing potential anticancer molecules

**DOI:** 10.55730/1300-0152.2686

**Published:** 2024-04-03

**Authors:** Salma GHAZY, Lalehan OKTAY, Serdar DURDAĞI

**Affiliations:** 1 Department of Biophysics, Computational Biology and Molecular Simulations Laboratory, School of Medicine, Bahçeşehir University, İstanbul, Turkiye; 2 Lab for Innovative Drugs (Lab4IND), Computational Drug Design Center (HITMER), Bahçeşehir University, İstanbul, Turkiye; 3 Department of Pharmaceutical Chemistry, Molecular Therapy Lab, School of Pharmacy, Bahçeşehir University, İstanbul, Turkiye

**Keywords:** Excision repair cross complementation group 1, xeroderma pigmentosum complementation group F, inhibitor, protein-protein interaction, cancer

## Abstract

**Background and aim:**

Cancer cell’s innate chemotherapeutic resistance continues to be an obstacle in molecular oncology. This theory is firmly tied to the cancer cells’ integral DNA repair mechanisms continuously neutralizing the effects of chemotherapy. Amidst these mechanisms, the nuclear excision repair pathway is crucial in renovating DNA lesions prompted by agents like Cisplatin. The ERCC1/XPF complex stands center-stage as a structure-specific endonuclease in this repair pathway. Targeting the ERCC1/XPF dimerization brings forth a strategy to augment chemotherapy by eschewing the resistance mechanism integral to cancer cells. This study tracks and identifies small anticancer molecules, with ERCC1/XPF inhibiting potential, within extensive small-molecule compound libraries.

**Materials and methods:**

A novel hybrid virtual screening algorithm, conjoining ligand- and target-based approaches, was developed. All-atom molecular dynamics (MD) simulations were then run on the obtained hit molecules to reveal their structural and dynamic contributions within the binding site. MD simulations were followed by MM/GBSA calculations to qualify the change in binding free energies of the protein/ligand complexes throughout MD simulations.

**Results:**

Conducted analyses highlight new potential inhibitors AN-487/40936989 from the SPECS SC library, K219-1359, and K786-1161 from the ChemDiv Representative Set library as showing better predicted activity than previously discovered ERCC1/XPF inhibitor, CHEMBL3617209.

**Conclusion:**

The algorithm implemented in this study expands our comprehension of chemotherapeutic resistance and how to overcome it through identifying ERCC1/XPF inhibitors with the aim of enhancing chemotherapeutic impact giving hope for ameliorated cancer treatment outcomes.

## 1. Introduction

Cancer, as declared by the World Health Organization (WHO), remains one of the leading causes of death worldwide. According to GLOBOCAN, the cancer incidences are expected to increase to 28.4 million cases in 2040 – a 47% elevation from 2020. Research in the field of cancer treatment aims to enhance the effectiveness of cancer opposing agents while minimizing their adverse effects (Sung et al., 2021).

Cisplatin, also known as cis-diammine-dichloroplatinum II, stands as one of the most used chemotherapy drugs in cancer treatment. Its mechanism involves the formation of platinum-DNA adducts, particularly intrastrand crosslinks, through binding to the N7 position of purine resulting in a modified DNA activity inducing apoptosis. Nevertheless, numerous tumors seem to develop resistance to platinum-based chemotherapy by triggering DNA repair mechanisms capable of addressing such detrimental damage. (Kirschner and Melton, 2010).

**Figure 1:**
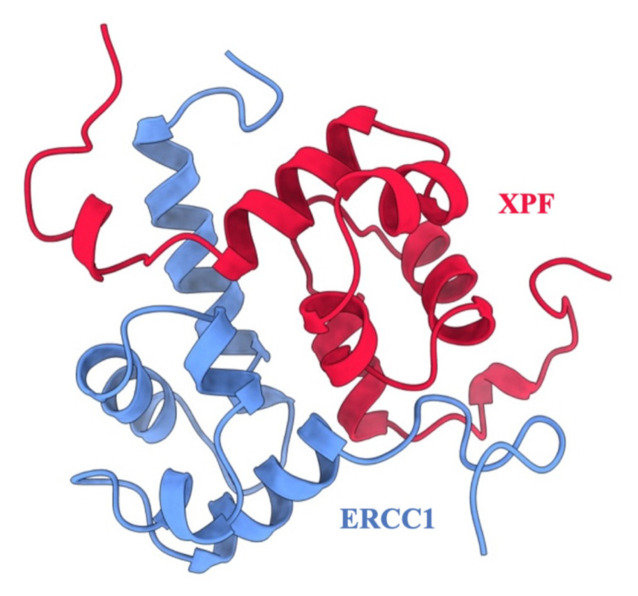
The ERCC1-XPF PPI complex (PDB ID: 1Z00). ERCC1 is shown in blue, and XPF is shown in red.

Investigations into DNA repair proteins have identified the Excision Repair Cross Complementation Group 1 (ERCC1) and Xeroderma Pigmentosum Complementation Group F (XPF) complex as being more highly expressed in cancer cell lines that exhibit chemoresistance (Weeda et al., 1997). It has been shown that downregulated ERCC1/XPF expression levels in nonsmall cell lung cancer, ovarian and breast cancer, resulted in increased cisplatin sensitivity (Kirschner and Melton, 2010). Usanova et al. highlight the heightened sensitivity of testicular germ cell tumors (TGCTs) to Cisplatin, which effectively treats about 80% of metastatic TGCTs, caused by reduced expression levels of ERCC1-XPF, resulting in limited DNA repair caused by drug-induced genotoxic effects. Consequently, recent research has explored inhibiting the interaction between the ERCC1/XPF complex in cancer cells to make them more vulnerable to platinum-based therapy (Usanova et al., 2010). The ERCC1/XPF complex (shown in Figure 1), acting as a structure-specific endonuclease, plays a crucial role in DNA repair processes such as nucleotide excision repair (NER), interstrand crosslink (ICL) repair, and double-strand break (DSB) repair (McNeil and Melton, 2012).

In the process of NER, UV-induced photo-adducts, intrastrand crosslinks, and chemical adducts that hinder DNA metabolism are repaired. The ERCC1/XPF complex cleaves the DNA strand at the 5′ end near the lesion, with a priority on protecting transcribing genes to maintain genomic integrity (Kirschner and Melton, 2010). A collection of proteins comprising transcription factor II H (TFIIH), XPG, RPA, and XPA carry out the NER process (Faridounnia et al., 2018).

Interstrand crosslinks result from covalent modifications of both DNA strands, impeding DNA metabolism. This repair pathway explores the use of ICL-inducing chemotherapeutic agents to target highly replicating cells due to their sensitivity (Niedernhofer et al., 2004). The chemical properties of the ICL formed depend on the crosslinking agent utilized. Among these agents, cisplatin, psoralens, and mitomycin-C are the mostly used therapeutics. They prevent the unwinding of the helix acting as a roadblock down the transcription and replication pathways. DNA lesions formed at the G_0_ or G_1_ stages of the cell cycle can be repairable unless the lesion holds up to the S phase where it will transform into DNA double-strand breaks (DSB) stalling the replication (Rahn et al., 2010; McNeil and Melton, 2012).

DSB, known for their high cytotoxicity (McNeil and Melton, 2012), are caused by various factors like ionizing radiation, radiomimetic drugs, and enzymes involved in DNA recombination and replication. Homologous recombination (HR) and nonhomologous end joining recombination (NHEJ) are the primary DSB repair pathways. ERCC1/XPF’s role involves excising bubble-shaped structures and overhanging single DNA strands (Ahmad et al., 2008). ERCC1 downregulation has been linked to a decrease in single- strand annealing (SSA) efficiency, the excision of nonhomologous tails in targeted gene substitution, and genome integrity (Li et al., 2019).

The interaction between ERCC1 and XPF proteins via their helix-hairpin-helix (HhH)_2_ domain is crucial for the complex’s activity (Choi et al., 2005). Each HhH2 domain consists of five α-helices with essential binding residues. Notably, ERCC1 has an additional α-helix in its N-terminus (Choi et al., 2005). The key residues Phe905 in XPF and Phe293 in ERCC1 are critical for dimerization and complex activity. Leu294 (ERCC1) keeps Phe293 (ERCC1) inside the XPF hydrophobic pocket, and Phe905 (XPF) fits into the ERCC1 hydrophobic pocket during heterodimerization (de Laat et al., 1998; Tripsianes et al., 2005). Additionally, residue mutations in XPF residues Asp687, Asp715, Lys727, and Asp731 have been identified as critical for catalytic activity, while residues Arg689 and Arg726 contribute to residual activity between both proteins (Enzlin and Schärer, 2002; Su et al., 2012).

**Figure 2:**
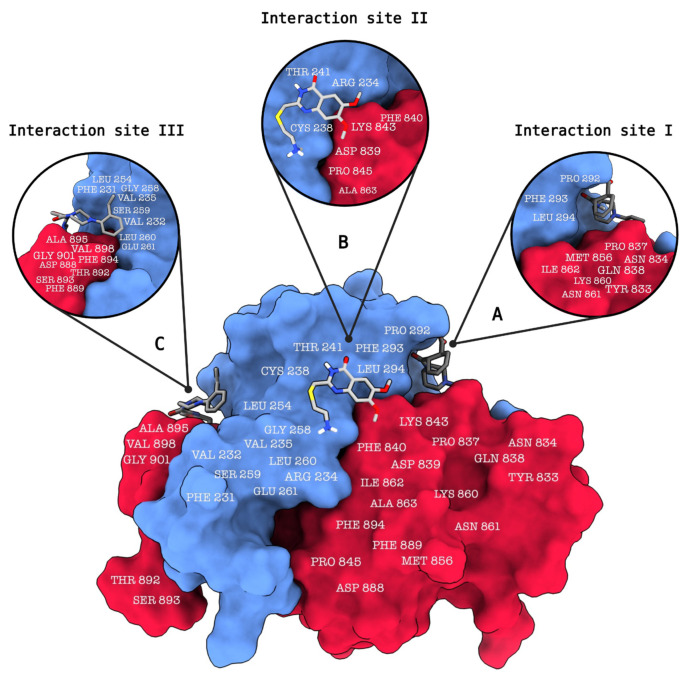
The ERCC1-XPF protein-protein interaction (PPI) complex, (PDB ID: 1Z00) is depicted in this illustration, showcasing ligands bound to three distinct binding sites and the interacting amino acid residues. (a) Binding site 1 comprises a set of residues on the XPF protein, including Tyr833, Asn834, Pro837, Gln838, Met856, Lys860, Asn861, and Ile862, which engage in interactions with corresponding residues 292–294 on the ERCC1 protein. (b) Binding site 2 involves specific residues on the XPF protein, namely Asp839, Phe840, Lys843, Pro845, and Ala863, which interact with residues Arg234, Cys238, and Thr241 on the ERCC1 protein. (c) Binding site 3 encompasses a group of residues on the XPF protein, consisting of Asp888, Phe889, Thr892, Ser893, Phe894, Ala895, Val898, and Gly901. These residues interact with the ERCC1 residues Phe231, Val232, Val235, Leu254, Gly258, Ser259, Leu260, and Glu261. This visual representation was generated using ChimeraX.

In a previously featured study by Jordheim et al., strong interaction between XPF and ERCC1 residues was described, with the exception of Asp839 on XPF, which changed binding interaction energy by approximately 1 kcal/mol (Jordheim et al., 2013). Additionally, Phe293 on ERCC1 was identified as crucial for dimerization interactions with XPF, exhibiting the highest significant reduction to the overall enthalpic contribution of the binding free energy by −11 kcal/mol (Jordheim et al., 2013). The study also identifies three potential ligand binding sites on the ERCC1/XPF complex (Figure 2). The first of these binding sites (site 1) comprises residues Tyr833, Asn834, Pro837, Gln838, Met856, Lys860, Asn861, and Ile862, which interact with ERCC1 residues 292–294 (Jordheim et al., 2013) (Figure 2a). The second identified binding site (site 2) involves residues Asp839, Phe840, Lys843, Pro845, and Ala863, which interact with ERCC1 residues Arg234, Cys238, and Thr241 (Jordheim et al., 2013) (Figure 2b). The third identified binding site entails residues Asp888, Phe889, Thr892, Ser893, Phe894, Ala895, Val898, and Gly901, interacting with ERCC1 residues Phe231, Val232, Val235, Leu254, Gly258, Ser259, Leu260, and Glu261 (Jordheim et al., 2013) (Figure 2c).

**Figure 3:**
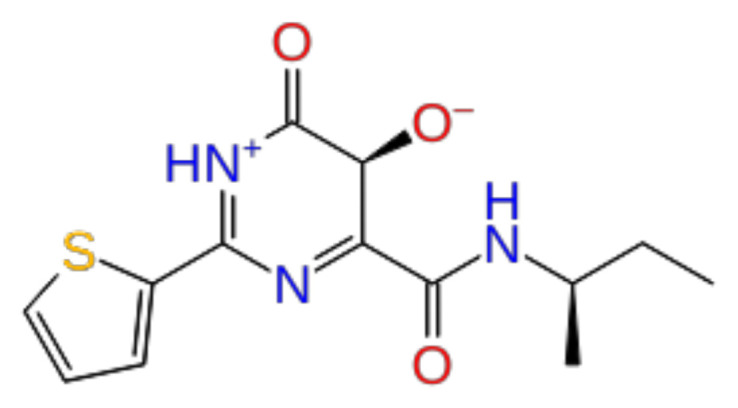
The reference molecule CHEMBL3617209 used in this study.

Previously, a hydroxypyrimidinone derivative (Figure 3), titled compound 22, was proven to have submicromolar half maximal inhibition concentration (IC_50_) via a high-throughput fluorescence-based in vitro biochemical assay (Chapman et al., 2015). The hydroxypyrimidinone scaffold was specifically selected for its ability to bind with metal ions, namely Mg^+2^ and its similarity to the previously identified active endonuclease inhibitor motif N-hydroxyimide (Chapman et al., 2015). This compound was considered as a comparative reference in our study.

## 2. Methodology

### 2.1. Protein preparation

The solution NMR spectroscopy structure of ERCC1/XPF (PDB ID: 1Z00) (Tripsianes et al., 2005) was obtained from the RCSB Protein Data Bank. Jordheim et al. (2013) have previously identified the crucial ERCC1/XPF interface residues based on this pdb file, strongly influencing our choice in choosing this structure amongst the others in particular. The pdb file includes 20 conformers of the ERCC1/XPF protein complex. All conformers were prepared using the Protein Preparation module (Madhavi Sastry et al., 2013) of the Maestro molecular modeling package. The protein was first preprocessed by assigning bond orders, adding hydrogens, creating zero-order bonds to metals, and creating disulfide bonds. The Prime module (Jacobson et al., 2002; Jacobson et al., 2004) was then implemented to complete the missing side chains and loops. Hydrogen bonds were later assigned using PROPKA (Olsson et al., 2011) at pH 7.0, after which the protein was minimized using the OPLS3e forcefield (Harder et al., 2016). Finally, the conformer with the lowest energy was selected for the study.

### 2.2. Receptor grid generation

Receptor grids were defined as the centroids of previously identified binding site residues by Jordheim et al. The same 3 grids with the coordinates mentioned below were used for the ERCC1 chain, ERCC1-XPF protein complex, and the XPF chain. For binding site 1, a grid encompassing XPF residues Tyr833, Asn834, Pro837, Gln838, Met856, Lys860, Asn861, Ile862 was generated. For binding site 2, a grid enclosing XPF residues Asp839, Phe840, Lys843, Pro845, and Ala863 was created. For binding site 3, the grid was created in the presence of XPF residues Asp888, Phe889, Thr892, Ser893, Phe894, Ala895, Val898, Gly901. The corresponding x, y, z Cartesian coordinates of all the grid centers were noted as follows: binding site 1 (9.00, −4.20, −0.34), binding site 2 (0.31, 1.19, −7.36), and binding site 3 (−11.05, 7.43, 1.96). The outer box and inner box sizes were set to 30 Å and 10 Å, respectively. All receptor hydroxyl and thiol groups encapsulated by the grid box were allowed to rotate.

### 2.3. Ligand preparation

Previously discovered ERCC1/XPF inhibitor Compound 22 (Chapman et al., 2015) was retrieved from the ChEMBL database (Zdrazil et al., 2023) noted as CHEMBL3617209. A comprehensive screening library was created from 460,160 compounds from the Enamine Hit Locator Library compounds^1^, 207,525 ChemDiv Representative Library compounds^2^, and 200,165 compounds from the SPECS SC library^3^. The LigPrep tool (Madhavi Sastry et al., 2013) from Maestro was utilized to generate all combinations of possible 3D conformation states via Epik (Shelley et al., 2007; Greenwood et al., 2010) at target pH 7.0 ± 2.0 using the OPLS3e forcefield (Harder et al., 2016).

### 2.4. Virtual screening workflow

The Virtual Screening Workflow (Friesner et al., 2006) module implements 3 methods of docking: High Throughput Virtual Screening (HTVS), Standard Precision (SP), and Extra Precision (XP). The Epik (Shelley et al., 2007; Greenwood et al., 2010) state penalties were used, the docking method was set as flexible and postdocking minimization was applied. Following the HTVS algorithm, 10% of the top-docking scored compounds of all states were automatically selected and passed on to the SP algorithm, after that 10% of the top-scoring compounds were chosen and transferred to the XP docking algorithm where 10% of the best compounds of only top-scoring states were kept for postprocessing with Prime MM/GBSA (Jacobson et al., 2002; Jacobson et al., 2004).

### 2.5. Anticancer therapeutic activity prediction

The filtered compounds were subjected to the MetaDrug/MetaCore cancer therapeutic activity binary-QSAR model^4^ for anticancer activity prediction. This binary-QSAR model is built with the following parameters: Training set N = 886, Test set N = 167, Sensitivity = 0.89, Specificity = 0.83, Accuracy = 0.86, and Matthews correlation coefficient (MCC) = 0.72 (Ekins et al., 2006). Compounds with a predicted activity probability of 0.5 or higher are considered to be potentially active anticancer compounds.

### 2.6. ADME/T studies

To address the drug metabolism and pharmacokinetics of the final potential inhibitors, ADME/T predictions have been conducted via MetaDrug/MetaCore ADME/T QSAR models. MetaDrug/MetaCore ADME parameters filter drug candidates based on their physicochemical and pharmacokinetic properties before they reach the preclinical phase, blood-brain penetration, lipophilicity, human serum protein binding, affinity to human serum albumin, and water solubility. MetaDrug/MetaCore toxicity QSAR models^5^ provide 26 independent toxicity filters that further investigate pharmacokinetic profiles (Sahin, 2022). Details of the model building parameters are provided in the supplementary tables.

### 2.7. Molecular dynamics (MD) simulations

The top-25 molecules with high (in absolute values) docking scores and anticancer activity prediction of 0.5 or higher from each of the three libraries ChemDiv, Enamine, and SPECS were subjected to MD simulations. The ERCC1/XPF complexed with the ligand was immersed in a TIP3P solvent model (Mark and Nilsson, 2001) water box with 10 Å buffering edges. The system was then neutralized by counterion placement and 0.15 M NaCl solution addition. All atoms were configured with the OPLS3e forcefield (Harder et al., 2016). Following the system setup, the MD simulations were run using Desmond ((Bowers et al., 2006)) and the simulation parameters were used as follows: a simulation time of 10 ns, a recording interval of 10 ps, 1000 frames, and the ensemble class NPT which utilizes the Nose-Hoover chain thermostat method (Evans and Holian, 1985; Martyna et al., 1992) at a temperature of 310 K and the Martyna-Tobias-Klein barostat method (Martyna et al., 1994) at a constant pressure of 1.01325 bar and an isotropic coupling style was incorporated to achieve thermodynamic equilibrium. The RESPA integrator (Tuckerman et al., 1992) was implemented every 2.0 femtoseconds (fs). A cutoff of 9 Å radius was assigned for short-range electrostatic and van Der Waals interactions while the long-range interactions were calculated through the particle mesh Ewald method and periodic boundaries. A total of 3 short-MD simulation replicas were run for each compound to procure a total of 30 ns and 3000 frames per compound. These short-MD simulations were proceeded with one longer MD simulation for each of the complexes for 100 ns and a recording interval of 100 ps with the same configurations listed previously. For extended 200 ns simulations, the same configurations were also applied, and 1000 frames per simulation were recorded.

### 2.8. Molecular mechanics with generalized Born and surface area solvation (MM/GBSA)

The average binding energy of ligands to proteins was calculated using the MM/GBSA method integrated in Prime (Jacobson et al., 2002; Jacobson et al., 2004). For each of the mentioned simulations, a total of 100 frames were extracted, of which the average MM/GBSA and standard deviation of the MM/GBSA scores were computed per compound. A total average of the MM/GBSA scores for all 3 MD replicas (300 frames) per compound was then obtained. The MM/GBSA scores were generated based on the VSGB 2.0 implicit solvation model (Li et al., 2011) and the OPLS3e (Harder et al., 2016) forcefield.

### 2.9. Z-score filtration

The Z-score was calculated based on the docking score of the selected top 25 molecules from each library and each grid site, and the normal distribution curve was plotted. The compounds with a minimum of 90% confidence level (z-score of 1.96) were chosen (where not applicable, the top 3 molecules with the highest Z-score were chosen) from each binding site of each library. A total of 28 selected compounds from small molecule libraries were used in long (100 ns and 200 ns) MD simulations in each site together with a reference compound. Thus, for short simulations in total 20.52 μs (10 ns × 25 compounds × 3 libraries × 3 sites × 3 replicas × 3 protein forms (i.e. ERCC1/XPF, ERCC1 only, and XPF only)) + (10 ns × 1 reference molecule × 3 sites × 3 replicas × 3 protein forms), and for long simulations in total 11.1 μs (300 ns × 28 compounds) + (1 reference × 300 ns × 3 protein forms × 3 sites) MD simulations were conducted.

**Figure 4:**
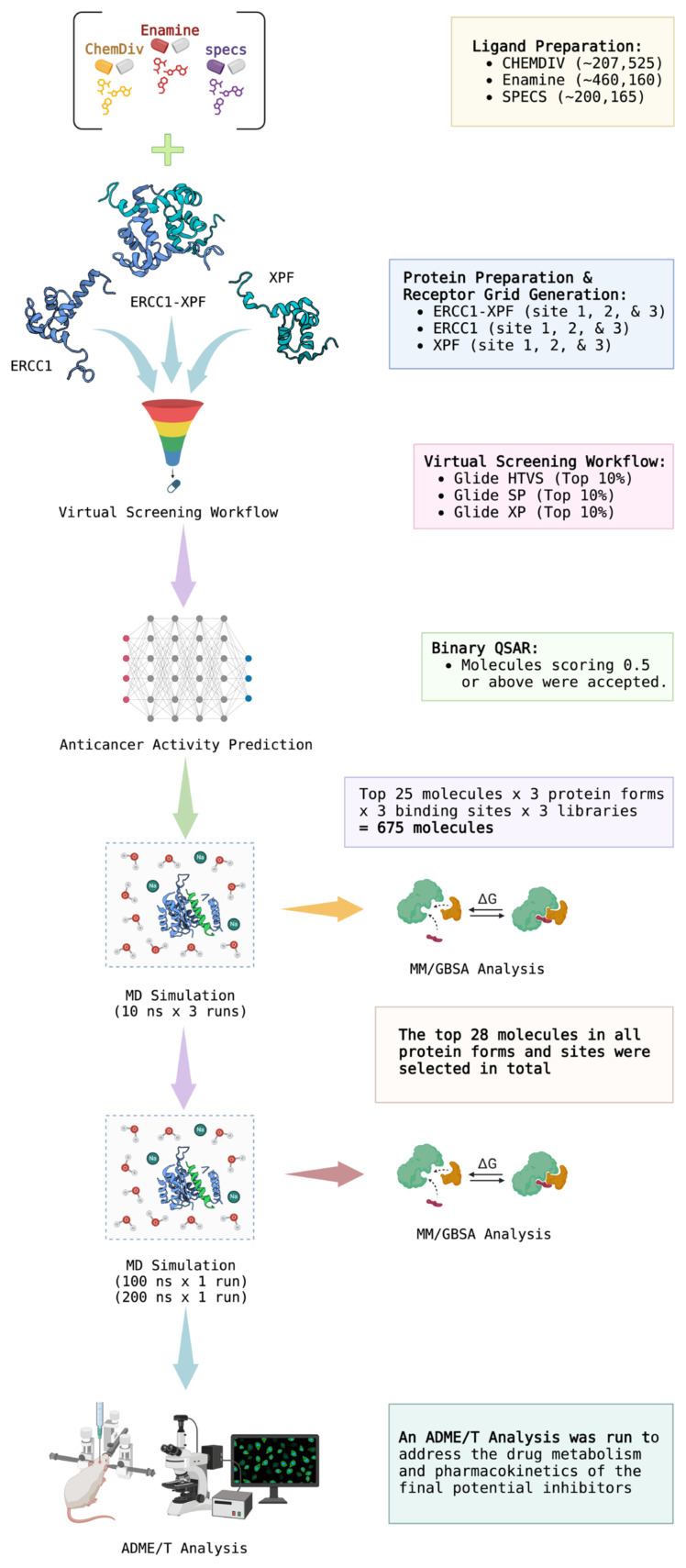
A flowchart of the methodology followed in this study. Created with BioRender.com.

A generalized scheme of work followed in this study is depicted in Figure 4.

## 3. Results and discussion

HTVS implementations offer the advantages of screening ultra-large (approximately 100,000–1,000,000 compounds) chemical libraries within a matter of hours with relatively high accuracy (Salmas et al., 2015). The Glide Virtual Screening Workflow (VSW) algorithm adopts a funnel-like three-staged docking approach, utilizing three docking protocols, HTVS, SP, and XP, respectively, where only a percentage of the results from each protocol is passed on to the next one, enabling a fast and accurate screening of large small-molecule databases (Friesner et al., 2004; Friesner et al., 2006). HTVS performs a robust docking, passing on the top-scoring 10% of compounds to be docked with the SP algorithm, where the resulting ranked postdocking 10% are passed onto the XP algorithm, which also implicates the shape complementarity of the ligand and receptor, eliminating “false positives” passed from the previous approaches (Friesner et al., 2006). Integrating MD simulations into the resulting docking poses aids in the identification of the changing time-dependent dynamics of these molecular systems ranging from quick internal movements to intricate phenomena like protein folding and ligand binding (Santos et al., 2019). We have further qualitatively assessed the binding free energies by postprocessing the MD trajectories by averaging MM/GBSA calculations of collected frames.

**Figure 5:**
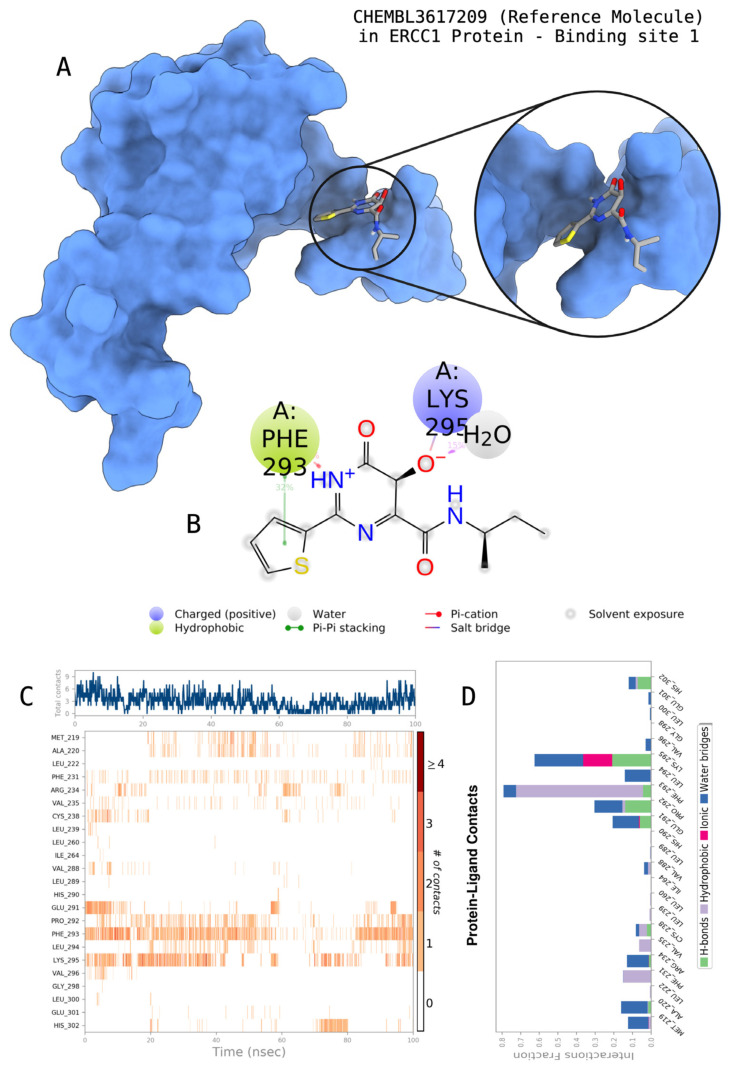
Interactions of the reference molecule CHEMBL3617209 with the critical residues on the ERCC1 binding site 1. Surface representation of CHEMBL3617209 on the ERCC1 (a). Ligand interaction diagram of CHEMBL3617209 with the critical residues mentioned (b). Time-dependent protein-CHEMBL3617209 contact panel throughout 100 ns MD simulations. The top-panel indicates the total contacts, while the bottom-panel indicates the formed and broken interactions (c). Interaction fractions and characterization of interactions of binding pocket residues of the ERCC1 with CHEMBL3617209 throughout MD simulations (d).

**Figure 6:**
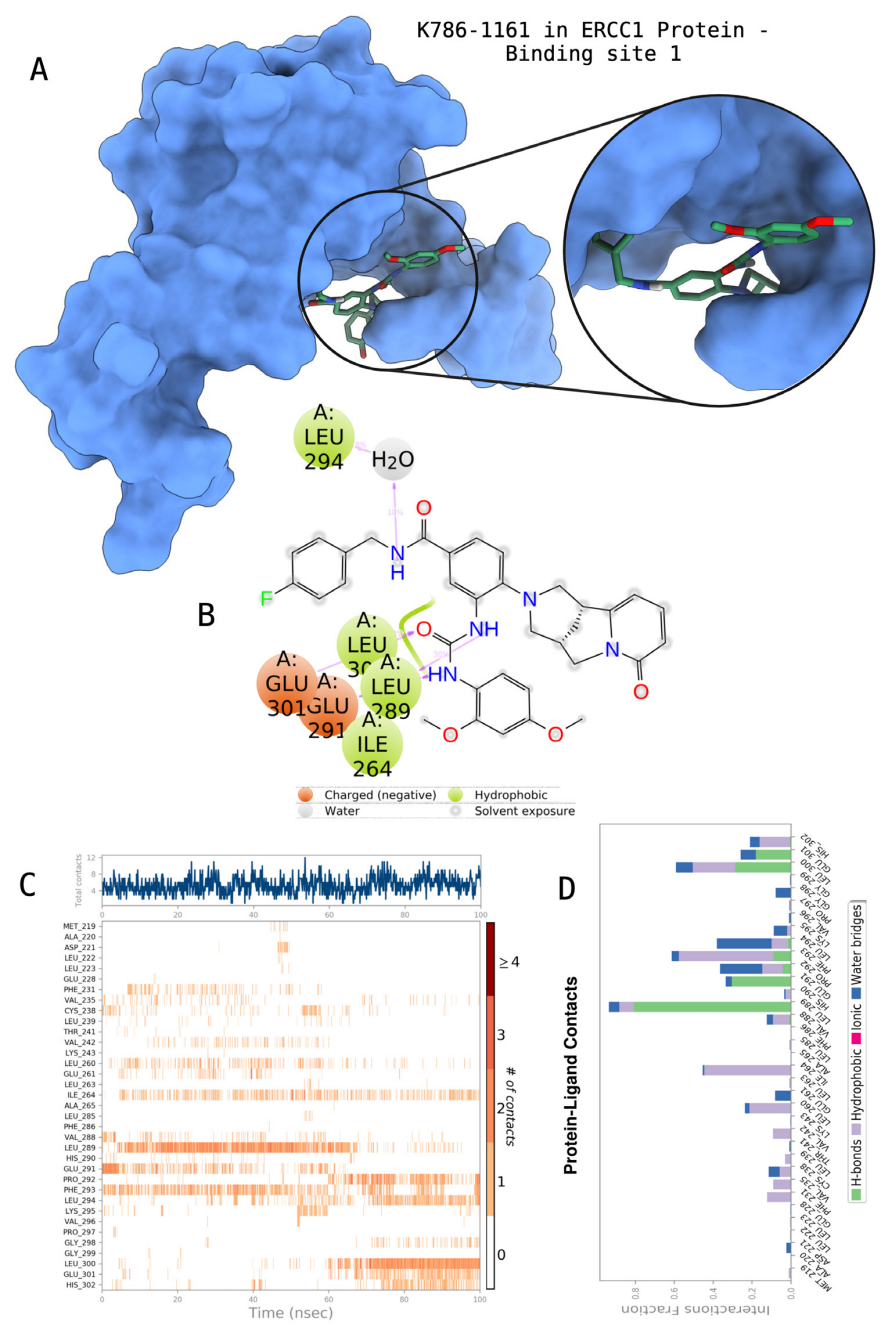
Interactions of compound K786-1161 from the ChemDiv library with the critical residues on the ERCC1 binding site 1. Surface representation of K786-1161 on the ERCC1 (a). Ligand interaction diagram of K786-1161 with critical residues mentioned (b). Time-dependent protein-K786-1161 contact panel throughout 100 ns MD simulations. The top-panel indicates the total contacts, while the bottom-panel indicates the formed and broken interactions (c). Interaction fractions and characterization of interactions of binding pocket residues of the ERCC1 with K786-1161 throughout MD simulations (d).

**Figure 7:**
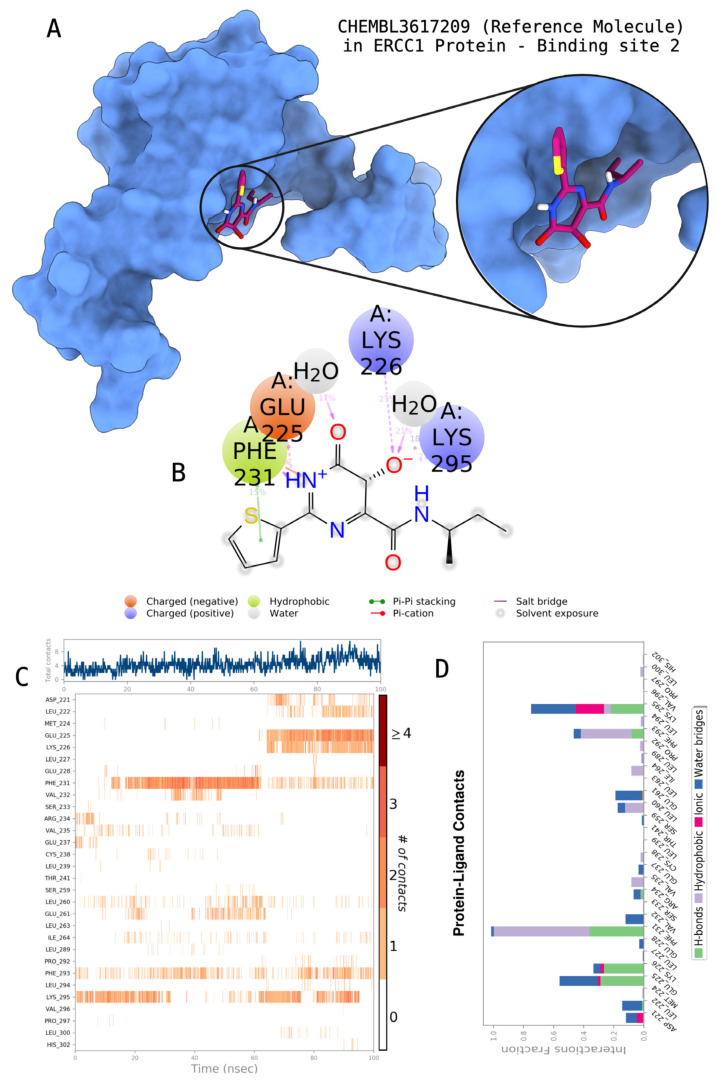
Interactions of the reference molecule CHEMBL3617209 with the critical residues on the ERCC1 binding site 2. Surface representation of CHEMBL3617209 on the ERCC1 (a). Ligand interaction diagram of CHEMBL3617209 with critical residues mentioned (b). Time-dependent protein-CHEMBL3617209 contact panel throughout 100 ns MD simulations. The top-panel indicates the total contacts, while the bottom-panel indicates the formed and broken interactions (c). Interaction fractions and characterization of interactions of binding pocket residues of the ERCC1 with CHEMBL3617209 throughout MD simulations (d).

**Figure 8:**
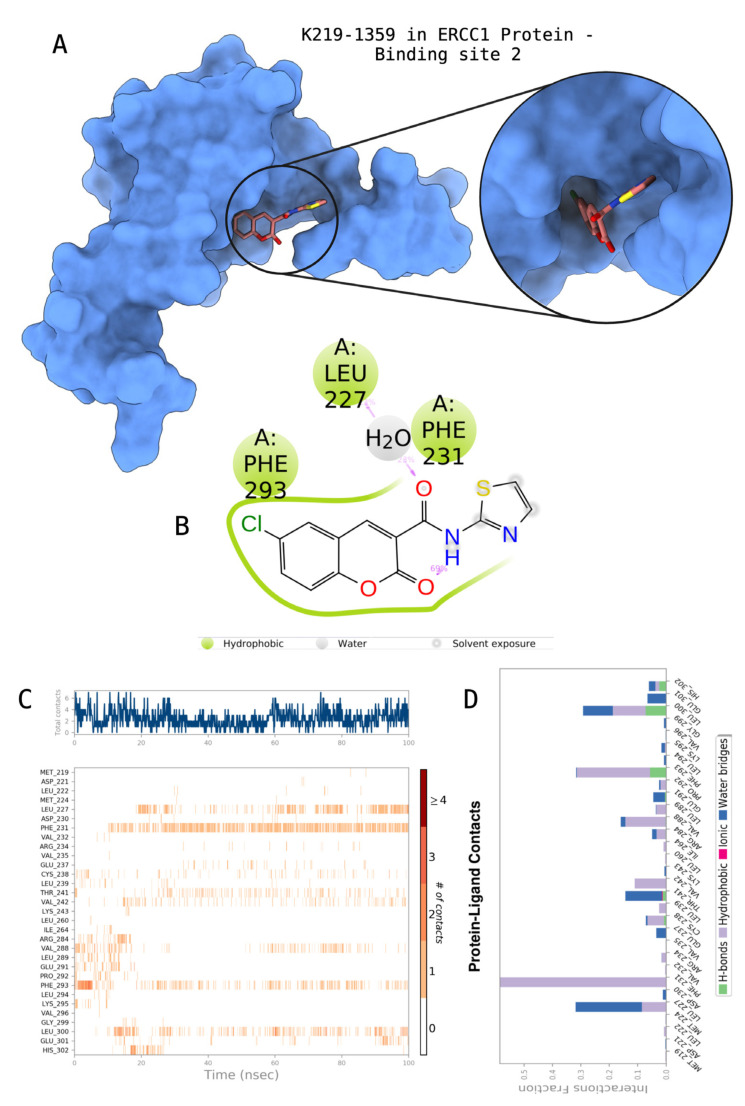
Interactions of compound K219-1359 from the ChemDiv library with the critical residues on the ERCC1 binding site 2. Surface representation of K219-1359 on the ERCC1 (a). Ligand interaction diagram of K219-1359 with critical residues mentioned (b). Time-dependent protein-K219-1359 contact panel throughout 100 ns MD simulations. The top-panel indicates the total contacts, while the bottom-panel indicates the formed and broken interactions (c). Interaction fractions and characterization of interactions of binding pocket residues of the ERCC1 with K219-1359 throughout MD simulations (d).

**Figure 9:**
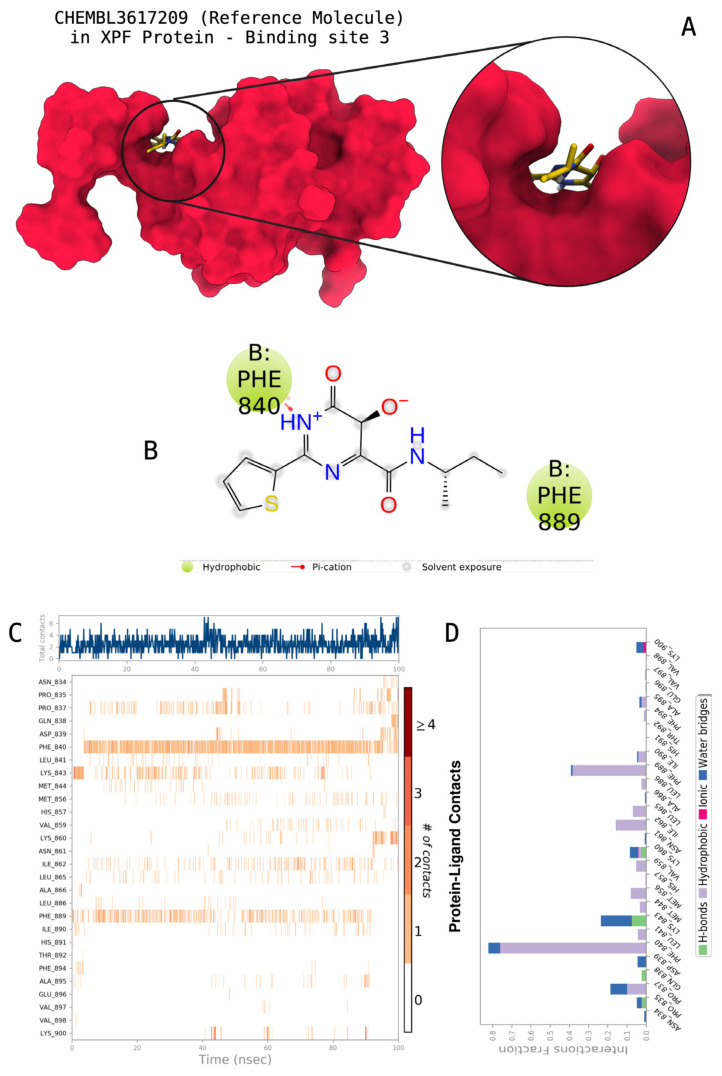
Interactions of the reference molecule CHEMBL3617209 with the critical residues on the XPF binding site 3. Surface representation of CHEMBL3617209 on the XPF (a). Ligand interaction diagram of CHEMBL3617209 with critical residues mentioned (b). Time-dependent protein-CHEMBL3617209 contact panel throughout 100 ns MD simulations. The top-panel indicates the total contacts, while the bottom-panel indicates the formed and broken interactions (c). Interaction fractions and characterization of interactions of binding pocket residues of the XPF with CHEMBL3617209 throughout MD simulations (d).

**Figure 10:**
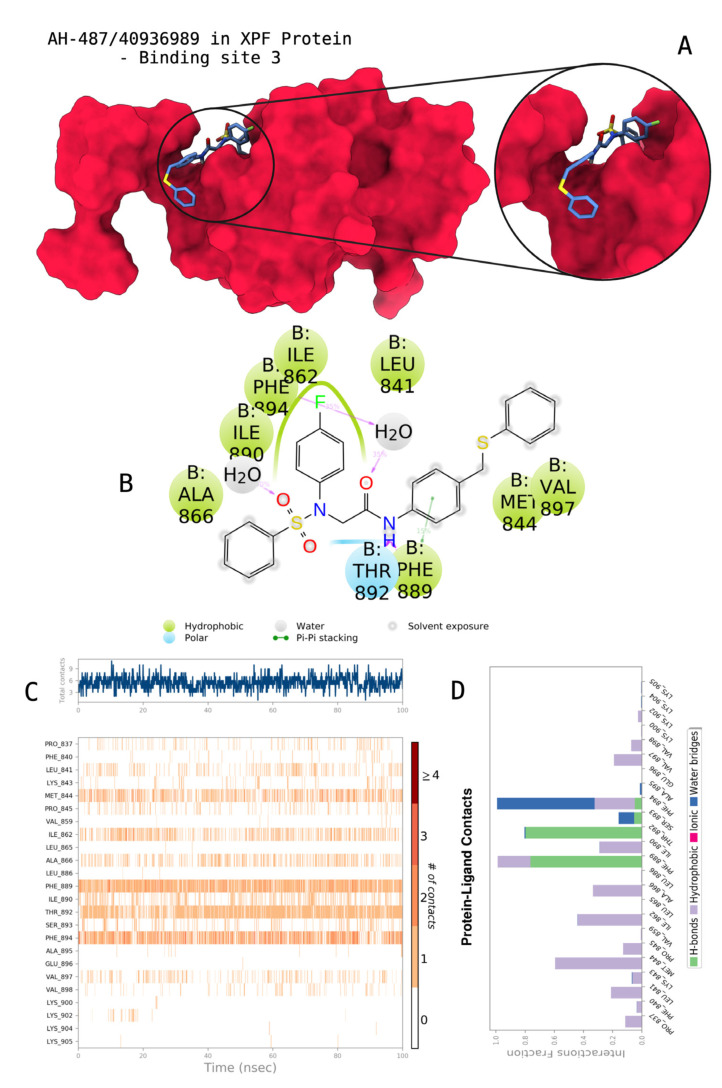
Interactions of compound AH-487/40936989 from the SPECS library with the critical residues on the XPF binding site 3. Surface representation of AH-487/40936989 on the XPF (a). Ligand interaction diagram of AH-487/40936989 with critical residues mentioned (b). Time-dependent protein-AH-487/40936989 contact panel throughout 100 ns MD simulations. The top-panel indicates the total contacts, while the bottom-panel indicates the formed and broken interactions (c). Interaction fractions and characterization of interactions of binding pocket residues of the XPF with AH-487/40936989 throughout MD simulations (d).

The docking scores, average MM/GBSA scores for all mentioned MD simulations, and anticancer therapeutic activity prediction values for the top-4 compounds from each of the grid sites, filtered using our methodology, have been tabulated and are shown in Tables 1, 2, and 3 for the ERCC1, ERCC1-XPF protein complex, and XPF, respectively. Docking scores, average MM/GBSA scores, and anticancer therapeutic activity prediction values of all 28 compounds obtained from each library for every protein and each of the three binding sites along with the reference molecule can be found in the supplementary tables provided. The top-scoring molecule in each binding site was selected and illustrated in comparison with the CHEMBL3617209 reference molecule. K786-1161 from the ChemDiv library in ERCC1 protein binding site 1 showed a 2-fold increase in the post-MD average MM/GBSA score of −80.21 kcal/mol over CHEMBL3617209 with an MM/GBSA score of −41.86 kcal/mol (Table 1). The surface representation of CHEMBL3617209 at binding site 1 on the ERCC1 protein is depicted in Figure 5a. CHEMBL3617209 exhibits hydrophobic contacts with ERCC1 residue Phe293, and this interaction is strengthened with the addition of a sidechain interaction with Lys295 (Figure 5b). These contacts are maintained mostly throughout 100 ns of MD simulations (Figure 5c). Interactions are mostly characterized by water-bridges and hydrophobic contacts (Figure 5d). The surface representation of K786-1161 at binding site 1 on the ERCC1 protein is displayed in Figure 6a. K786-1161 also exhibits hydrophobic contacts with ERCC1 residues in its vicinity, namely Leu300, Leu289, Leu294, and Ile264 (Figure 6b). These hydrophobic contacts are fortified with charged sidechain interactions of Glu291 and Glu301 (Figure 6b). The hydrogen bonds with these charged sidechains appear in the second 50 ns of the simulations and reinforce the position of the ligand in the binding pocket (Figure 6c). The fraction of interaction of hydrogen bonds with respect to other interactions made with K786-1161 and the protein complex is shown to be higher than any other contact (Figure 6d). K219-1359 from the Enamine library in site 2 showed a high overall MM/GBSA score (−57.56 kcal/mol) when compared to reference CHEMBL3617209 which abandoned the binding pocket after 100 ns of simulation (Table 1). The surface representation of CHEMBL3617209 at binding site 2 on the ERCC1 protein is depicted in Figure 7a. Reference CHEMBL3617209 interacts with hydrophobic ERCC1 Phe231 through π-π stacking interactions and makes hydrogen bonds with charged ERCC1 residues Lys226, Lys295, and Glu225 (Figure 7b). These interactions are sustained interchangeably and partially maintained for 100 ns of MD simulations (Figure 7c). Figure 7d reemphasizes the interactions mentioned in Figure 7c. The surface representation of K219-1359 at binding site 2 on the ERCC1 protein is shown in Figure 8a. K219-1359 exhibits mainly hydrophobic interactions with ERCC1 Phe293, Leu227, and Phe231 (Figure 8b). Hydrophobic contacts with Phe231 were maintained mostly throughout the MD simulation and contacts with Phe293 and Leu300 were maintained interchangeably (Figure 8c). Interactions mentioned in Figure 8b along with additional contacts with Asp230 are further highlighted in Figure 8d. AN-487/40936989 from the SPECS SC library in the XPF protein binding site 3 shows an average MM/GBSA score of −89.7 kcal/mol as opposed to CHEMBL3617209 which leaves the binding pocket after 100 ns of simulations (Table 2). The surface representation of CHEMBL3617209 at binding site 3 on the XPF protein is depicted in Figure 9a. It can be observed that reference CHEMBL3617209 is maintained in site 3 through mostly hydrophobic contacts with XPF residues Phe840 and Phe889 (Figure 9b). These hydrophobic contacts make up most of the interactions of the protein-ligand complex during the 100 ns MD simulations (Figures 9c and 9d). The surface representation of AN-487/40936989 at binding site 3 on the XPF protein is represented in Figure 10a. AN-487/40936989 shows hydrophobic interactions with Leu841, Met844, Ile862, Ala866, Phe889, Ile890, Phe894, and Val897 of the XPF target (Figure 10b). These interactions, especially Phe889, Phe894, and Thr892 are maintained during the 100 ns simulations (Figure 10c). Namely highlighted interactions are hydrogen-bonding with Phe889 and Thr892 with additional water-bridge contacts with Phe894 (Figure 10d).

The average MM/GBSA scores of the short MD (10 ns) simulations and the long MD (100 ns) simulations show consistency which reflects a positive outlook for ligand stability in lengthier MD simulations (Tables 1, 2, and 3). Site 2 shows an overall lower average binding free energy when compared to sites 1 and 3, which is mainly due to its mostly hydrophobic character. Furthermore, the production run of MD simulations was extended with an extra 200 ns in order to see the effect of the extension of the simulations to the structural and dynamical properties of the used systems (Tables 1, 2, and 3). Average MM/GBSA calculations for the reference molecule at binding sites 2 and 3 were not conducted, due to the unbinding of the molecule after 100 ns at these sites. Average MM/GBSA scores of the reference molecule at site 1, however, do not indicate a substantial change from average MM/GBSA scores calculated over 100 ns of MD simulations. However, the selected compounds show predicted high affinity and are maintained in their respective binding pockets.

Therapeutic activity predictions obtained from the MetaCoreMetaDrug platform (see Supplementary Tables) have shown that reference CHEMBL3617209 is a highly active (0.83) anticancer molecule. Chosen compounds from the selected libraries also display high anticancer therapeutic activities (> = 0.70), since scores > = 0.5 are considered active anticancer molecules. Here, the docking scores, and average MM/GBSA scores show that the selected compounds are also more selective than CHEMBL3617209 to ERCC1 and XPF, and also exhibit anticancer properties.

## 4. Conclusions

The importance of the inhibition of the ERCC1/XPF binding stems from its inclusion and crucial role in DNA repair mechanisms induced by chemotherapeutic drugs. The current study focuses on the search for ERCC1/XPF binding inhibitors for the treatment of chemotherapy resistance. Here, three large-scale chemical compound libraries have been screened with a vigorous docking-based algorithm and binary-QSAR-based filtering. Results were further quantified with MD simulations and post-MD average MM/GBSA calculations. The resulting compounds have (i) better average binding free energy scores and (ii) are active as anticancer molecules through computational assessment. Our approach combines docking, QSAR, MD, and binding free energy calculations providing an accurate assessment of potential selective ERCC1/XPF protein-protein interaction inhibitors.

## Supplementary Information



## Figures and Tables

**Table 1:** Docking scores, average MM/GBSA scores, and anticancer therapeutic activity prediction values for top-scoring compounds in the ERCC1 protein binding sites 1, 2, and 3.

ERCC1												
	2D Structure	Ligand ID	Library	Anticancer activity prediction	Docking score (kcal/mol)	Post-VSW MM/GBSA average score (kcal/mol)	Short MD (10 ns) MM/GBSA average score (kcal/mol)	STDEV (10 ns)	Long MD (100 ns) MM/GBSA average score (kcal/mol)	STDEV (100 ns)	Long MD (200 ns) MM/GBSA average score (kcal/mol)	STDEV (200 ns)
**Site 1**	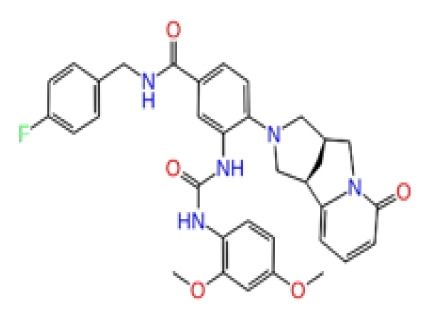	K786-1161	CHEMDIV	0.74	−6.26	−61.71	−73.77	9.36	−80.21	10.03	−92.09	14.94
	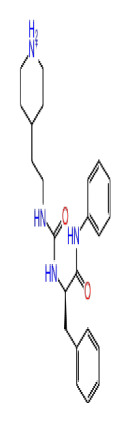	Z2482664935	Enamine	0.77	−7.01	−68.83	−76.40	6.96	−78.12	6.51	−81.05	7.58
	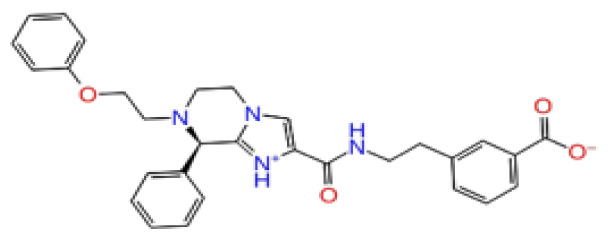	ZC46-0211	CHEMDIV	0.62	−6.65	−62.82	−73.10	6.46	−67.69	9.85	−69.30	9.55
	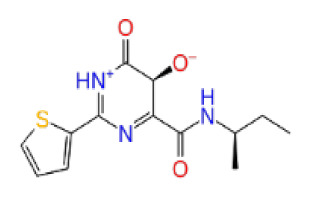	CHEMBL3617209	CHEMBL	0.83	−2.97	−31.33	−29.34	7.92	−41.86	5.69	−40.08	6.00
**Site 2**	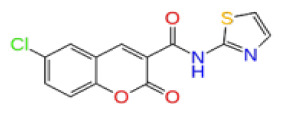	K219-1359	CHEMDIV	0.51	−5.40	−46.36	−56.49	6.40	−57.56	4.23	−61.11	7.03
	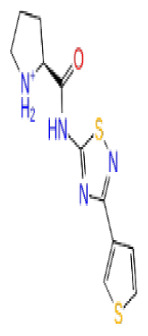	Z1450326974	Enamine	0.69	−5.56	−40.27	−57.03	7.67	−62.32	5.29	−60.70	5.30
	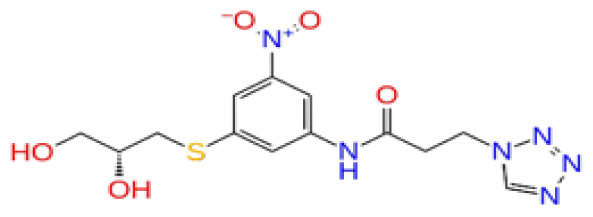	Y501-9249	CHEMDIV	0.74	−5.24	−42.34	−61.38	7.34	−54.34	8.26	−52.51	7.21
	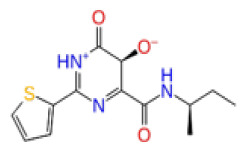	CHEMBL3617209	CHEMBL	0.69	−4.72	−29.43	−39.10	7.29	−44.12	6.75	NA	NA
**Site 3**	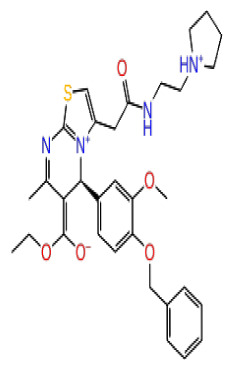	V019-9483	CHEMDIV	0.64	−5.92	−52.11	−57.71	9.10	−70.97	13.99	−66.36	11.47
	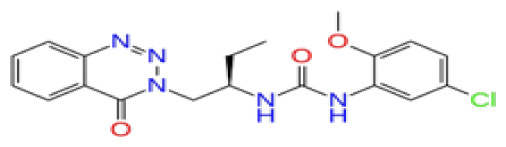	D264-0862	CHEMDIV	0.58	−5.85	−58.31	−60.17	4.48	−61.98	5.05	−61.44	6.00
	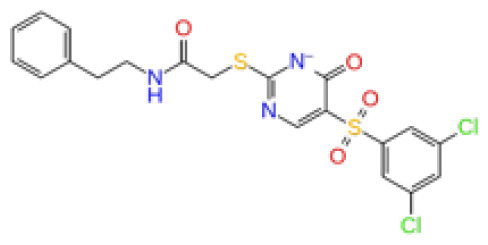	G605-0598	CHEMDIV	0.71	−5.71	−62.81	−60.36	6.14	−61.88	10.87	−55.30	10.53
	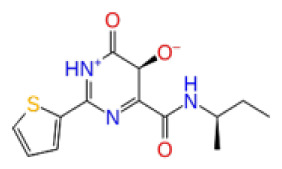	CHEMBL3617209	CHEMBL	0.83	−2.97	−31.33	−29.34	4.06	−28.34	4.18	NA	NA

**Table 2:** Docking scores, average MM/GBSA scores, and anticancer therapeutic activity prediction values for top-scoring compounds in the ERCC1-XPF PPI binding sites 1, 2, and 3.

ERCC1-XPF												
	2D Structure	Ligand ID	Library	Anticancer activity prediction	Docking score (kcal/mol)	Post-VSW MM/GBSA average score (kcal/mol)	Short MD (10 ns) MM/GBSA average score (kcal/mol)	STDEV (10 ns)	Long MD (100 ns) MM/GBSA average score (kcal/mol)	STDEV (100 ns)	Long MD (200 ns) MM/GBSA average score (kcal/mol)	STDEV (200 ns)
**Site 1**	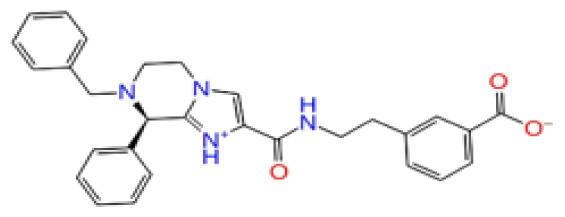	ZC46-0199	CHEMDIV	0.63	−5.95	−51.73	−66.81	5.61	−59.20	4.97	−54.68	6.08
	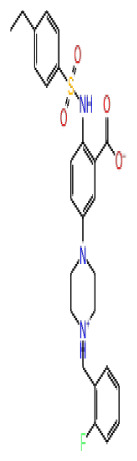	F684-0404	CHEMDIV	0.56	−5.91	−41.52	−52.18	5.25	−46.06	5.13	−44.89	4.43
	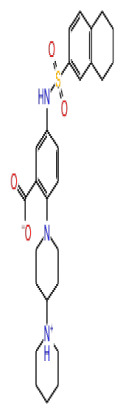	F294-0607	CHEMDIV	0.56	−6.41	−36.94	−50.27	5.87	−42.25	15.21	−42.86	12.00
	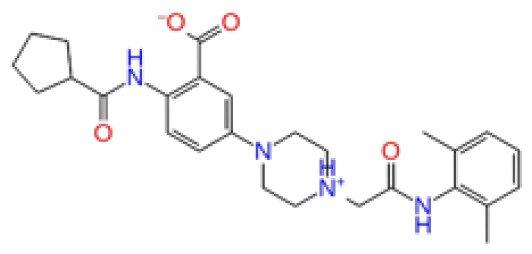	F687-1384	CHEMDIV	0.56	−6.76	−41.39	−53.55	5.00	−34.51	16.45	−35.36	15.65
	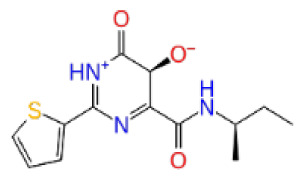	CHEMBL3617209	CHEMBL	0.83	−4.33	−33.70	−31.11	5.05	−33.44	7.20	−33.10	7.47
**Site 2**	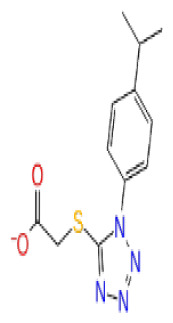	Z74543901	Enamine	0.60	−4.29	−22.93	−31.76	5.93	−44.54	6.70	−45.89	5.27
	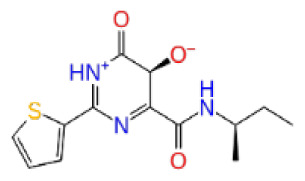	CHEMBL3617209	CHEMBL	0.83	−3.86	−20.02	−22.58	7.57	−39.07	9.63	−39.17	7.44
	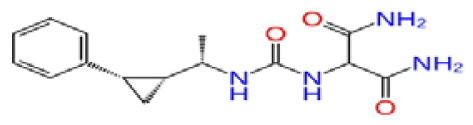	Z807707434	Enamine	0.71	−3.85	−38.43	−31.06	7.79	−30.71	5.17	−31.28	9.18
	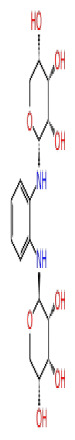	0527-0155	CHEMDIV	0.72	−5.75	−37.63	−37.15	5.52	−25.80	11.15	−23.69	10.97
**Site 3**	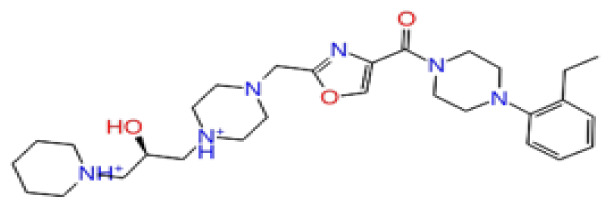	V008-1978	CHEMDIV	0.64	−6.23	−60.16	−63.54	6.31	−61.69	8.73	−64.17	8.76
	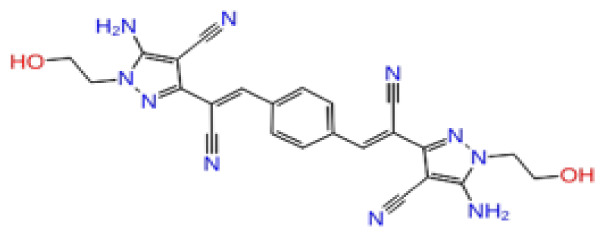	AG-205/36628032	SPECS	0.65	−5.65	−48.58	−63.06	9.43	−58.71	7.56	−60.10	7.14
	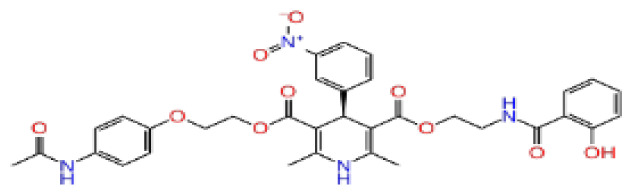	AQ-405/42300197	SPECS	0.59	−5.59	−58.27	−63.27	8.40	−37.39	10.00	−43.40	12.62
	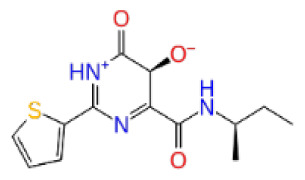	CHEMBL3617209	CHEMBL	0.83	−3.90	−18.75	−30.32	6.69	−41.68	9.19	−43.06	8.02

**Table 3:** Docking scores, average MM/GBSA scores, and anti-cancer therapeutic activity prediction values for top-scoring compounds in the XPF protein binding sites 1, 2, and 3.

XPF												
	2D Structure	Ligand ID	Library	Anticancer Activity Prediction	Docking Score (kcal/mol)	Post-VSW MM/GBSA Average Score (kcal/mol)	Short MD (10 ns) MM/GBSA Average Score (kcal/mol)	STDEV (10 ns)	Long MD (100 ns) MM/GBSA Average Score (kcal/mol)	STDEV (100 ns)	Long MD (200 ns) MM/GBSA Average Score (kcal/mol)	STDEV (200 ns)
**Site 1**	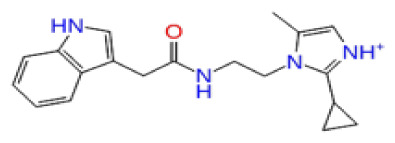	S751-0309	CHEMDIV	0.76	−7.68	−69.28	−77.41	6.94	−73.60	6.30	−71.82	7.64
	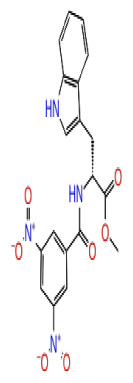	AF-399/33695064	SPECS	0.71	−6.74	−62.60	−73.71	8.58	−68.87	7.96	−65.29	8.42
	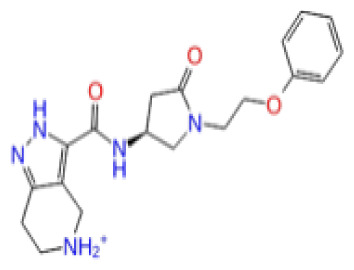	Z1780715778	Enamine	0.67	−7.34	−76.95	−78.16	5.78	−62.79	11.10	−61.72	10.72
	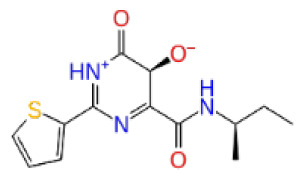	CHEMBL3617209	CHEMBL	0.83	−4.19	−43.98	−52.86	5.38	−53.35	5.19	−52.53	6.22
**Site 2**	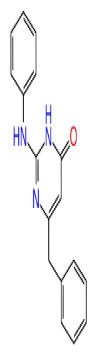	Y020-2805	CHEMDIV	0.70	−5.72	−50.37	−55.27	5.97	−58.28	9.26	−56.98	9.24
	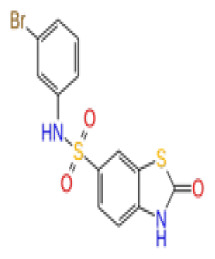	F818-0233	CHEMDIV	0.76	−5.35	−50.98	−56.34	6.82	−53.13	9.18	−54.10	8.24
	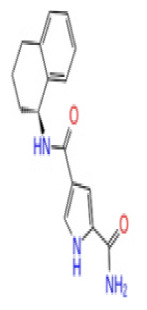	Z605272712	Enamine	0.70	−5.88	−41.79	−55.18	7.22	−46.93	6.31	−44.51	5.77
	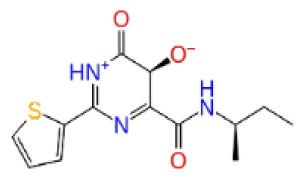	CHEMBL3617209	CHEMBL	0.83	−3.00	−28.61	−33.98	7.38	−53.92	16.30	NA	NA
**Site 3**	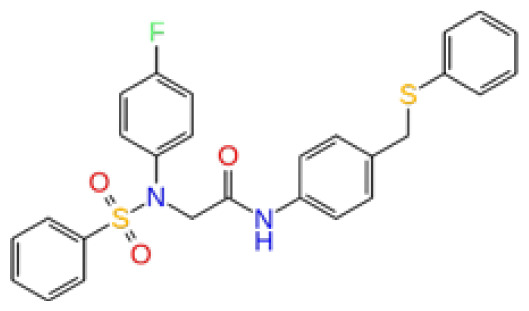	AH-487/40936989	SPECS	0.53	−7.09	−76.90	−81.14	8.66	−96.65	6.78	−97.30	6.66
	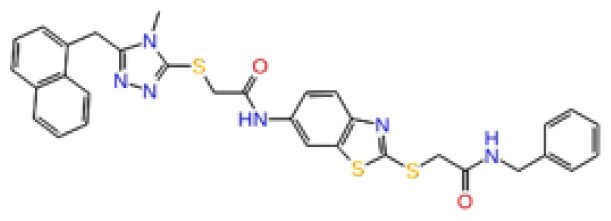	AN-988/40787604	SPECS	0.70	−7.51	−82.64	−86.60	8.12	−89.70	8.64	−87.94	10.33
	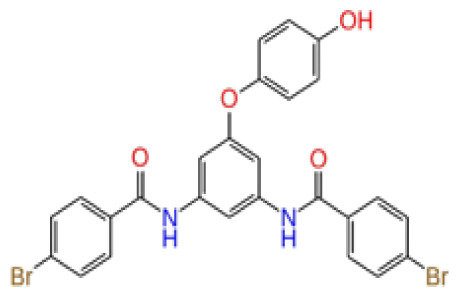	Y500-0018	CHEMDIV	0.71	−7.82	−81.60	−82.48	6.51	−85.87	7.47	−86.17	8.63
	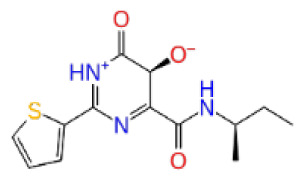	CHEMBL3617209	CHEMBL	0.83	−2.65	−38.50	−31.32	7.99	−44.20	4.75	NA	NA

## Footnotes

1 https://enamine.net/compound-libraries/diversity-libraries [accessed 12 May 2022].

2 https://www.chemdiv.com/catalog/diversity-libraries/bemis-murcko-clustering-library [accessed 12 May 2022].

3 https://www.specs.net [accessed 12 May 2022].

4
https://portal.genego.com/

5
https://portal.genego.com/
