# Supplementary material for: A novel algorithm for the virtual screening of extensive small molecule libraries against ERCC1/XPF protein-protein interaction for the identification of resistance-bypassing potential anticancer molecules
Source: Turk J Biol. 2024 Apr 3;48(2):91–111. doi: 10.55730/1300-0152.2686 (PMC11265927; doi:10.55730/1300-0152.2686)
Supplement: Supplementary file 1 [file TJBio_ERCC1_XPF_Supplementary.docx]

**Supporting Information for:**

**A Novel Algorithm for the Virtual Screening of Extensive Small Molecule Libraries Against ERCC1/XPF Protein-Protein Interaction (PPI) for the Identification of Therapeutic Resistance-Bypassing Small Anticancer Molecules**

Salma Ghazy ^1,2^, Lalehan Oktay^1,2^ and Serdar Durdağı ^1,2,3^*

**Table of Contents**

[**Table S 1:** The predicted anticancer activity, docking scores, post-virtual screening workflow average MM/GBSA scores, and the average MM/GBSA scores of the 3 short MD runs performed on the top 25 molecules obtained from each library in the ERCC1 protein’s binding site 1 3](#_Toc164171921)

[**Table** **S 2:** The predicted anticancer activity, docking scores, post-virtual screening workflow average MM/GBSA scores, and the average MM/GBSA scores of the 3 short MD runs performed on the top 25 molecules obtained from each library in the ERCC1 protein’s binding site 2 5](#_Toc164171922)

[**Table S 3:** The predicted anticancer activity, docking scores, post-virtual screening workflow average MM/GBSA scores, and the average MM/GBSA scores of the 3 short MD runs performed on the top 25 molecules obtained from each library in the ERCC1 protein’s binding site 3 7](#_Toc164171923)

[**Table S 4:** The predicted anticancer activity, docking scores, post-virtual screening workflow average MM/GBSA scores, and the average MM/GBSA scores of the 3 short MD runs performed on the top 25 molecules obtained from each library in the ERCC1/XPF protein complex’s binding site 1 9](#_Toc164171924)

[**Table S 5:** The predicted anticancer activity, docking scores, post-virtual screening workflow average MM/GBSA scores, and the average MM/GBSA scores of the 3 short MD runs performed on the top 25 molecules obtained from each library in the ERCC1/XPF protein complex’s binding site 2 11](#_Toc164171925)

[**Table S 6:** The predicted anticancer activity, docking scores, post-virtual screening workflow average MM/GBSA scores, and the average MM/GBSA scores of the 3 short MD runs performed on the top 25 molecules obtained from each library in the ERCC1/XPF protein complex’s binding site 3 13](#_Toc164171926)

[**Table S 7:** The predicted anticancer activity, docking scores, post-virtual screening workflow average MM/GBSA scores, and the average MM/GBSA scores of the 3 short MD runs performed on the top 25 molecules obtained from each library in the XPF protein’s binding site 1 15](#_Toc164171927)

[**Table S 8:** The predicted anticancer activity, docking scores, post-virtual screening workflow average MM/GBSA scores, and the average MM/GBSA scores of the 3 short MD runs performed on the top 25 molecules obtained from each library in the XPF protein’s binding site 2 17](#_Toc164171928)

[**Table S 9:** The predicted anticancer activity, docking scores, post-virtual screening workflow average MM/GBSA scores, and the average MM/GBSA scores of the 3 short MD runs performed on the top 25 molecules obtained from each library in the XPF protein’s binding site 3 19](#_Toc164171929)

[**Table S 10:** The ADME/Toxicity values of the 28 hit molecules obtained from this study in addition to the reference molecule “CHEMBL3617209” performed by MetaDrug/MetaCore. 22](#_Toc164171930)

[**Table S 11:** MetaDrug/MetaCore parameters of ADME QSAR Models 26](#_Toc164171931)

[**Table S 12:** MetaDrug/MetaCore Parameters of Prediction of Toxic Effects 27](#_Toc164171932)

**References..**……………………………………………………………………………….…………………………………………………………..…34

Table S 1: The predicted anticancer activity, docking scores, post-virtual screening workflow average MM/GBSA scores, and the average MM/GBSA scores of the 3 short MD runs performed on the top 25 molecules obtained from each library in the ERCC1 protein’s binding site 1

| **Ligand ID** | **Library** | **Anticancer Activity Prediction** | **Docking Score (kcal/mol)** | **Post-VSW MM/GBSA (kcal/mol)** | **MM/GBSA Run 1 (kcal/mol)** | **Standard Deviation Run 1** | **MM/GBSA Run 2 (kcal/mol)** | **Standard Deviation Run 2** | **MM/GBSA Run 3 (kcal/mol)** | **Standard Deviation Run 3** | **MMGBSA Average Score (kcal/mol)** | **Average Standard Deviation** | **Normal Distribution** | **Z-score** |
| --- | --- | --- | --- | --- | --- | --- | --- | --- | --- | --- | --- | --- | --- | --- |
| Z2482664935 | Enamine | 0.77 | -7.01 | -68.83 | -77.74 | 5.92 | -80.19 | 9.24 | -71.27 | 5.71 | -76.40 | 6.96 | 0.00 | -2.18 |
| K786-1161 | CHEMDIV | 0.74 | -6.26 | -61.71 | -73.08 | 10.11 | -76.86 | 8.76 | -71.39 | 9.21 | -73.77 | 9.36 | 0.00 | -1.96 |
| ZC46-0211 | CHEMDIV | 0.62 | -6.65 | -62.82 | -72.18 | 6.48 | -59.10 | 5.11 | -88.01 | 7.80 | -73.10 | 6.46 | 0.01 | -1.91 |
| Z125777780 | Enamine | 0.61 | -6.71 | -61.19 | -73.96 | 8.76 | -66.54 | 4.18 | -67.83 | 4.66 | -69.44 | 5.86 | 0.01 | -1.61 |
| V029-1692 | CHEMDIV | 0.71 | -6.24 | -63.45 | -71.94 | 4.61 | -65.26 | 10.60 | -70.08 | 4.04 | -69.09 | 6.42 | 0.01 | -1.59 |
| Z1410469704 | Enamine | 0.73 | -6.87 | -56.92 | -58.77 | 4.59 | -76.39 | 6.94 | -70.28 | 6.56 | -68.48 | 6.03 | 0.01 | -1.54 |
| Z2439024202 | Enamine | 0.71 | -7.59 | -62.67 | -64.19 | 4.29 | -68.37 | 4.58 | -70.55 | 6.06 | -67.71 | 4.98 | 0.01 | -1.47 |
| 5645-0401 | CHEMDIV | 0.75 | -6.24 | -56.65 | -55.16 | 8.44 | -47.61 | 7.69 | -93.01 | 16.44 | -65.26 | 10.86 | 0.01 | -1.28 |
| G857-0408 | CHEMDIV | 0.65 | -6.40 | -49.93 | -72.37 | 12.49 | -61.78 | 7.80 | -61.33 | 8.26 | -65.16 | 9.52 | 0.01 | -1.27 |
| AN-465/42784210 | SPECS | 0.68 | -6.10 | -49.93 | -52.89 | 9.53 | -59.92 | 9.96 | -77.44 | 10.03 | -63.42 | 9.84 | 0.02 | -1.13 |
| AO-476/43407410 | SPECS | 0.74 | -5.91 | -57.29 | -61.34 | 8.08 | -70.13 | 8.08 | -58.72 | 5.57 | -63.40 | 7.25 | 0.02 | -1.13 |
| F531-0552 | CHEMDIV | 0.51 | -6.30 | -53.74 | -63.10 | 10.71 | -54.30 | 10.73 | -66.80 | 8.33 | -61.40 | 9.92 | 0.02 | -0.96 |
| AN-465/42888094 | SPECS | 0.55 | -6.54 | -51.90 | -50.22 | 6.54 | -70.71 | 8.44 | -61.40 | 10.28 | -60.78 | 8.42 | 0.02 | -0.91 |
| AO-022/43513700 | SPECS | 0.85 | -6.05 | -55.40 | -67.22 | 4.76 | -56.31 | 11.48 | -58.22 | 3.90 | -60.58 | 6.71 | 0.02 | -0.90 |
| AN-465/43411131 | SPECS | 0.53 | -6.29 | -62.46 | -59.60 | 6.03 | -59.56 | 4.86 | -62.42 | 5.56 | -60.52 | 5.48 | 0.02 | -0.89 |
| AN-465/43384140 | SPECS | 0.72 | -6.36 | -50.61 | -58.56 | 5.68 | -57.97 | 4.87 | -61.30 | 5.64 | -59.28 | 5.40 | 0.02 | -0.79 |
| AN-465/43411115 | SPECS | 0.28 | -5.88 | -49.93 | -70.61 | 7.50 | -55.43 | 6.87 | -51.56 | 6.53 | -59.20 | 6.97 | 0.02 | -0.79 |
| AN-465/42888093 | SPECS | 0.57 | -5.96 | -59.60 | -52.89 | 9.93 | -59.95 | 7.75 | -64.71 | 6.98 | -59.18 | 8.22 | 0.02 | -0.79 |
| AN-465/42784320 | SPECS | 0.64 | -6.11 | -52.03 | -62.38 | 5.80 | -58.77 | 7.90 | -55.53 | 5.64 | -58.90 | 6.45 | 0.02 | -0.76 |
| G217-1151 | CHEMDIV | 0.77 | -6.78 | -59.77 | -65.89 | 7.58 | -57.63 | 7.36 | -51.09 | 7.94 | -58.20 | 7.63 | 0.03 | -0.71 |
| Z1372397016 | Enamine | 0.62 | -7.38 | -52.12 | -55.14 | 5.18 | -63.06 | 6.81 | -56.08 | 6.20 | -58.09 | 6.06 | 0.03 | -0.70 |
| 4606-0673 | CHEMDIV | 0.63 | -6.56 | -55.45 | -60.68 | 6.64 | -54.03 | 4.50 | -57.54 | 7.19 | -57.41 | 6.11 | 0.03 | -0.64 |
| C450-0313 | CHEMDIV | 0.57 | -6.36 | -60.02 | -65.40 | 8.73 | -47.22 | 11.81 | -56.27 | 5.47 | -56.30 | 8.67 | 0.03 | -0.55 |
| Z649823630 | Enamine | 0.70 | -6.55 | -51.25 | -57.20 | 6.21 | -48.95 | 10.78 | -60.37 | 7.38 | -55.50 | 8.12 | 0.03 | -0.49 |
| P107-0042 | CHEMDIV | 0.66 | -6.27 | -54.87 | -47.48 | 7.24 | -60.34 | 6.03 | -57.72 | 4.78 | -55.18 | 6.01 | 0.03 | -0.46 |
| AO-365/43402979 | SPECS | 0.63 | -6.31 | -56.25 | -58.45 | 5.96 | -57.28 | 9.55 | -48.69 | 12.21 | -54.81 | 9.24 | 0.03 | -0.43 |
| 8020-0488 | CHEMDIV | 0.57 | -6.32 | -50.70 | -48.48 | 10.34 | -55.54 | 7.98 | -59.52 | 5.68 | -54.52 | 8.00 | 0.03 | -0.41 |
| AN-465/42246583 | SPECS | 0.72 | -5.91 | -59.76 | -62.70 | 8.71 | -47.49 | 9.46 | -51.36 | 6.52 | -53.85 | 8.23 | 0.03 | -0.35 |
| AN-465/42887421 | SPECS | 0.60 | -6.17 | -52.85 | -55.34 | 7.28 | -51.41 | 7.12 | -54.04 | 10.07 | -53.60 | 8.16 | 0.03 | -0.33 |
| SB11-0558 | CHEMDIV | 0.84 | -6.48 | -54.81 | -51.08 | 7.02 | -55.93 | 5.20 | -52.83 | 9.39 | -53.28 | 7.20 | 0.03 | -0.31 |
| Z2912331969 | Enamine | 0.50 | -6.50 | -43.57 | -56.50 | 6.50 | -48.25 | 7.86 | -53.21 | 6.03 | -52.65 | 6.80 | 0.03 | -0.26 |
| AN-465/42889905 | SPECS | 0.60 | -6.05 | -59.92 | -56.54 | 7.07 | -57.09 | 6.00 | -44.06 | 6.28 | -52.56 | 6.45 | 0.03 | -0.25 |
| Z2242893563 | Enamine | 0.55 | -6.62 | -54.57 | -48.67 | 6.25 | -54.56 | 7.95 | -53.58 | 6.86 | -52.27 | 7.02 | 0.03 | -0.23 |
| Z1458239348 | Enamine | 0.62 | -6.65 | -49.08 | -50.16 | 11.27 | -59.45 | 8.00 | -45.56 | 9.63 | -51.72 | 9.63 | 0.03 | -0.18 |
| S639-9285 | CHEMDIV | 0.70 | -7.56 | -50.95 | -46.53 | 6.55 | -54.27 | 8.37 | -52.49 | 11.52 | -51.09 | 8.81 | 0.03 | -0.13 |
| AN-465/43012969 | SPECS | 0.58 | -6.38 | -48.48 | -51.21 | 5.61 | -48.70 | 6.23 | -51.68 | 8.51 | -50.53 | 6.79 | 0.03 | -0.09 |
| AJ-030/14523202 | SPECS | 0.68 | -7.47 | -54.78 | -50.83 | 10.66 | -52.68 | 7.89 | -47.94 | 7.19 | -50.49 | 8.58 | 0.03 | -0.08 |
| Z3019210071 | Enamine | 0.55 | -6.64 | -41.59 | -47.56 | 6.12 | -44.89 | 8.75 | -56.82 | 8.01 | -49.76 | 7.63 | 0.03 | -0.02 |
| S551-0276 | CHEMDIV | 0.68 | -6.89 | -53.50 | -43.25 | 5.22 | -51.64 | 6.11 | -53.97 | 8.87 | -49.62 | 6.73 | 0.03 | -0.01 |
| Z2752945600 | Enamine | 0.70 | -6.49 | -45.24 | -52.31 | 6.09 | -44.86 | 10.47 | -50.52 | 7.42 | -49.23 | 7.99 | 0.03 | 0.02 |
| S841-0039 | CHEMDIV | 0.59 | -6.66 | -49.06 | -55.31 | 6.19 | -39.25 | 12.26 | -50.43 | 6.62 | -48.33 | 8.36 | 0.03 | 0.09 |
| S823-0761 | CHEMDIV | 0.76 | -6.83 | -47.34 | -45.24 | 6.51 | -46.53 | 9.30 | -51.19 | 5.90 | -47.65 | 7.23 | 0.03 | 0.15 |
| AN-465/42888023 | SPECS | 0.59 | -5.95 | -58.91 | -59.31 | 8.71 | -42.65 | 6.78 | -40.37 | 7.41 | -47.44 | 7.63 | 0.03 | 0.16 |
| Z2762275562 | Enamine | 0.76 | -6.69 | -49.84 | -39.21 | 7.06 | -55.21 | 7.45 | -47.71 | 6.37 | -47.38 | 6.96 | 0.03 | 0.17 |
| AN-465/42837019 | SPECS | 0.70 | -6.01 | -54.35 | -46.13 | 5.36 | -51.75 | 7.62 | -44.09 | 5.44 | -47.32 | 6.14 | 0.03 | 0.17 |
| AN-465/42888810 | SPECS | 0.67 | -7.01 | -46.76 | -52.15 | 5.70 | -40.89 | 5.96 | -46.44 | 7.53 | -46.49 | 6.40 | 0.03 | 0.24 |
| D340-1053 | CHEMDIV | 0.70 | -6.60 | -54.29 | -45.59 | 6.83 | -46.75 | 10.20 | -44.62 | 5.88 | -45.65 | 7.64 | 0.03 | 0.31 |
| SB91-0954 | CHEMDIV | 0.72 | -6.66 | -47.44 | -53.44 | 6.97 | -31.99 | 10.96 | -51.45 | 7.25 | -45.63 | 8.39 | 0.03 | 0.31 |
| Z1870704769 | Enamine | 0.55 | -6.97 | -49.72 | -46.49 | 8.04 | -35.73 | 4.17 | -54.22 | 10.48 | -45.48 | 7.56 | 0.03 | 0.32 |
| Z2242882612 | Enamine | 0.51 | -7.26 | -46.81 | -40.43 | 9.25 | -43.22 | 8.47 | -51.38 | 7.20 | -45.01 | 8.31 | 0.03 | 0.36 |
| Z1623026591 | Enamine | 0.53 | -6.47 | -44.11 | -47.95 | 8.38 | -36.21 | 8.05 | -49.55 | 6.88 | -44.57 | 7.77 | 0.03 | 0.39 |
| AN-465/43013238 | SPECS | 0.58 | -6.24 | -45.11 | -47.28 | 5.99 | -46.71 | 6.65 | -39.46 | 5.99 | -44.48 | 6.21 | 0.03 | 0.40 |
| Z2242895257 | Enamine | 0.56 | -7.52 | -46.77 | -42.86 | 5.91 | -47.94 | 6.05 | -42.34 | 7.29 | -44.38 | 6.42 | 0.03 | 0.41 |
| AN-465/42888806 | SPECS | 0.71 | -5.92 | -44.99 | -49.68 | 7.36 | -42.08 | 4.81 | -41.18 | 6.64 | -44.31 | 6.27 | 0.03 | 0.42 |
| Z2278180665 | Enamine | 0.61 | -6.73 | -40.55 | -38.59 | 6.58 | -43.13 | 5.59 | -49.91 | 8.86 | -43.88 | 7.01 | 0.03 | 0.45 |
| AB-323/13887094 | SPECS | 0.83 | -6.80 | -44.55 | -43.46 | 6.95 | -42.74 | 4.97 | -43.30 | 5.40 | -43.17 | 5.78 | 0.03 | 0.51 |
| V023-2006 | CHEMDIV | 0.53 | -6.41 | -52.42 | -42.14 | 10.05 | -44.11 | 7.82 | -43.08 | 8.91 | -43.11 | 8.93 | 0.03 | 0.51 |
| AN-465/42887224 | SPECS | 0.72 | -6.17 | -43.86 | -31.92 | 8.79 | -45.41 | 5.95 | -51.64 | 5.88 | -42.99 | 6.88 | 0.03 | 0.52 |
| AE-562/12222311 | SPECS | 0.54 | -7.12 | -43.23 | -41.86 | 8.27 | -47.64 | 5.52 | -36.08 | 6.03 | -41.86 | 6.61 | 0.03 | 0.61 |
| Z2437934768 | Enamine | 0.61 | -7.07 | -38.27 | -41.32 | 4.07 | -38.20 | 3.39 | -45.17 | 4.42 | -41.56 | 3.96 | 0.03 | 0.64 |
| SB91-0822 | CHEMDIV | 0.78 | -6.33 | -56.47 | -37.78 | 9.84 | -40.09 | 7.85 | -44.67 | 7.49 | -40.85 | 8.39 | 0.03 | 0.70 |
| Z2234959965 | Enamine | 0.81 | -6.57 | -47.44 | -39.17 | 10.22 | -43.90 | 5.82 | -37.56 | 5.73 | -40.21 | 7.25 | 0.02 | 0.75 |
| Z2242893701 | Enamine | 0.51 | -6.46 | -44.74 | -42.13 | 6.11 | -36.04 | 5.81 | -41.56 | 5.44 | -39.91 | 5.79 | 0.02 | 0.77 |
| Z800011206 | Enamine | 0.60 | -6.74 | -47.49 | -39.85 | 6.82 | -40.70 | 6.61 | -37.67 | 7.29 | -39.41 | 6.91 | 0.02 | 0.81 |
| AN-465/43384152 | SPECS | 0.67 | -6.37 | -42.33 | -45.04 | 4.84 | -33.03 | 11.31 | -40.02 | 8.30 | -39.36 | 8.15 | 0.02 | 0.82 |
| Z445414324 | Enamine | 0.60 | -7.31 | -40.45 | -40.99 | 8.11 | -36.01 | 5.81 | -40.44 | 6.84 | -39.15 | 6.92 | 0.02 | 0.83 |
| AN-465/42889349 | SPECS | 0.68 | -5.99 | -44.71 | -33.26 | 7.46 | -41.58 | 5.46 | -41.14 | 5.32 | -38.66 | 6.08 | 0.02 | 0.87 |
| Z2418132016 | Enamine | 0.63 | -6.75 | -36.42 | -39.05 | 6.50 | -36.34 | 7.93 | -39.86 | 6.26 | -38.42 | 6.90 | 0.02 | 0.89 |
| SB91-0140 | CHEMDIV | 0.56 | -6.64 | -56.40 | -35.90 | 6.45 | -39.14 | 6.06 | -38.79 | 5.50 | -37.94 | 6.01 | 0.02 | 0.93 |
| Z1491297556 | Enamine | 0.58 | -6.48 | -39.55 | -36.83 | 5.42 | -27.70 | 5.76 | -41.89 | 4.05 | -35.47 | 5.08 | 0.02 | 1.13 |
| SB91-0897 | CHEMDIV | 0.53 | -6.32 | -57.72 | -45.44 | 6.50 | -16.10 | 13.89 | -42.26 | 7.99 | -34.60 | 9.46 | 0.02 | 1.20 |
| SB11-0298 | CHEMDIV | 0.86 | -6.29 | -42.92 | -16.97 | 13.64 | -36.72 | 6.30 | -42.99 | 6.67 | -32.22 | 8.87 | 0.01 | 1.39 |
| Z2796665607 | Enamine | 0.69 | -7.05 | -38.04 | -33.90 | 4.13 | -26.05 | 4.19 | -35.10 | 6.13 | -31.68 | 4.81 | 0.01 | 1.44 |
| 8015-5741 | CHEMDIV | 0.77 | -6.77 | -27.11 | -30.64 | 5.54 | -26.99 | 2.81 | -29.48 | 3.46 | -29.04 | 3.94 | 0.01 | 1.65 |
| 8016-3256 | CHEMDIV | 0.62 | -6.53 | -37.65 | -38.92 | 7.41 | -30.05 | 4.17 | -12.86 | 11.71 | -27.28 | 7.76 | 0.01 | 1.79 |
| CHEMBL3617209 | CHEMBL | 0.83 | -2.97 | -31.33 | -29.34 | 8.13 | -28.53 | 9.54 | -30.14 | 6.08 | -14.07 | 7.92 | 0.00 | 2.86 |

Table S 2: The predicted anticancer activity, docking scores, post-virtual screening workflow average MM/GBSA scores, and the average MM/GBSA scores of the 3 short MD runs performed on the top 25 molecules obtained from each library in the ERCC1 protein’s binding site 2

| **Ligand ID** | **Library** | **Anticancer Activity Prediction** | **Docking Score (kcal/mol)** | **Post-VSW MM/GBSA (kcal/mol)** | **MM/GBSA Run 1 (kcal/mol)** | **Standard Deviation Run 1** | **MM/GBSA Run 2 (kcal/mol)** | **Standard Deviation Run 2** | **MM/GBSA Run 3 (kcal/mol)** | **Standard Deviation Run 3** | **MMGBSA Average Score (kcal/mol)** | **Average Standard Deviation** | **Normal Distribution** | **Z-score** |
| --- | --- | --- | --- | --- | --- | --- | --- | --- | --- | --- | --- | --- | --- | --- |
| Y501-9249 | CHEMDIV | 0.74 | -5.24 | -42.34 | -60.40 | 6.61 | -58.41 | 6.23 | -65.35 | 9.17 | -61.38 | 7.34 | 0.00 | -2.36 |
| Z1450326974 | Enamine | 0.69 | -5.56 | -40.27 | -56.85 | 9.11 | -55.38 | 6.60 | -58.84 | 7.30 | -57.03 | 7.67 | 0.01 | -1.77 |
| K219-1359 | CHEMDIV | 0.51 | -5.40 | -46.36 | -56.87 | 5.08 | -54.02 | 9.26 | -58.59 | 4.86 | -56.49 | 6.40 | 0.01 | -1.70 |
| Z18519308 | Enamine | 0.84 | -5.69 | -40.42 | -52.37 | 5.97 | -58.53 | 5.15 | -56.20 | 5.01 | -55.70 | 5.38 | 0.02 | -1.59 |
| Z1823992925 | Enamine | 0.78 | -6.34 | -37.78 | -55.19 | 7.54 | -43.73 | 7.59 | -65.80 | 8.17 | -54.91 | 7.77 | 0.02 | -1.48 |
| 8017-4372 | CHEMDIV | 0.59 | -5.44 | -41.74 | -53.71 | 6.96 | -61.57 | 9.13 | -49.26 | 7.11 | -54.85 | 7.73 | 0.02 | -1.48 |
| G754-0040 | CHEMDIV | 0.63 | -6.27 | -46.38 | -54.76 | 10.95 | -52.43 | 10.93 | -55.27 | 5.66 | -54.15 | 9.18 | 0.02 | -1.38 |
| Z1128091108 | Enamine | 0.50 | -5.69 | -48.73 | -51.23 | 8.79 | -50.44 | 5.76 | -59.57 | 5.74 | -53.75 | 6.76 | 0.02 | -1.33 |
| Z446559698 | Enamine | 0.69 | -6.56 | -48.02 | -54.25 | 6.37 | -54.31 | 5.84 | -51.87 | 7.22 | -53.48 | 6.48 | 0.02 | -1.29 |
| AK-968/11181105 | SPECS | 0.52 | -5.65 | -45.18 | -56.03 | 5.71 | -38.15 | 8.25 | -65.40 | 9.93 | -53.19 | 7.96 | 0.02 | -1.25 |
| AR-422/41674182 | SPECS | 0.87 | -5.47 | -45.45 | -58.21 | 6.31 | -51.38 | 5.32 | -46.59 | 4.92 | -52.06 | 5.51 | 0.03 | -1.10 |
| AQ-390/41116338 | SPECS | 0.64 | -5.59 | -42.92 | -53.72 | 7.93 | -57.64 | 11.63 | -43.13 | 5.98 | -51.49 | 8.52 | 0.03 | -1.03 |
| AI-067/31572061 | SPECS | 0.83 | -5.63 | -44.77 | -63.94 | 10.55 | -41.65 | 5.59 | -48.00 | 4.18 | -51.20 | 6.77 | 0.03 | -0.99 |
| Z968561674 | Enamine | 0.51 | -5.79 | -41.01 | -44.91 | 5.55 | -64.03 | 12.04 | -44.14 | 9.81 | -51.03 | 9.13 | 0.03 | -0.96 |
| Z1521943091 | Enamine | 0.62 | -5.61 | -36.86 | -52.40 | 10.09 | -39.99 | 6.28 | -58.12 | 9.32 | -50.17 | 8.56 | 0.04 | -0.85 |
| 8005-8451 | CHEMDIV | 0.50 | -6.38 | -41.23 | -56.86 | 6.95 | -44.13 | 5.49 | -47.32 | 7.16 | -49.44 | 6.53 | 0.04 | -0.75 |
| AO-081/40878566 | SPECS | 0.65 | -5.97 | -35.80 | -42.67 | 7.26 | -49.82 | 5.85 | -54.63 | 10.16 | -49.04 | 7.76 | 0.04 | -0.69 |
| AE-848/01519002 | SPECS | 0.66 | -6.23 | -46.78 | -51.52 | 4.36 | -46.02 | 6.12 | -48.97 | 5.95 | -48.84 | 5.48 | 0.04 | -0.67 |
| AI-204/31696051 | SPECS | 0.80 | -5.53 | -35.05 | -62.86 | 10.13 | -41.53 | 4.75 | -41.76 | 9.20 | -48.72 | 8.03 | 0.04 | -0.65 |
| Z45679838 | Enamine | 0.54 | -5.88 | -49.40 | -56.61 | 7.80 | -45.89 | 5.74 | -43.48 | 7.07 | -48.66 | 6.87 | 0.04 | -0.64 |
| AP-263/43491146 | SPECS | 0.61 | -5.88 | -41.09 | -48.84 | 6.98 | -49.74 | 6.76 | -45.82 | 4.45 | -48.13 | 6.06 | 0.05 | -0.57 |
| G751-2008 | CHEMDIV | 0.60 | -5.42 | -35.24 | -48.82 | 7.40 | -51.67 | 6.96 | -43.08 | 7.53 | -47.86 | 7.30 | 0.05 | -0.54 |
| P163-0706 | CHEMDIV | 0.52 | -5.46 | -36.14 | -48.06 | 5.32 | -41.70 | 7.85 | -51.49 | 5.92 | -47.08 | 6.36 | 0.05 | -0.43 |
| AP-263/43502846 | SPECS | 0.52 | -5.51 | -46.12 | -44.61 | 5.22 | -45.04 | 5.11 | -49.33 | 5.83 | -46.33 | 5.38 | 0.05 | -0.33 |
| AP-263/43418363 | SPECS | 0.82 | -5.85 | -40.42 | -41.18 | 7.15 | -50.33 | 6.23 | -47.31 | 4.87 | -46.27 | 6.08 | 0.05 | -0.32 |
| AP-263/43418352 | SPECS | 0.69 | -6.34 | -40.09 | -43.60 | 7.97 | -48.06 | 4.19 | -46.34 | 5.92 | -46.00 | 6.03 | 0.05 | -0.29 |
| Z45658670 | Enamine | 0.82 | -5.85 | -42.08 | -41.94 | 4.87 | -53.92 | 6.30 | -42.03 | 4.34 | -45.96 | 5.17 | 0.05 | -0.28 |
| Z365118504 | Enamine | 0.59 | -6.25 | -30.80 | -41.06 | 4.95 | -48.48 | 9.24 | -48.03 | 6.30 | -45.86 | 6.83 | 0.05 | -0.27 |
| Z45554450 | Enamine | 0.56 | -5.52 | -42.52 | -46.95 | 3.84 | -50.35 | 11.88 | -40.05 | 5.45 | -45.78 | 7.06 | 0.05 | -0.26 |
| AP-263/43491190 | SPECS | 0.64 | -5.64 | -43.97 | -41.20 | 7.77 | -54.84 | 6.89 | -41.07 | 8.53 | -45.71 | 7.73 | 0.05 | -0.25 |
| AE-848/07197030 | SPECS | 0.82 | -5.53 | -44.25 | -45.87 | 4.09 | -42.31 | 6.82 | -48.07 | 6.56 | -45.42 | 5.82 | 0.05 | -0.21 |
| Z2064307715 | Enamine | 0.58 | -6.08 | -37.29 | -34.81 | 6.26 | -49.96 | 7.15 | -50.96 | 7.44 | -45.25 | 6.95 | 0.05 | -0.18 |
| Z970066398 | Enamine | 0.65 | -5.65 | -47.98 | -53.14 | 5.48 | -35.97 | 6.75 | -46.39 | 4.10 | -45.17 | 5.44 | 0.05 | -0.17 |
| Z1467481257 | Enamine | 0.60 | -5.54 | -34.99 | -41.26 | 7.21 | -46.22 | 8.92 | -47.57 | 6.48 | -45.02 | 7.54 | 0.05 | -0.15 |
| Y020-8110 | CHEMDIV | 0.70 | -5.56 | -30.12 | -44.17 | 5.86 | -45.91 | 8.25 | -44.73 | 5.95 | -44.94 | 6.69 | 0.05 | -0.14 |
| AP-263/43491147 | SPECS | 0.59 | -6.27 | -38.58 | -41.80 | 6.81 | -50.47 | 8.94 | -42.22 | 7.33 | -44.83 | 7.69 | 0.05 | -0.13 |
| AK-918/10657028 | SPECS | 0.53 | -5.50 | -38.22 | -56.78 | 7.04 | -37.60 | 10.96 | -39.66 | 4.68 | -44.68 | 7.56 | 0.05 | -0.11 |
| S965-0090 | CHEMDIV | 0.83 | -5.73 | -33.17 | -45.31 | 3.90 | -39.87 | 5.53 | -48.76 | 8.19 | -44.65 | 5.87 | 0.05 | -0.10 |
| 4261-0082 | CHEMDIV | 0.61 | -5.10 | -33.38 | -47.58 | 5.34 | -51.93 | 8.14 | -34.32 | 3.51 | -44.61 | 5.66 | 0.05 | -0.10 |
| AQ-390/43238221 | SPECS | 0.82 | -5.43 | -42.89 | -44.49 | 3.92 | -46.99 | 10.06 | -42.18 | 5.21 | -44.55 | 6.39 | 0.05 | -0.09 |
| 8018-7999 | CHEMDIV | 0.60 | -5.55 | -39.56 | -45.52 | 5.59 | -40.30 | 5.92 | -46.87 | 5.39 | -44.23 | 5.63 | 0.05 | -0.05 |
| Z45636549 | Enamine | 0.58 | -5.61 | -37.58 | -45.01 | 5.64 | -38.35 | 5.73 | -48.83 | 5.64 | -44.06 | 5.67 | 0.05 | -0.02 |
| AG-205/37130076 | SPECS | 0.82 | -5.71 | -39.34 | -53.57 | 6.55 | -42.81 | 5.58 | -35.31 | 8.51 | -43.90 | 6.88 | 0.05 | 0.00 |
| 8018-0236 | CHEMDIV | 0.66 | -5.12 | -33.40 | -43.44 | 5.15 | -38.47 | 4.37 | -49.43 | 5.92 | -43.78 | 5.15 | 0.05 | 0.01 |
| 8013-5568 | CHEMDIV | 0.71 | -5.24 | -22.63 | -39.04 | 5.94 | -46.75 | 4.58 | -44.02 | 5.64 | -43.27 | 5.38 | 0.05 | 0.08 |
| Y502-4533 | CHEMDIV | 0.81 | -5.85 | -45.35 | -52.70 | 6.61 | -40.10 | 6.47 | -36.31 | 8.47 | -43.03 | 7.18 | 0.05 | 0.11 |
| AN-465/41853741 | SPECS | 0.55 | -6.02 | -29.56 | -48.46 | 8.16 | -36.73 | 7.52 | -43.44 | 4.02 | -42.88 | 6.56 | 0.05 | 0.13 |
| Z601-6344 | CHEMDIV | 0.74 | -5.25 | -47.32 | -33.45 | 6.11 | -54.07 | 6.76 | -39.57 | 8.96 | -42.36 | 7.27 | 0.05 | 0.20 |
| AE-848/00888035 | SPECS | 0.57 | -5.85 | -40.98 | -40.31 | 6.91 | -45.34 | 6.45 | -39.87 | 5.98 | -41.84 | 6.45 | 0.05 | 0.27 |
| Z1716746924 | Enamine | 0.62 | -6.68 | -41.25 | -39.47 | 8.93 | -40.18 | 5.52 | -44.84 | 5.61 | -41.50 | 6.68 | 0.05 | 0.32 |
| Y031-8657 | CHEMDIV | 0.64 | -5.30 | -33.80 | -30.43 | 5.57 | -39.52 | 8.09 | -54.30 | 7.97 | -41.42 | 7.21 | 0.05 | 0.33 |
| Z45510154 | Enamine | 0.87 | -5.80 | -36.00 | -37.93 | 5.68 | -43.76 | 4.50 | -42.10 | 4.33 | -41.26 | 4.84 | 0.05 | 0.35 |
| 8016-4307 | CHEMDIV | 0.62 | -5.21 | -28.99 | -37.30 | 7.90 | -40.59 | 7.66 | -45.87 | 8.73 | -41.25 | 8.10 | 0.05 | 0.35 |
| AN-652/34817040 | SPECS | 0.82 | -5.52 | -43.53 | -44.87 | 7.73 | -35.76 | 6.60 | -42.87 | 5.55 | -41.17 | 6.63 | 0.05 | 0.36 |
| Z2960705137 | Enamine | 0.55 | -5.54 | -39.46 | -40.61 | 8.91 | -38.34 | 8.92 | -40.80 | 5.71 | -39.92 | 7.85 | 0.05 | 0.53 |
| Y503-4553 | CHEMDIV | 0.82 | -5.42 | -38.00 | -32.48 | 5.70 | -42.06 | 4.72 | -43.88 | 8.56 | -39.47 | 6.33 | 0.05 | 0.59 |
| C800-0909 | CHEMDIV | 0.66 | -5.17 | -37.51 | -38.41 | 4.86 | -37.63 | 5.69 | -40.95 | 5.85 | -38.99 | 5.47 | 0.04 | 0.66 |
| AP-263/43418989 | SPECS | 0.69 | -5.45 | -43.55 | -46.66 | 5.17 | -34.34 | 6.94 | -35.66 | 5.93 | -38.89 | 6.01 | 0.04 | 0.67 |
| AP-263/43371237 | SPECS | 0.87 | -5.82 | -40.21 | -33.87 | 6.81 | -39.49 | 5.76 | -43.02 | 4.85 | -38.79 | 5.81 | 0.04 | 0.68 |
| S059-0273 | CHEMDIV | 0.85 | -5.46 | -36.50 | -34.60 | 6.37 | -34.45 | 7.09 | -44.02 | 7.83 | -37.69 | 7.10 | 0.04 | 0.83 |
| AO-854/43462383 | SPECS | 0.87 | -5.43 | -29.92 | -33.83 | 4.84 | -46.69 | 6.46 | -32.25 | 4.96 | -37.59 | 5.42 | 0.04 | 0.85 |
| CM4439-0048 | CHEMDIV | 0.76 | -5.59 | -34.28 | -42.11 | 7.16 | -39.34 | 5.18 | -30.76 | 4.44 | -37.40 | 5.59 | 0.04 | 0.87 |
| Z1459347160 | Enamine | 0.55 | -6.09 | -29.60 | -40.71 | 7.38 | -35.45 | 5.50 | -35.58 | 7.03 | -37.25 | 6.63 | 0.04 | 0.89 |
| Z1536831640 | Enamine | 0.59 | -6.83 | -38.74 | -29.91 | 5.87 | -40.79 | 5.92 | -40.57 | 6.82 | -37.09 | 6.20 | 0.04 | 0.91 |
| 8017-9618 | CHEMDIV | 0.88 | -5.17 | -37.79 | -31.32 | 7.00 | -36.80 | 8.39 | -42.03 | 4.46 | -36.72 | 6.62 | 0.03 | 0.96 |
| Z2975550894 | Enamine | 0.84 | -5.89 | -36.88 | -39.56 | 3.75 | -40.56 | 4.49 | -29.06 | 5.55 | -36.39 | 4.60 | 0.03 | 1.01 |
| AO-854/43450495 | SPECS | 0.56 | -5.66 | -42.30 | -50.17 | 7.55 | -25.57 | 11.30 | -32.50 | 8.46 | -36.08 | 9.10 | 0.03 | 1.05 |
| Z45636550 | Enamine | 0.52 | -6.49 | -26.17 | -48.28 | 8.04 | -34.52 | 6.06 | -24.08 | 4.70 | -35.63 | 6.26 | 0.03 | 1.11 |
| Z1931407411 | Enamine | 0.59 | -5.66 | -40.22 | -41.00 | 8.42 | -32.00 | 13.56 | -31.65 | 6.66 | -34.88 | 9.55 | 0.03 | 1.21 |
| Z2497328193 | Enamine | 0.64 | -5.88 | -37.94 | -28.48 | 5.54 | -41.94 | 5.62 | -30.56 | 4.70 | -33.66 | 5.29 | 0.02 | 1.37 |
| 8015-5176 | CHEMDIV | 0.75 | -5.33 | -27.36 | -29.95 | 3.99 | -40.27 | 4.40 | -29.77 | 10.06 | -33.33 | 6.15 | 0.02 | 1.42 |
| AP-263/43479523 | SPECS | 0.65 | -5.44 | -39.85 | -13.88 | 14.90 | -49.51 | 3.75 | -30.79 | 6.27 | -31.39 | 8.31 | 0.01 | 1.68 |
| P048-1722 | CHEMDIV | 0.54 | -5.12 | -30.93 | -25.54 | 8.78 | -34.89 | 5.56 | -32.78 | 5.72 | -31.07 | 6.69 | 0.01 | 1.72 |
| 8011-8369 | CHEMDIV | 0.82 | -5.85 | -32.74 | -26.13 | 4.21 | -29.94 | 5.81 | -31.81 | 4.62 | -29.30 | 4.88 | 0.01 | 1.96 |
| Z2242930093 | Enamine | 0.81 | -5.86 | -37.50 | -21.27 | 9.05 | -25.36 | 6.43 | -32.64 | 6.12 | -26.43 | 7.20 | 0.00 | 2.35 |
| CHEMBL3617209 | CHEMBL | 0.69 | -4.72 | -29.43 | -36.62 | 5.19 | -36.64 | 7.15 | -44.04 | 9.53 | -20.99 | 7.29 | 0.00 | 3.08 |

Table S 3: The predicted anticancer activity, docking scores, post-virtual screening workflow average MM/GBSA scores, and the average MM/GBSA scores of the 3 short MD runs performed on the top 25 molecules obtained from each library in the ERCC1 protein’s binding site 3

| **Ligand ID** | **Library** | **Anticancer Activity Prediction** | **Docking Score (kcal/mol)** | **Post-VSW MM/GBSA (kcal/mol)** | **MM/GBSA Run 1 (kcal/mol)** | **Standard Deviation Run 1** | **MM/GBSA Run 2 (kcal/mol)** | **Standard Deviation Run 2** | **MM/GBSA Run 3 (kcal/mol)** | **Standard Deviation Run 3** | **MMGBSA Average Score (kcal/mol)** | **Average Standard Deviation** | **Normal Distribution** | **Z-score** |
| --- | --- | --- | --- | --- | --- | --- | --- | --- | --- | --- | --- | --- | --- | --- |
| G605-0598 | CHEMDIV | 0.71 | -5.71 | -62.81 | -68.18 | 8.9832 | -48.96 | 4.2877 | -63.94 | 5.1495 | -60.36 | 6.14 | 0.01 | -2.07 |
| D264-0862 | CHEMDIV | 0.58 | -5.85 | -58.31 | -62.01 | 4.3048 | -61.25 | 4.664 | -57.24 | 4.4707 | -60.17 | 4.48 | 0.01 | -2.05 |
| V019-9483 | CHEMDIV | 0.64 | -5.92 | -52.11 | -57.35 | 12.7024 | -58.08 | 6.463 | -57.71 | 8.1237 | -57.71 | 9.10 | 0.01 | -1.74 |
| Z2441063089 | Enamine | 0.78 | -5.80 | -51.99 | -57.89 | 8.9818 | -49.47 | 9.1169 | -62.90 | 5.8225 | -56.76 | 7.97 | 0.01 | -1.63 |
| Z1818284484 | Enamine | 0.64 | -5.90 | -53.00 | -45.39 | 7.4976 | -67.19 | 5.4995 | -47.69 | 9.5117 | -53.42 | 7.50 | 0.02 | -1.22 |
| AN-465/43384083 | SPECS | 0.59 | -5.35 | -46.68 | -52.94 | 6.0644 | -45.12 | 6.7588 | -61.61 | 13.0908 | -53.22 | 8.64 | 0.02 | -1.19 |
| Z409970406 | Enamine | 0.56 | -5.90 | -54.80 | -56.19 | 4.5025 | -43.06 | 5.9343 | -60.22 | 4.842 | -53.16 | 5.09 | 0.02 | -1.18 |
| L437-0017 | CHEMDIV | 0.77 | -5.54 | -55.67 | -41.53 | 4.968 | -56.63 | 3.9756 | -61.09 | 6.2185 | -53.09 | 5.05 | 0.02 | -1.18 |
| S049-0360 | CHEMDIV | 0.66 | -5.69 | -44.10 | -57.57 | 11.5565 | -39.27 | 6.6281 | -57.03 | 9.1436 | -51.29 | 9.11 | 0.03 | -0.96 |
| Z338264330 | Enamine | 0.70 | -5.80 | -43.80 | -45.09 | 5.2201 | -49.70 | 4.8674 | -58.41 | 7.5941 | -51.07 | 5.89 | 0.03 | -0.93 |
| V019-7112 | CHEMDIV | 0.63 | -5.93 | -53.98 | -45.70 | 7.5031 | -55.06 | 7.8042 | -52.19 | 7.7068 | -50.98 | 7.67 | 0.03 | -0.92 |
| Y021-2083 | CHEMDIV | 0.64 | -5.61 | -47.23 | -52.30 | 8.5078 | -52.82 | 5.8112 | -46.51 | 15.6634 | -50.55 | 9.99 | 0.03 | -0.86 |
| AN-465/43384141 | SPECS | 0.71 | -5.41 | -49.34 | -37.89 | 5.6628 | -57.98 | 6.5219 | -55.11 | 6.6374 | -50.33 | 6.27 | 0.03 | -0.84 |
| J107-0623 | CHEMDIV | 0.82 | -5.52 | -43.06 | -35.43 | 7.2651 | -51.44 | 9.1639 | -63.92 | 6.402 | -50.26 | 7.61 | 0.03 | -0.83 |
| AN-465/42837105 | SPECS | 0.66 | -5.48 | -49.44 | -57.16 | 8.5278 | -43.45 | 7.2165 | -48.51 | 7.1079 | -49.71 | 7.62 | 0.04 | -0.76 |
| AM-807/43276749 | SPECS | 0.78 | -5.50 | -45.39 | -56.94 | 7.6258 | -41.86 | 9.4728 | -49.99 | 5.2246 | -49.59 | 7.44 | 0.04 | -0.75 |
| AN-465/43384157 | SPECS | 0.58 | -5.80 | -48.25 | -56.81 | 6.9119 | -46.40 | 11.2671 | -44.23 | 6.0627 | -49.14 | 8.08 | 0.04 | -0.69 |
| Z747044084 | Enamine | 0.58 | -5.66 | -46.17 | -45.69 | 5.8374 | -47.27 | 6.444 | -53.81 | 7.0597 | -48.92 | 6.45 | 0.04 | -0.66 |
| AN-465/43384078 | SPECS | 0.55 | -5.92 | -41.72 | -44.77 | 9.0103 | -53.38 | 9.9656 | -48.46 | 5.9242 | -48.87 | 8.30 | 0.04 | -0.66 |
| 8015-8045 | CHEMDIV | 0.55 | -5.50 | -39.65 | -39.69 | 13.759 | -49.96 | 6.2726 | -56.70 | 5.8483 | -48.78 | 8.63 | 0.04 | -0.65 |
| V012-9015 | CHEMDIV | 0.65 | -6.15 | -54.45 | -47.16 | 6.2383 | -59.83 | 7.2992 | -38.42 | 6.6523 | -48.47 | 6.73 | 0.04 | -0.61 |
| Y600-0025 | CHEMDIV | 0.77 | -5.70 | -51.22 | -46.93 | 4.5513 | -52.32 | 3.3932 | -46.01 | 4.287 | -48.42 | 4.08 | 0.04 | -0.60 |
| Z826239562 | Enamine | 0.52 | -6.09 | -58.63 | -43.08 | 10.317 | -54.22 | 5.2375 | -47.96 | 8.2624 | -48.42 | 7.94 | 0.04 | -0.60 |
| Z2379493624 | Enamine | 0.80 | -5.75 | -50.14 | -53.14 | 8.8878 | -42.81 | 6.9376 | -48.21 | 9.4239 | -48.06 | 8.42 | 0.04 | -0.56 |
| AN-465/43384081 | SPECS | 0.56 | -5.59 | -45.00 | -47.26 | 5.3955 | -47.41 | 5.5293 | -48.95 | 7.677 | -47.87 | 6.20 | 0.04 | -0.54 |
| Z1818285179 | Enamine | 0.77 | -5.74 | -43.22 | -46.97 | 7.4981 | -49.01 | 5.8388 | -47.17 | 8.5206 | -47.71 | 7.29 | 0.04 | -0.52 |
| AN-465/43384051 | SPECS | 0.64 | -5.48 | -46.72 | -57.47 | 11.0844 | -43.07 | 4.7391 | -41.35 | 4.0715 | -47.30 | 6.63 | 0.04 | -0.47 |
| Z2464461011 | Enamine | 0.67 | -5.95 | -46.91 | -47.44 | 6.5079 | -50.36 | 8.6534 | -44.07 | 4.8262 | -47.29 | 6.66 | 0.04 | -0.46 |
| AG-690/11062031 | SPECS | 0.74 | -5.49 | -51.23 | -49.43 | 5.1663 | -42.98 | 6.9809 | -48.94 | 6.7603 | -47.12 | 6.30 | 0.04 | -0.44 |
| AK-968/13408045 | SPECS | 0.77 | -5.54 | -51.23 | -51.71 | 5.2243 | -47.13 | 4.7424 | -42.25 | 7.1787 | -47.03 | 5.72 | 0.04 | -0.43 |
| Z2712023074 | Enamine | 0.63 | -5.66 | -48.04 | -41.84 | 6.2648 | -46.52 | 6.2996 | -51.13 | 6.1383 | -46.50 | 6.23 | 0.05 | -0.37 |
| SB70-0816 | CHEMDIV | 0.81 | -5.60 | -54.23 | -42.31 | 7.9295 | -44.51 | 6.9688 | -50.44 | 6.8389 | -45.75 | 7.25 | 0.05 | -0.28 |
| Z2355059376 | Enamine | 0.77 | -5.70 | -43.58 | -41.62 | 6.8566 | -47.39 | 5.3204 | -47.74 | 7.726 | -45.58 | 6.63 | 0.05 | -0.25 |
| E567-0013 | CHEMDIV | 0.55 | -5.53 | -45.73 | -52.27 | 4.7821 | -48.13 | 5.9951 | -36.30 | 7.2323 | -45.57 | 6.00 | 0.05 | -0.25 |
| AO-022/43512953 | SPECS | 0.65 | -5.67 | -45.04 | -39.82 | 7.0055 | -50.29 | 8.591 | -46.44 | 7.9477 | -45.52 | 7.85 | 0.05 | -0.25 |
| S596-1185 | CHEMDIV | 0.50 | -5.94 | -47.99 | -36.97 | 6.6359 | -47.80 | 7.8602 | -48.59 | 9.6809 | -44.45 | 8.06 | 0.05 | -0.12 |
| AN-465/42889973 | SPECS | 0.74 | -5.40 | -55.56 | -44.35 | 7.1695 | -41.09 | 9.6366 | -47.27 | 7.7189 | -44.24 | 8.18 | 0.05 | -0.09 |
| Z2975071803 | Enamine | 0.73 | -6.08 | -47.19 | -50.15 | 5.9809 | -51.67 | 5.2096 | -30.88 | 10.5735 | -44.23 | 7.25 | 0.05 | -0.09 |
| C908-0508 | CHEMDIV | 0.67 | -5.91 | -45.07 | -43.57 | 6.3544 | -45.38 | 6.0449 | -43.44 | 6.8743 | -44.13 | 6.42 | 0.05 | -0.08 |
| AN-465/43384050 | SPECS | 0.57 | -5.53 | -45.89 | -45.89 | 5.084 | -38.07 | 6.5371 | -48.39 | 7.5422 | -44.12 | 6.39 | 0.05 | -0.07 |
| AN-465/43384156 | SPECS | 0.57 | -5.39 | -52.74 | -46.41 | 5.3217 | -41.70 | 6.1395 | -41.85 | 5.4471 | -43.32 | 5.64 | 0.05 | 0.02 |
| AN-465/43384164 | SPECS | 0.73 | -5.60 | -49.23 | -53.38 | 7.3265 | -37.85 | 10.0864 | -38.62 | 7.2529 | -43.29 | 8.22 | 0.05 | 0.03 |
| AN-465/43384142 | SPECS | 0.71 | -5.47 | -48.94 | -40.32 | 3.9869 | -46.78 | 10.0869 | -41.60 | 6.5421 | -42.90 | 6.87 | 0.05 | 0.07 |
| 8012-6537 | CHEMDIV | 0.75 | -5.56 | -44.50 | -47.04 | 7.586 | -34.87 | 7.2197 | -46.24 | 6.4597 | -42.72 | 7.09 | 0.05 | 0.10 |
| SC25-0763 | CHEMDIV | 0.50 | -6.03 | -47.26 | -37.77 | 7.316 | -41.22 | 5.9889 | -48.33 | 6.7212 | -42.44 | 6.68 | 0.05 | 0.13 |
| Z2464646124 | Enamine | 0.71 | -5.86 | -48.02 | -35.68 | 6.0624 | -46.48 | 8.1268 | -45.11 | 7.701 | -42.42 | 7.30 | 0.05 | 0.13 |
| AN-465/43411276 | SPECS | 0.73 | -5.48 | -51.52 | -37.28 | 6.626 | -46.18 | 6.6953 | -43.65 | 6.5854 | -42.37 | 6.64 | 0.05 | 0.14 |
| AN-465/43384162 | SPECS | 0.73 | -5.36 | -49.24 | -50.30 | 9.5117 | -39.14 | 4.8065 | -35.72 | 3.7036 | -41.72 | 6.01 | 0.05 | 0.22 |
| Z167650748 | Enamine | 0.52 | -5.71 | -44.97 | -37.62 | 5.5098 | -45.62 | 5.9404 | -41.44 | 4.9721 | -41.56 | 5.47 | 0.05 | 0.24 |
| Z2440089400 | Enamine | 0.74 | -5.90 | -48.50 | -39.66 | 5.1866 | -32.23 | 9.1386 | -52.29 | 10.6316 | -41.39 | 8.32 | 0.05 | 0.26 |
| 5655-0305 | CHEMDIV | 0.69 | -5.74 | -52.65 | -56.48 | 8.4437 | -40.77 | 8.0929 | -26.76 | 14.9249 | -41.34 | 10.49 | 0.05 | 0.27 |
| Z2465025707 | Enamine | 0.68 | -6.11 | -40.48 | -44.48 | 5.8567 | -46.95 | 6.7967 | -31.86 | 4.634 | -41.10 | 5.76 | 0.05 | 0.30 |
| Z1552203481 | Enamine | 0.65 | -5.78 | -49.96 | -39.56 | 4.1768 | -43.18 | 5.4466 | -40.42 | 3.7839 | -41.06 | 4.47 | 0.05 | 0.30 |
| D264-0858 | CHEMDIV | 0.64 | -5.87 | -54.50 | -38.37 | 4.9129 | -42.98 | 5 | -41.62 | 6.4344 | -40.99 | 5.45 | 0.05 | 0.31 |
| V012-8627 | CHEMDIV | 0.61 | -5.57 | -44.94 | -42.45 | 8.3028 | -37.45 | 5.7805 | -42.80 | 6.0666 | -40.90 | 6.72 | 0.05 | 0.32 |
| SC85-0539 | CHEMDIV | 0.57 | -5.58 | -49.21 | -30.40 | 12.145 | -47.24 | 4.6685 | -44.90 | 5.5307 | -40.85 | 7.45 | 0.05 | 0.33 |
| Z2364064474 | Enamine | 0.56 | -6.18 | -40.68 | -28.81 | 6.2708 | -46.66 | 10.255 | -45.59 | 7.7145 | -40.35 | 8.08 | 0.05 | 0.39 |
| Z2362433806 | Enamine | 0.61 | -6.25 | -46.32 | -38.23 | 6.646 | -41.46 | 7.1653 | -41.35 | 5.9867 | -40.35 | 6.60 | 0.05 | 0.39 |
| E852-2310 | CHEMDIV | 0.77 | -5.63 | -50.67 | -38.04 | 6.9756 | -40.52 | 7.9764 | -42.45 | 4.271 | -40.33 | 6.41 | 0.05 | 0.39 |
| AP-853/42161332 | SPECS | 0.68 | -5.78 | -48.23 | -36.94 | 7.9073 | -37.43 | 3.6173 | -46.24 | 4.9975 | -40.20 | 5.51 | 0.05 | 0.41 |
| Z2447081864 | Enamine | 0.57 | -5.76 | -48.71 | -37.34 | 5.6611 | -38.45 | 5.84 | -44.33 | 5.8557 | -40.04 | 5.79 | 0.04 | 0.43 |
| Z2394518759 | Enamine | 0.64 | -5.73 | -51.64 | -42.90 | 7.6741 | -37.62 | 5.0536 | -36.25 | 6.863 | -38.92 | 6.53 | 0.04 | 0.56 |
| AN-465/43384082 | SPECS | 0.60 | -5.43 | -48.58 | -33.42 | 12.1102 | -41.91 | 5.024 | -37.22 | 7.9012 | -37.52 | 8.35 | 0.04 | 0.74 |
| AJ-030/14523202 | SPECS | 0.68 | -6.38 | -47.20 | -36.27 | 6.155 | -35.26 | 6.7533 | -40.72 | 7.2301 | -37.41 | 6.71 | 0.04 | 0.75 |
| AN-465/43384049 | SPECS | 0.61 | -5.37 | -48.64 | -39.17 | 5.8964 | -29.08 | 12.3218 | -43.95 | 5.3996 | -37.40 | 7.87 | 0.04 | 0.75 |
| AN-465/43411466 | SPECS | 0.57 | -5.41 | -47.58 | -30.14 | 4.1046 | -37.24 | 5.6139 | -43.99 | 5.0847 | -37.12 | 4.93 | 0.04 | 0.78 |
| AP-124/43382853 | SPECS | 0.60 | -5.56 | -39.68 | -39.86 | 5.9216 | -33.09 | 5.2999 | -38.02 | 5.7607 | -36.99 | 5.66 | 0.04 | 0.80 |
| Z927436652 | Enamine | 0.64 | -5.70 | -48.53 | -35.20 | 5.2473 | -35.47 | 7.9816 | -35.06 | 5.5982 | -35.24 | 6.28 | 0.03 | 1.02 |
| Z819969812 | Enamine | 0.53 | -5.77 | -42.84 | -32.42 | 7.423 | -34.41 | 5.3765 | -38.23 | 7.4196 | -35.02 | 6.74 | 0.03 | 1.04 |
| N121-0953 | CHEMDIV | 0.54 | -5.54 | -56.53 | -40.54 | 9.2131 | -7.97 | 14.428 | -55.17 | 8.7598 | -34.56 | 10.80 | 0.03 | 1.10 |
| D124-0178 | CHEMDIV | 0.51 | -5.50 | -41.16 | -39.37 | 5.5889 | -23.10 | 16.1454 | -36.33 | 4.9742 | -32.93 | 8.90 | 0.02 | 1.30 |
| AE-562/12222311 | SPECS | 0.54 | -5.36 | -40.03 | -31.65 | 4.5259 | -31.58 | 6.3623 | -35.02 | 8.3014 | -32.75 | 6.40 | 0.02 | 1.32 |
| D351-0071 | CHEMDIV | 0.64 | -5.85 | -41.40 | -28.13 | 4.9899 | -35.27 | 7.5162 | -33.27 | 5.6252 | -32.22 | 6.04 | 0.02 | 1.39 |
| Z927307644 | Enamine | 0.70 | -6.76 | -44.67 | -29.10 | 6.2076 | -35.39 | 5.5972 | -29.34 | 5.5087 | -31.28 | 5.77 | 0.02 | 1.50 |
| Z2772942724 | Enamine | 0.58 | -5.87 | -46.06 | -15.32 | 14.9677 | -38.47 | 9.3788 | -38.27 | 7.6546 | -30.69 | 10.67 | 0.01 | 1.57 |
| CHEMBL3617209 | CHEMBL | 0.83 | -2.97 | -31.33 | -29.34 | 5.5872 | -28.53 | 2.8882 | -30.14 | 3.6956 | -15.91 | 4.06 | 0.00 | 3.39 |

Table S 4: The predicted anticancer activity, docking scores, post-virtual screening workflow average MM/GBSA scores, and the average MM/GBSA scores of the 3 short MD runs performed on the top 25 molecules obtained from each library in the ERCC1/XPF protein complex’s binding site 1

| **Ligand ID** | **Library** | **Anticancer Activity Prediction** | **Docking Score (kcal/mol)** | **Post-VSW MM/GBSA (kcal/mol)** | **MM/GBSA Run 1 (kcal/mol)** | **Standard Deviation Run 1** | **MM/GBSA Run 2 (kcal/mol)** | **Standard Deviation Run 2** | **MM/GBSA Run 3 (kcal/mol)** | **Standard Deviation Run 3** | **MMGBSA Average (kcal/mol)** | **Standard Deviation Average** | **Normal Distribution** | **Z-score** |
| --- | --- | --- | --- | --- | --- | --- | --- | --- | --- | --- | --- | --- | --- | --- |
| ZC46-0199 | CHEMDIV | 0.63 | -5.95 | -51.73 | -62,11 | 6.64 | -67.74 | 4.94 | -65.89 | 5.25 | -66.81 | 5.61 | 0.00 | -3.71 |
| F687-1384 | CHEMDIV | 0.56 | -6.76 | -41.39 | -57,69 | 4.66 | -56.20 | 6.58 | -50.90 | 3.76 | -53.55 | 5.00 | 0.00 | -2.33 |
| F684-0404 | CHEMDIV | 0.56 | -5.91 | -41.52 | -34,38 | 5.19 | -40.42 | 4.11 | -63.95 | 6.46 | -52.18 | 5.25 | 0.00 | -2.19 |
| F294-0607 | CHEMDIV | 0.56 | -6.41 | -36.94 | -51,91 | 7.70 | -50.57 | 4.87 | -49.96 | 5.05 | -50.27 | 5.87 | 0.01 | -1.99 |
| Z1946972496 | Enamine | 0.62 | -6.70 | -48.85 | -46.29 | 3.15 | -49.35 | 6.25 | -46.40 | 5.88 | -47.35 | 5.09 | 0.01 | -1.68 |
| Z2595568325 | Enamine | 0.64 | -5.96 | -43.60 | -51.37 | 4.90 | -37.70 | 11.55 | -51.17 | 4.48 | -46.75 | 6.98 | 0.01 | -1.62 |
| AF-399/15285006 | SPECS | 0.56 | -5.85 | -43.92 | -36.71 | 4.80 | -51.11 | 6.85 | -52.42 | 7.35 | -46.74 | 6.33 | 0.01 | -1.62 |
| ZC47-0201 | CHEMDIV | 0.63 | -5.91 | -36.68 | -34,60 | 4.41 | -45.04 | 5.43 | -44.45 | 11.71 | -44.74 | 7.18 | 0.02 | -1.41 |
| F447-0692 | CHEMDIV | 0.52 | -5.84 | -37.54 | -36,48 | 4.86 | -42.47 | 4.44 | -43.00 | 5.45 | -42.73 | 4.92 | 0.02 | -1.20 |
| Z2607552291 | Enamine | 0.62 | -6.14 | -36.51 | -48.74 | 7.13 | -40.58 | 5.92 | -34.06 | 3.09 | -41.13 | 5.38 | 0.02 | -1.04 |
| G889-2420 | CHEMDIV | 0.52 | -6.36 | -32.78 | -34,63 | 5.78 | -43.09 | 5.25 | -34.17 | 11.59 | -38.63 | 7.54 | 0.03 | -0.78 |
| S841-0105 | CHEMDIV | 0.59 | -7.92 | -41.52 | -27,44 | 7.91 | -38.68 | 4.92 | -38.51 | 4.14 | -38.59 | 5.65 | 0.03 | -0.77 |
| AP-845/42110292 | SPECS | 0.78 | -5.73 | -42.72 | -37.78 | 3.21 | -32.74 | 5.12 | -45.16 | 5.59 | -38.56 | 4.64 | 0.03 | -0.77 |
| G889-1244 | CHEMDIV | 0.52 | -5.94 | -36.04 | -31,39 | 4.95 | -41.23 | 8.25 | -35.78 | 5.69 | -38.50 | 6.29 | 0.03 | -0.76 |
| F293-1056 | CHEMDIV | 0.56 | -6.89 | -44.49 | -34,46 | 9.36 | -30.96 | 10.62 | -45.67 | 5.18 | -38.32 | 8.39 | 0.03 | -0.74 |
| Y043-5143 | CHEMDIV | 0.51 | -5.97 | -28.11 | -45,62 | 11.44 | -34.27 | 7.46 | -41.86 | 3.84 | -38.07 | 7.58 | 0.03 | -0.72 |
| F687-0982 | CHEMDIV | 0.66 | -6.43 | -35.04 | -40,27 | 4.49 | -44.44 | 8.02 | -30.87 | 9.13 | -37.66 | 7.21 | 0.03 | -0.67 |
| F447-0515 | CHEMDIV | 0.57 | -6.92 | -44.28 | -36,98 | 5.83 | -39.05 | 5.57 | -35.42 | 3.90 | -37.23 | 5.10 | 0.03 | -0.63 |
| F293-1061 | CHEMDIV | 0.54 | -6.87 | -50.55 | -35,50 | 9.31 | -46.08 | 4.13 | -28.38 | 6.25 | -37.23 | 6.56 | 0.03 | -0.63 |
| AQ-360/42570853 | SPECS | 0.64 | -5.16 | -39.76 | -31.72 | 7.00 | -39.83 | 4.96 | -39.65 | 7.93 | -37.07 | 6.63 | 0.03 | -0.61 |
| AG-690/15438188 | SPECS | 0.68 | -5.28 | -38.29 | -40.25 | 7.96 | -35.29 | 6.40 | -32.86 | 5.69 | -36.14 | 6.69 | 0.04 | -0.52 |
| AN-465/43411026 | SPECS | 0.52 | -6.45 | -34.85 | -41.37 | 4.09 | -32.49 | 4.73 | -31.52 | 4.36 | -35.12 | 4.39 | 0.04 | -0.41 |
| AN-329/43386121 | SPECS | 0.61 | -5.53 | -37.51 | -29.25 | 7.55 | -41.70 | 4.92 | -32.37 | 5.61 | -34.44 | 6.03 | 0.04 | -0.34 |
| AN-329/43385389 | SPECS | 0.62 | -5.59 | -31.33 | -45.33 | 5.26 | -39.65 | 3.89 | -17.99 | 6.71 | -34.32 | 5.29 | 0.04 | -0.33 |
| AN-329/40942995 | SPECS | 0.70 | -5.87 | -45.27 | -33.21 | 6.43 | -30.88 | 7.24 | -37.80 | 6.79 | -33.96 | 6.82 | 0.04 | -0.29 |
| Z963573118 | Enamine | 0.67 | -6.59 | -38.72 | -24.87 | 9.34 | -47.78 | 3.22 | -28.25 | 7.18 | -33.63 | 6.58 | 0.04 | -0.25 |
| Z257643202 | Enamine | 0.50 | -6.80 | -37.05 | -36.37 | 3.40 | -33.51 | 5.44 | -30.91 | 4.64 | -33.59 | 4.49 | 0.04 | -0.25 |
| 8018-8675 | CHEMDIV | 0.60 | -7.42 | -28.55 | -32,66 | 5.62 | -27.86 | 4.49 | -39.02 | 4.19 | -33.44 | 4.77 | 0.04 | -0.23 |
| Z2627615568 | Enamine | 0.76 | -6.97 | -42.70 | -28.94 | 12.49 | -37.14 | 6.65 | -34.04 | 5.58 | -33.38 | 8.24 | 0.04 | -0.23 |
| Z1603470413 | Enamine | 0.66 | -6.25 | -49.40 | -37.62 | 5.43 | -33.11 | 7.81 | -28.52 | 10.77 | -33.09 | 8.00 | 0.04 | -0.20 |
| F724-0202 | CHEMDIV | 0.59 | -6.17 | -35.75 | -41,46 | 5.72 | -33.96 | 4.57 | -32.13 | 6.20 | -33.04 | 5.50 | 0.04 | -0.19 |
| AN-329/40945305 | SPECS | 0.81 | -6.22 | -38.52 | -36.75 | 8.79 | -32.64 | 5.34 | -28.46 | 6.28 | -32.61 | 6.80 | 0.04 | -0.15 |
| AQ-360/42570782 | SPECS | 0.53 | -5.76 | -38.90 | -39.00 | 5.22 | -27.45 | 6.43 | -30.98 | 6.52 | -32.48 | 6.06 | 0.04 | -0.13 |
| F447-0142 | CHEMDIV | 0.50 | -7.08 | -31.18 | -28,90 | 7.36 | -27.44 | 8.14 | -36.57 | 3.72 | -32.00 | 6.41 | 0.04 | -0.09 |
| G889-0607 | CHEMDIV | 0.51 | -6.71 | -35.47 | -19,91 | 8.29 | -29.27 | 5.53 | -34.47 | 5.65 | -31.87 | 6.49 | 0.04 | -0.07 |
| 8018-9039 | CHEMDIV | 0.65 | -7.32 | -27.50 | -34,06 | 6.20 | -33.95 | 4.19 | -29.43 | 4.17 | -31.69 | 4.86 | 0.04 | -0.05 |
| AG-690/15431824 | SPECS | 0.61 | -5.24 | -45.16 | -48.36 | 7.37 | -26.37 | 8.24 | -19.78 | 9.07 | -31.50 | 8.23 | 0.04 | -0.03 |
| CHEMBL3617209 | CHEMBL | 0.83 | -4.33 | -33.70 | -36.34 | 4.11 | -32.58 | 4.95 | -24.40 | 6.09 | -31.11 | 5.05 | 0.04 | 0.01 |
| Z398843372 | Enamine | 0.74 | -7.81 | -39.18 | -30.97 | 4.40 | -30.04 | 2.62 | -32.00 | 3.81 | -31.01 | 3.61 | 0.04 | 0.02 |
| 8602-0274 | CHEMDIV | 0.56 | -8.19 | -32.44 | -27,05 | 4.04 | -32.35 | 3.88 | -29.07 | 4.18 | -30.71 | 4.04 | 0.04 | 0.05 |
| AS-871/43475726 | SPECS | 0.56 | -5.46 | -29.73 | -24.87 | 8.10 | -33.47 | 3.67 | -33.23 | 9.64 | -30.52 | 7.14 | 0.04 | 0.07 |
| AG-690/13704110 | SPECS | 0.68 | -5.13 | -33.07 | -32.47 | 6.58 | -28.04 | 2.42 | -30.74 | 5.22 | -30.42 | 4.74 | 0.04 | 0.08 |
| Z1445399487 | Enamine | 0.55 | -5.91 | -46.09 | -23.05 | 7.02 | -34.14 | 9.05 | -31.80 | 4.62 | -29.66 | 6.89 | 0.04 | 0.16 |
| Z4114487168 | Enamine | 0.66 | -5.99 | -43.14 | -24.75 | 6.03 | -35.34 | 7.93 | -26.61 | 5.34 | -28.90 | 6.44 | 0.04 | 0.24 |
| AN-465/43460947 | SPECS | 0.59 | -6.74 | -28.06 | -27.87 | 3.85 | -28.96 | 4.82 | -28.09 | 3.88 | -28.31 | 4.18 | 0.04 | 0.30 |
| Z2504958912 | Enamine | 0.63 | -5.87 | -30.03 | -22.91 | 5.56 | -33.39 | 5.74 | -27.78 | 6.39 | -28.02 | 5.90 | 0.04 | 0.33 |
| G955-0482 | CHEMDIV | 0.54 | -6.33 | -41.86 | -21,50 | 8.67 | -26.87 | 5.82 | -28.00 | 5.84 | -27.44 | 6.77 | 0.04 | 0.39 |
| AF-399/41290240 | SPECS | 0.72 | -5.42 | -36.13 | -21.31 | 3.80 | -31.79 | 4.21 | -28.39 | 5.64 | -27.17 | 4.55 | 0.04 | 0.42 |
| AT-057/42952656 | SPECS | 0.66 | -5.68 | -22.91 | -19.96 | 9.59 | -25.69 | 7.93 | -35.28 | 4.35 | -26.98 | 7.29 | 0.04 | 0.44 |
| AK-968/15361749 | SPECS | 0.54 | -6.21 | -41.33 | -21.12 | 7.05 | -33.27 | 5.10 | -25.73 | 6.60 | -26.71 | 6.25 | 0.04 | 0.47 |
| Z1609738788 | Enamine | 0.82 | -6.05 | -45.79 | -29.36 | 8.68 | -22.61 | 5.17 | -25.08 | 9.86 | -25.68 | 7.90 | 0.04 | 0.57 |
| AN-329/43448881 | SPECS | 0.67 | -6.82 | -37.07 | -35.08 | 7.52 | -10.32 | 11.21 | -30.49 | 5.76 | -25.30 | 8.16 | 0.03 | 0.61 |
| Z2754552007 | Enamine | 0.58 | -6.34 | -37.86 | -27.00 | 9.78 | -23.08 | 7.23 | -25.09 | 13.28 | -25.06 | 10.10 | 0.03 | 0.64 |
| Z1227711106 | Enamine | 0.70 | -6.09 | -34.80 | -11.16 | 8.62 | -44.53 | 6.31 | -16.47 | 5.47 | -24.05 | 6.80 | 0.03 | 0.74 |
| Z1517547057 | Enamine | 0.58 | -8.04 | -36.31 | -26.53 | 5.96 | -22.23 | 4.85 | -23.34 | 5.75 | -24.04 | 5.52 | 0.03 | 0.75 |
| AB-323/13887094 | SPECS | 0.83 | -5.98 | -42.52 | -26.18 | 5.31 | -29.14 | 4.76 | -16.56 | 9.04 | -23.96 | 6.37 | 0.03 | 0.75 |
| AN-329/10547048 | SPECS | 0.53 | -5.34 | -28.80 | -24.82 | 4.44 | -21.95 | 11.04 | -24.31 | 6.71 | -23.69 | 7.40 | 0.03 | 0.78 |
| AJ-292/41685910 | SPECS | 0.80 | -5.21 | -27.18 | -28.18 | 7.26 | -13.12 | 6.20 | -27.63 | 5.07 | -22.98 | 6.18 | 0.03 | 0.86 |
| Z1445505436 | Enamine | 0.79 | -6.43 | -22.89 | -14.22 | 5.83 | -23.73 | 4.25 | -30.40 | 5.07 | -22.78 | 5.05 | 0.03 | 0.88 |
| Z963572756 | Enamine | 0.78 | -6.33 | -44.51 | -24.24 | 9.40 | -20.98 | 5.62 | -22.94 | 4.70 | -22.72 | 6.58 | 0.03 | 0.88 |
| AJ-030/14523202 | SPECS | 0.68 | -6.06 | -53.37 | -15.29 | 17.06 | -25.12 | 10.81 | -27.32 | 10.35 | -22.58 | 12.74 | 0.03 | 0.90 |
| AU-059/02895030 | SPECS | 0.86 | -6.11 | -45.33 | -25.58 | 9.86 | -12.64 | 13.70 | -28.87 | 6.05 | -22.36 | 9.87 | 0.03 | 0.92 |
| Z2489598461 | Enamine | 0.64 | -6.76 | -31.01 | -22.02 | 11.54 | -22.89 | 5.07 | -21.60 | 6.09 | -22.17 | 7.57 | 0.03 | 0.94 |
| Y203-9173 | CHEMDIV | 0.54 | -5.87 | -39.65 | -31,63 | 3.85 | -27.31 | 7.53 | -16.98 | 9.46 | -22.14 | 6.95 | 0.03 | 0.94 |
| Z1635315655 | Enamine | 0.50 | -6.31 | -18.91 | -38.90 | 3.89 | -12.11 | 8.74 | -15.40 | 9.35 | -22.13 | 7.33 | 0.03 | 0.94 |
| Z1159207962 | Enamine | 0.85 | -8.23 | -33.05 | -26.81 | 3.21 | -15.83 | 8.84 | -22.78 | 3.85 | -21.81 | 5.30 | 0.03 | 0.98 |
| F294-1004 | CHEMDIV | 0.54 | -5.95 | -37.48 | -38,81 | 6.70 | -22.72 | 9.36 | -19.98 | 12.89 | -21.35 | 9.65 | 0.02 | 1.03 |
| Z2911448235 | Enamine | 0.63 | -7.87 | -42.87 | -17.68 | 5.61 | -21.90 | 4.96 | -23.96 | 4.20 | -21.18 | 4.93 | 0.02 | 1.04 |
| G696-5966 | CHEMDIV | 0.66 | -8.09 | -41.50 | -28,64 | 6.10 | -22.14 | 12.53 | -18.83 | 11.49 | -20.49 | 10.04 | 0.02 | 1.12 |
| AN-465/43424886 | SPECS | 0.61 | -7.47 | -35.01 | -15.85 | 8.54 | -15.27 | 9.83 | -28.48 | 3.46 | -19.87 | 7.28 | 0.02 | 1.18 |
| Z2796658937 | Enamine | 0.68 | -6.12 | -31.51 | -10.03 | 11.98 | -31.81 | 4.46 | -14.87 | 6.20 | -18.90 | 7.55 | 0.02 | 1.28 |
| Z2607716151 | Enamine | 0.57 | -7.55 | -39.77 | -24.29 | 6.86 | -8.73 | 9.56 | -20.58 | 6.61 | -17.87 | 7.68 | 0.02 | 1.39 |
| Z808565802 | Enamine | 0.72 | -6.78 | -28.37 | -24.88 | 9.05 | -15.12 | 7.19 | -12.47 | 7.35 | -17.49 | 7.87 | 0.02 | 1.43 |
| C197-0573 | CHEMDIV | 0.57 | -7.17 | -34.92 | -49,41 | 6.94 | -21.53 | 4.46 | -12.29 | 9.32 | -16.91 | 6.91 | 0.01 | 1.49 |
| Z3173453002 | Enamine | 0.78 | -6.21 | -23.64 | -15.83 | 6.38 | -21.16 | 4.53 | -13.55 | 9.96 | -16.85 | 6.96 | 0.01 | 1.49 |
| AI-204/05459031 | SPECS | 0.53 | -5.87 | -31.37 | -10.21 | 7.28 | -12.10 | 6.98 | -18.83 | 3.95 | -13.71 | 6.07 | 0.01 | 1.82 |

Table S 5: The predicted anticancer activity, docking scores, post-virtual screening workflow average MM/GBSA scores, and the average MM/GBSA scores of the 3 short MD runs performed on the top 25 molecules obtained from each library in the ERCC1/XPF protein complex’s binding site 2

| **Ligand ID** | **Library** | **Anticancer Activity Prediction** | **Docking Score (kcal/mol)** | **Post-VSW MM/GBSA (kcal/mol)** | **MM/GBSA Run 1 (kcal/mol)** | **Standard Deviation Run 1** | **MM/GBSA Run 2 (kcal/mol)** | **Standard Deviation Run 2** | **MM/GBSA Run 3 (kcal/mol)** | **Standard Deviation Run 3** | **MMGBSA Average (kcal/mol)** | **Standard Deviation Average** | **Normal Distribution** | **Z-score** |
| --- | --- | --- | --- | --- | --- | --- | --- | --- | --- | --- | --- | --- | --- | --- |
| 0527-0155 | CHEMDIV | 0.72 | -5.75 | -37.63 | -41.35 | 3.71 | -41.76 | 3.65 | -28.33 | 9.21 | -37.15 | 5.52 | 0.00 | -2.73 |
| Z74543901 | Enamine | 0.60 | -4.29 | -22.93 | -36.18 | 6.73 | -24.74 | 3.20 | -34.34 | 7.87 | -31.76 | 5.93 | 0.01 | -1.84 |
| Z807707434 | Enamine | 0.71 | -3.85 | -38.43 | -26.55 | 7.05 | -28.41 | 9.61 | -38.22 | 6.72 | -31.06 | 7.79 | 0.01 | -1.72 |
| AG-205/37107123 | SPECS | 0.60 | -3.82 | -39.83 | -26.20 | 6.43 | -26.90 | 5.48 | -37.93 | 6.91 | -30.35 | 6.27 | 0.02 | -1.61 |
| AI-031/31967053 | SPECS | 0.57 | -4.99 | -17.28 | -34.50 | 10.90 | -34.11 | 9.01 | -19.55 | 4.07 | -29.39 | 7.99 | 0.02 | -1.45 |
| 8013-1758 | CHEMDIV | 0.79 | -3.38 | -33.54 | -27.02 | 6.93 | -29.40 | 4.87 | -31.46 | 4.94 | -29.29 | 5.58 | 0.02 | -1.43 |
| 8020-0379 | CHEMDIV | 0.59 | -3.40 | -23.78 | -35.37 | 6.64 | -21.81 | 6.20 | -24.82 | 5.54 | -27.33 | 6.13 | 0.04 | -1.11 |
| AE-562/12222311 | SPECS | 0.54 | -3.61 | -21.15 | -25.56 | 7.02 | -31.44 | 7.77 | -24.55 | 6.38 | -27.19 | 7.06 | 0.04 | -1.08 |
| 8017-1136 | CHEMDIV | 0.50 | -3.86 | -18.46 | -21.15 | 5.70 | -32.58 | 4.74 | -27.60 | 6.31 | -27.11 | 5.58 | 0.04 | -1.07 |
| Z1445395750 | Enamine | 0.64 | -5.40 | -20.16 | -25.58 | 4.34 | -25.40 | 5.42 | -27.65 | 5.23 | -26.21 | 5.00 | 0.04 | -0.92 |
| 8016-0721 | CHEMDIV | 0.90 | -3.93 | -31.68 | -22.86 | 6.70 | -30.91 | 7.29 | -23.85 | 7.96 | -25.87 | 7.32 | 0.05 | -0.87 |
| AP-853/43387217 | SPECS | 0.76 | -4.22 | -24.34 | -26.45 | 9.84 | -16.87 | 4.27 | -34.14 | 8.33 | -25.82 | 7.48 | 0.05 | -0.86 |
| Z1675850257 | Enamine | 0.63 | -3.80 | -28.15 | -22.11 | 11.42 | -23.35 | 10.17 | -29.63 | 6.75 | -25.03 | 9.45 | 0.05 | -0.73 |
| Z1758869753 | Enamine | 0.73 | -3.78 | -29.49 | -22.67 | 11.71 | -25.51 | 8.18 | -26.08 | 4.38 | -24.75 | 8.09 | 0.05 | -0.68 |
| 8009-7841 | CHEMDIV | 0.54 | -3.51 | -30.17 | -24.58 | 6.29 | -29.78 | 3.79 | -19.75 | 13.71 | -24.71 | 7.93 | 0.05 | -0.67 |
| Z1274460823 | Enamine | 0.81 | -5.27 | -22.19 | -21.92 | 4.64 | -26.32 | 5.97 | -25.74 | 5.42 | -24.66 | 5.34 | 0.05 | -0.67 |
| Z274574446 | Enamine | 0.56 | -4.84 | -17.82 | -29.96 | 7.15 | -11.90 | 9.40 | -32.11 | 4.50 | -24.66 | 7.02 | 0.05 | -0.67 |
| AG-205/40650580 | SPECS | 0.61 | -5.00 | -19.92 | -23.62 | 5.58 | -24.26 | 5.80 | -25.95 | 5.81 | -24.61 | 5.73 | 0.05 | -0.66 |
| AU-059/43516942 | SPECS | 0.89 | -4.53 | -28.55 | -20.05 | 6.24 | -35.59 | 11.86 | -17.88 | 10.63 | -24.51 | 9.58 | 0.05 | -0.64 |
| Z234809834 | Enamine | 0.81 | -4.99 | -16.64 | -18.90 | 3.26 | -28.67 | 5.36 | -25.77 | 6.12 | -24.44 | 4.91 | 0.05 | -0.63 |
| AB-323/13887107 | SPECS | 0.66 | -4.21 | -18.16 | -30.86 | 7.65 | -12.59 | 8.64 | -29.21 | 6.63 | -24.22 | 7.64 | 0.06 | -0.59 |
| S836-7339 | CHEMDIV | 0.55 | -3.67 | -35.66 | -36.03 | 8.23 | -14.02 | 10.18 | -22.42 | 7.53 | -24.16 | 8.65 | 0.06 | -0.58 |
| Z636475366 | Enamine | 0.59 | -3.73 | -24.20 | -26.50 | 7.18 | -22.85 | 5.20 | -22.74 | 10.75 | -24.03 | 7.71 | 0.06 | -0.56 |
| AQ-086/43478992 | SPECS | 0.56 | -6.82 | -31.77 | -24.58 | 7.26 | -25.59 | 7.29 | -21.73 | 4.77 | -23.97 | 6.44 | 0.06 | -0.55 |
| Y021-1697 | CHEMDIV | 0.55 | -4.06 | -25.27 | -18.38 | 6.60 | -27.28 | 6.82 | -25.85 | 7.92 | -23.84 | 7.11 | 0.06 | -0.53 |
| 2460-0811 | CHEMDIV | 0.67 | -3.77 | -27.04 | -21.02 | 10.37 | -30.87 | 2.62 | -19.58 | 9.86 | -23.82 | 7.62 | 0.06 | -0.53 |
| AJ-292/13525008 | SPECS | 0.52 | -5.74 | -22.30 | -17.19 | 4.10 | -20.80 | 8.38 | -33.08 | 6.08 | -23.69 | 6.19 | 0.06 | -0.51 |
| 8015-7008 | CHEMDIV | 0.78 | -3.79 | -17.37 | -18.59 | 8.30 | -23.08 | 4.11 | -28.81 | 5.81 | -23.49 | 6.07 | 0.06 | -0.47 |
| AO-080/43378394 | SPECS | 0.54 | -4.43 | -18.30 | -35.20 | 7.39 | -18.25 | 4.39 | -16.84 | 5.14 | -23.43 | 5.64 | 0.06 | -0.46 |
| Y040-8817 | CHEMDIV | 0.65 | -4.54 | -23.33 | -25.25 | 5.75 | -24.92 | 6.00 | -18.61 | 5.31 | -22.93 | 5.69 | 0.06 | -0.38 |
| CHEMBL3617209 | CHEMBL | 0.83 | -3.86 | -20.02 | -36.57 | 9.17 | -5.19 | 7.45 | -25.98 | 6.08 | -22.58 | 7.57 | 0.06 | -0.32 |
| Z234988436 | Enamine | 0.81 | -5.01 | -18.17 | -27.01 | 11.03 | -19.02 | 3.77 | -20.26 | 4.03 | -22.10 | 6.28 | 0.06 | -0.24 |
| Z2996050370 | Enamine | 0.51 | -4.02 | -22.44 | -18.39 | 5.92 | -14.99 | 5.18 | -32.35 | 5.40 | -21.91 | 5.50 | 0.06 | -0.21 |
| 4478-3729 | CHEMDIV | 0.80 | -3.38 | -27.64 | -18.13 | 9.47 | -21.44 | 4.72 | -25.90 | 7.38 | -21.82 | 7.19 | 0.06 | -0.20 |
| Z2448763957 | Enamine | 0.76 | -3.80 | -28.80 | -28.28 | 7.29 | -11.79 | 12.61 | -25.17 | 3.69 | -21.74 | 7.86 | 0.06 | -0.18 |
| 8014-1463 | CHEMDIV | 0.91 | -3.42 | -15.88 | -21.59 | 4.33 | -23.04 | 6.84 | -20.60 | 3.42 | -21.74 | 4.86 | 0.06 | -0.18 |
| 0133-0003 | CHEMDIV | 0.88 | -3.99 | -18.72 | -27.18 | 12.82 | -25.38 | 10.07 | -12.32 | 6.63 | -21.63 | 9.84 | 0.06 | -0.16 |
| K085-0019 | CHEMDIV | 0.56 | -3.49 | -19.02 | -22.15 | 8.54 | -16.94 | 6.73 | -25.64 | 5.98 | -21.58 | 7.08 | 0.07 | -0.16 |
| Z2761825433 | Enamine | 0.51 | -4.45 | -23.36 | -16.32 | 8.97 | -25.81 | 5.36 | -22.49 | 5.83 | -21.54 | 6.72 | 0.07 | -0.15 |
| Z2581731665 | Enamine | 0.65 | -4.33 | -18.52 | -30.60 | 8.93 | -12.36 | 6.79 | -21.49 | 3.61 | -21.48 | 6.44 | 0.07 | -0.14 |
| AS-662/43412951 | SPECS | 0.79 | -3.99 | -24.90 | -17.69 | 7.10 | -19.87 | 6.45 | -26.66 | 6.48 | -21.41 | 6.68 | 0.07 | -0.13 |
| Z2476547507 | Enamine | 0.62 | -4.20 | -27.60 | -24.08 | 5.56 | -21.33 | 3.91 | -18.44 | 7.02 | -21.28 | 5.49 | 0.07 | -0.11 |
| AG-690/11669147 | SPECS | 0.66 | -5.15 | -18.63 | -24.97 | 6.47 | -22.52 | 3.69 | -15.59 | 5.57 | -21.03 | 5.24 | 0.07 | -0.07 |
| Z2788447560 | Enamine | 0.59 | -4.34 | -22.47 | -18.21 | 8.85 | -22.15 | 5.45 | -22.41 | 6.36 | -20.92 | 6.89 | 0.07 | -0.05 |
| Z1350778087 | Enamine | 0.81 | -4.64 | -22.16 | -17.23 | 6.50 | -30.03 | 5.24 | -14.97 | 5.04 | -20.75 | 5.59 | 0.07 | -0.02 |
| Z283989490 | Enamine | 0.56 | -4.27 | -22.57 | -7.67 | 8.54 | -26.67 | 6.27 | -27.54 | 2.97 | -20.63 | 5.93 | 0.07 | 0.00 |
| AQ-360/11426152 | SPECS | 0.53 | -4.71 | -21.84 | -13.57 | 7.67 | -21.61 | 4.12 | -25.25 | 10.64 | -20.14 | 7.48 | 0.07 | 0.08 |
| AU-059/02895030 | SPECS | 0.86 | -5.14 | -26.23 | -19.02 | 6.38 | -14.30 | 8.11 | -26.83 | 5.13 | -20.05 | 6.54 | 0.07 | 0.10 |
| AB-323/13887094 | SPECS | 0.83 | -5.64 | -28.33 | -21.69 | 4.64 | -7.34 | 9.67 | -31.00 | 4.27 | -20.01 | 6.19 | 0.07 | 0.10 |
| R052-0872 | CHEMDIV | 0.82 | -3.96 | -17.73 | -16.25 | 5.61 | -19.92 | 5.60 | -22.87 | 5.58 | -19.68 | 5.60 | 0.07 | 0.16 |
| Z2396480061 | Enamine | 0.82 | -3.79 | -31.94 | -28.45 | 7.61 | -17.50 | 9.63 | -10.98 | 9.97 | -18.98 | 9.07 | 0.06 | 0.27 |
| Z1983689834 | Enamine | 0.81 | -6.16 | -26.71 | -18.12 | 5.50 | -18.15 | 5.12 | -20.30 | 7.81 | -18.86 | 6.14 | 0.06 | 0.29 |
| Z2506674678 | Enamine | 0.72 | -4.17 | -19.93 | -20.19 | 7.18 | -18.16 | 6.42 | -16.56 | 2.73 | -18.30 | 5.44 | 0.06 | 0.38 |
| 8756-0077 | CHEMDIV | 0.68 | -3.81 | -17.21 | -15.92 | 9.00 | -25.97 | 4.34 | -12.00 | 5.29 | -17.96 | 6.21 | 0.06 | 0.44 |
| AI-204/31688002 | SPECS | 0.82 | -4.37 | -18.91 | -11.42 | 7.61 | -24.31 | 5.61 | -17.58 | 4.07 | -17.77 | 5.76 | 0.06 | 0.47 |
| C202-2389 | CHEMDIV | 0.53 | -3.47 | -23.96 | -22.13 | 4.71 | -12.34 | 8.77 | -16.81 | 4.88 | -17.09 | 6.12 | 0.06 | 0.58 |
| Y044-6691 | CHEMDIV | 0.55 | -4.01 | -19.31 | -17.30 | 9.51 | -22.31 | 3.45 | -11.22 | 7.43 | -16.94 | 6.80 | 0.05 | 0.61 |
| 8016-3256 | CHEMDIV | 0.62 | -4.54 | -22.34 | -9.41 | 9.72 | -19.89 | 3.55 | -20.59 | 4.30 | -16.63 | 5.86 | 0.05 | 0.66 |
| AN-465/43384045 | SPECS | 0.61 | -3.56 | -22.14 | -13.66 | 8.78 | -21.87 | 8.18 | -13.45 | 9.88 | -16.32 | 8.95 | 0.05 | 0.71 |
| 2812-0053 | CHEMDIV | 0.54 | -4.09 | -14.39 | -13.48 | 5.12 | -16.35 | 4.87 | -18.08 | 3.13 | -15.97 | 4.37 | 0.05 | 0.77 |
| Z1523160623 | Enamine | 0.84 | -4.42 | -32.79 | -14.51 | 13.23 | -18.65 | 10.66 | -14.13 | 12.52 | -15.76 | 12.14 | 0.05 | 0.80 |
| K629-0034 | CHEMDIV | 0.84 | -3.92 | -24.37 | -10.86 | 10.08 | -18.10 | 3.82 | -15.62 | 12.74 | -14.86 | 8.88 | 0.04 | 0.95 |
| Z2946794372 | Enamine | 0.57 | -4.00 | -20.17 | -23.06 | 9.30 | -11.58 | 9.54 | -8.56 | 8.49 | -14.40 | 9.11 | 0.04 | 1.03 |
| AO-022/43513452 | SPECS | 0.61 | -3.77 | -10.73 | -9.07 | 4.06 | -18.52 | 3.12 | -12.50 | 3.03 | -13.36 | 3.40 | 0.03 | 1.20 |
| AE-848/00990055 | SPECS | 0.82 | -4.11 | -14.70 | -12.88 | 5.25 | -14.93 | 7.11 | -11.21 | 7.27 | -13.00 | 6.54 | 0.03 | 1.26 |
| Y020-9755 | CHEMDIV | 0.55 | -3.42 | -8.70 | -11.42 | 1.86 | -7.61 | 6.53 | -19.74 | 7.56 | -12.92 | 5.32 | 0.03 | 1.27 |
| Z2763053782 | Enamine | 0.50 | -5.05 | -17.86 | -4.37 | 5.77 | -9.93 | 5.85 | -22.71 | 4.42 | -12.34 | 5.35 | 0.03 | 1.37 |
| 8013-1619 | CHEMDIV | 0.65 | -3.63 | -28.79 | -15.19 | 10.26 | -12.58 | 8.84 | -8.20 | 9.86 | -11.99 | 9.66 | 0.02 | 1.43 |
| AE-848/30701010 | SPECS | 0.88 | -3.79 | -7.94 | -12.61 | 10.42 | -13.35 | 3.69 | -8.83 | 8.50 | -11.59 | 7.54 | 0.02 | 1.49 |
| 8013-0959 | CHEMDIV | 0.84 | -3.54 | -24.37 | -5.95 | 8.51 | -16.58 | 6.45 | -11.00 | 7.86 | -11.17 | 7.60 | 0.02 | 1.56 |
| AN-584/40178121 | SPECS | 0.90 | -3.82 | -25.82 | -3.84 | 7.47 | -10.14 | 8.94 | -18.87 | 10.44 | -10.95 | 8.95 | 0.02 | 1.60 |
| AP-853/43464284 | SPECS | 0.85 | -6.00 | -23.07 | -10.33 | 6.18 | -8.30 | 6.98 | -13.06 | 6.66 | -10.56 | 6.61 | 0.02 | 1.66 |
| Z2581731965 | Enamine | 0.63 | -4.49 | -18.06 | -19.18 | 10.14 | -2.58 | 5.81 | -8.18 | 7.48 | -9.98 | 7.81 | 0.01 | 1.76 |
| AE-848/31946053 | SPECS | 0.77 | -4.23 | -21.22 | -7.41 | 7.06 | -15.97 | 5.58 | -6.07 | 6.79 | -9.82 | 6.48 | 0.01 | 1.78 |
| AK-968/15359162 | SPECS | 0.62 | -5.69 | -18.72 | -10.57 | 6.00 | -5.31 | 6.33 | -9.76 | 7.01 | -8.55 | 6.45 | 0.01 | 1.99 |
| AE-848/00964025 | SPECS | 0.55 | -4.70 | -14.77 | -3.01 | 5.13 | -2.73 | 4.98 | -6.47 | 6.15 | -4.07 | 5.42 | 0.00 | 2.73 |

Table S 6: The predicted anticancer activity, docking scores, post-virtual screening workflow average MM/GBSA scores, and the average MM/GBSA scores of the 3 short MD runs performed on the top 25 molecules obtained from each library in the ERCC1/XPF protein complex’s binding site 3

| **Ligand ID** | **Library** | **Anticancer Activity Prediction** | **Docking Score (kcal/mol)** | **Post-VSW MM/GBSA (kcal/mol)** | **MM/GBSA Run 1 (kcal/mol)** | **Standard Deviation Run 1** | **MM/GBSA Run 2 (kcal/mol)** | **Standard Deviation Run 2** | **MM/GBSA Run 3 (kcal/mol)** | **Standard Deviation Run 3** | **MMGBSA Average (kcal/mol)** | **Standard Deviation Average** | **Normal Distribution** | **Z-score** |
| --- | --- | --- | --- | --- | --- | --- | --- | --- | --- | --- | --- | --- | --- | --- |
| V008-1978 | CHEMDIV | 0.64 | -6.23 | -60.16 | -60.07 | 5.42 | -71.61 | 6.99 | -58.95 | 6.52 | -63.54 | 6.31 | 0.01 | -1.58 |
| AQ-405/42300197 | SPECS | 0.59 | -5.59 | -58.27 | -49.26 | 8.15 | -64.69 | 6.55 | -75.86 | 10.51 | -63.27 | 8.40 | 0.01 | -1.55 |
| AG-205/36628032 | SPECS | 0.65 | -5.65 | -48.58 | -73.59 | 11.86 | -63.23 | 9.93 | -52.35 | 6.51 | -63.06 | 9.43 | 0.01 | -1.53 |
| AQ-405/42300228 | SPECS | 0.62 | -6.10 | -49.29 | -52.30 | 8.14 | -69.47 | 4.55 | -66.48 | 7.04 | -62.75 | 6.57 | 0.01 | -1.50 |
| AN-465/41990317 | SPECS | 0.54 | -5.38 | -43.78 | -67.41 | 7.78 | -60.59 | 7.32 | -59.55 | 4.90 | -62.52 | 6.67 | 0.02 | -1.47 |
| AO-476/43417634 | SPECS | 0.78 | -5.20 | -53.09 | -58.02 | 4.51 | -62.89 | 5.00 | -64.01 | 5.53 | -61.64 | 5.01 | 0.02 | -1.37 |
| 8014-8414 | CHEMDIV | 0.51 | -5.80 | -70.15 | -63.28 | 5.57 | -58.17 | 6.94 | -63.44 | 10.42 | -61.63 | 7.64 | 0.02 | -1.37 |
| G696-5919 | CHEMDIV | 0.66 | -5.91 | -49.34 | -61.39 | 7.63 | -71.11 | 5.39 | -49.23 | 20.75 | -60.58 | 11.26 | 0.02 | -1.26 |
| AK-968/41923847 | SPECS | 0.56 | -5.21 | -62.67 | -58.99 | 4.99 | -69.48 | 9.24 | -53.16 | 5.89 | -60.54 | 6.71 | 0.02 | -1.25 |
| S596-0645 | CHEMDIV | 0.70 | -6.07 | -50.94 | -55.58 | 10.07 | -61.13 | 9.23 | -63.51 | 6.58 | -60.07 | 8.63 | 0.02 | -1.20 |
| G856-5453 | CHEMDIV | 0.66 | -5.74 | -45.61 | -55.12 | 6.92 | -53.01 | 4.78 | -71.54 | 7.46 | -59.89 | 6.39 | 0.02 | -1.18 |
| V023-3592 | CHEMDIV | 0.70 | -5.80 | -50.59 | -86.86 | 7.24 | -49.80 | 8.63 | -42.64 | 7.18 | -59.77 | 7.68 | 0.02 | -1.17 |
| F154-0589 | CHEMDIV | 0.71 | -5.74 | -52.15 | -64.89 | 8.28 | -64.99 | 7.77 | -48.73 | 7.86 | -59.53 | 7.97 | 0.02 | -1.14 |
| AS-980/43410531 | SPECS | 0.57 | -5.14 | -24.20 | -65.95 | 7.03 | -54.82 | 5.95 | -57.45 | 7.57 | -59.41 | 6.85 | 0.02 | -1.13 |
| Z787549448 | Enamine | 0.75 | -5.67 | -46.44 | -60.17 | 3.85 | -48.65 | 6.11 | -67.17 | 4.60 | -58.66 | 4.85 | 0.03 | -1.04 |
| AK-968/41924756 | SPECS | 0.54 | -5.49 | -43.78 | -66.48 | 6.42 | -49.77 | 10.52 | -56.64 | 11.26 | -57.63 | 9.40 | 0.03 | -0.93 |
| AN-740/37278013 | SPECS | 0.65 | -5.17 | -42.47 | -66.54 | 4.45 | -53.90 | 7.98 | -51.01 | 5.95 | -57.15 | 6.12 | 0.03 | -0.87 |
| AO-476/43407204 | SPECS | 0.64 | -5.57 | -45.27 | -51.17 | 5.93 | -60.83 | 6.77 | -58.40 | 6.64 | -56.80 | 6.45 | 0.03 | -0.84 |
| Z956158858 | Enamine | 0.61 | -5.71 | -44.50 | -60.68 | 6.41 | -62.74 | 6.15 | -46.88 | 4.77 | -56.77 | 5.78 | 0.03 | -0.83 |
| Z1332317213 | Enamine | 0.72 | -5.66 | -32.22 | -65.09 | 6.90 | -60.55 | 5.77 | -44.52 | 4.36 | -56.72 | 5.68 | 0.03 | -0.83 |
| Z1824174370 | Enamine | 0.63 | -5.70 | -38.15 | -57.08 | 4.67 | -53.66 | 5.00 | -58.84 | 6.53 | -56.53 | 5.40 | 0.03 | -0.81 |
| SD06-0865 | CHEMDIV | 0.50 | -5.77 | -41.35 | -63.79 | 6.45 | -55.79 | 7.74 | -49.53 | 5.26 | -56.37 | 6.48 | 0.03 | -0.79 |
| AN-988/15131264 | SPECS | 0.61 | -5.59 | -52.39 | -58.62 | 8.14 | -53.05 | 8.82 | -52.71 | 8.14 | -54.79 | 8.36 | 0.04 | -0.61 |
| Z822253918 | Enamine | 0.54 | -5.64 | -24.06 | -52.42 | 7.03 | -57.37 | 6.12 | -54.30 | 5.38 | -54.70 | 6.18 | 0.04 | -0.60 |
| AO-022/43512474 | SPECS | 0.58 | -5.35 | -40.50 | -48.95 | 5.11 | -58.19 | 4.07 | -56.92 | 4.55 | -54.69 | 4.58 | 0.04 | -0.60 |
| V022-7820 | CHEMDIV | 0.79 | -5.90 | -33.68 | -53.60 | 5.99 | -49.30 | 7.01 | -60.74 | 6.94 | -54.55 | 6.65 | 0.04 | -0.59 |
| AN-465/42833800 | SPECS | 0.52 | -5.10 | -40.19 | -58.11 | 4.97 | -51.89 | 5.66 | -51.93 | 6.32 | -53.98 | 5.65 | 0.04 | -0.52 |
| Z2767890599 | Enamine | 0.68 | -5.60 | -41.40 | -62.08 | 5.63 | -52.98 | 7.72 | -46.14 | 8.10 | -53.73 | 7.15 | 0.04 | -0.50 |
| Z2234970955 | Enamine | 0.61 | -5.84 | -37.13 | -50.43 | 3.93 | -56.56 | 7.44 | -53.98 | 6.93 | -53.66 | 6.10 | 0.04 | -0.49 |
| Y043-5532 | CHEMDIV | 0.56 | -5.82 | -42.54 | -56.20 | 5.33 | -53.62 | 6.99 | -48.25 | 9.53 | -52.69 | 7.28 | 0.04 | -0.38 |
| AP-263/43503625 | SPECS | 0.69 | -5.20 | -51.52 | -44.03 | 6.88 | -50.49 | 5.81 | -63.18 | 5.81 | -52.57 | 6.17 | 0.04 | -0.37 |
| AT-057/43315937 | SPECS | 0.65 | -5.10 | -44.87 | -63.30 | 10.23 | -48.50 | 8.90 | -45.78 | 6.42 | -52.53 | 8.52 | 0.04 | -0.36 |
| AN-465/43369904 | SPECS | 0.59 | -5.25 | -51.32 | -50.94 | 3.89 | -52.23 | 6.31 | -53.01 | 4.81 | -52.06 | 5.00 | 0.04 | -0.31 |
| V028-2225 | CHEMDIV | 0.63 | -6.38 | -48.14 | -47.95 | 5.97 | -45.86 | 7.28 | -57.11 | 7.27 | -50.30 | 6.84 | 0.04 | -0.11 |
| S596-1358 | CHEMDIV | 0.71 | -6.32 | -45.67 | -55.99 | 5.30 | -45.01 | 10.44 | -49.17 | 5.01 | -50.06 | 6.92 | 0.04 | -0.09 |
| Z2905660614 | Enamine | 0.59 | -5.65 | -34.92 | -51.51 | 6.90 | -40.42 | 15.83 | -56.49 | 5.93 | -49.47 | 9.55 | 0.04 | -0.02 |
| SB11-0097 | CHEMDIV | 0.67 | -5.81 | -43.86 | -56.18 | 5.13 | -52.22 | 7.73 | -39.65 | 8.19 | -49.35 | 7.02 | 0.04 | -0.01 |
| 0831-0948 | CHEMDIV | 0.60 | -6.01 | -40.07 | -49.38 | 4.82 | -48.60 | 6.15 | -49.61 | 6.09 | -49.20 | 5.68 | 0.04 | 0.01 |
| R015-0010 | CHEMDIV | 0.56 | -6.15 | -49.60 | -38.76 | 9.48 | -52.63 | 6.84 | -55.44 | 4.08 | -48.94 | 6.80 | 0.04 | 0.04 |
| Z916003148 | Enamine | 0.54 | -5.71 | -52.51 | -30.69 | 15.37 | -62.35 | 8.12 | -53.68 | 6.24 | -48.91 | 9.91 | 0.04 | 0.04 |
| Z2062028767 | Enamine | 0.65 | -5.67 | -37.76 | -52.02 | 8.41 | -50.13 | 5.49 | -44.53 | 6.31 | -48.90 | 6.74 | 0.04 | 0.04 |
| AN-329/40922877 | SPECS | 0.56 | -5.20 | -56.23 | -50.04 | 4.80 | -41.65 | 8.41 | -54.95 | 6.28 | -48.88 | 6.50 | 0.04 | 0.04 |
| AN-465/43411181 | SPECS | 0.57 | -5.34 | -30.14 | -52.89 | 4.93 | -43.84 | 6.95 | -49.30 | 6.67 | -48.67 | 6.18 | 0.04 | 0.07 |
| SA96-1275 | CHEMDIV | 0.56 | -5.73 | -50.72 | -48.10 | 5.63 | -59.09 | 6.33 | -38.15 | 6.86 | -48.45 | 6.27 | 0.04 | 0.09 |
| Z2464461011 | Enamine | 0.67 | -5.63 | -48.17 | -46.14 | 4.77 | -49.88 | 6.21 | -47.89 | 5.50 | -47.97 | 5.49 | 0.04 | 0.14 |
| 8006-2504 | CHEMDIV | 0.90 | -5.79 | -37.63 | -53.03 | 5.87 | -37.93 | 9.55 | -51.92 | 5.06 | -47.63 | 6.83 | 0.04 | 0.18 |
| Z1636704680 | Enamine | 0.78 | -5.55 | -31.94 | -42.03 | 5.85 | -54.54 | 7.23 | -45.65 | 8.19 | -47.41 | 7.09 | 0.04 | 0.21 |
| V013-0150 | CHEMDIV | 0.64 | -5.71 | -35.62 | -48.59 | 7.39 | -46.66 | 7.80 | -45.71 | 7.31 | -46.99 | 7.50 | 0.04 | 0.25 |
| Z1150656136 | Enamine | 0.65 | -6.04 | -32.89 | -44.99 | 8.13 | -48.45 | 3.82 | -46.71 | 5.02 | -46.72 | 5.66 | 0.04 | 0.28 |
| Z1011714984 | Enamine | 0.88 | -5.71 | -36.44 | -44.22 | 6.19 | -37.13 | 7.60 | -58.64 | 6.54 | -46.67 | 6.78 | 0.04 | 0.29 |
| Z1458239348 | Enamine | 0.62 | -6.30 | -43.60 | -41.78 | 9.65 | -49.25 | 7.57 | -48.71 | 7.10 | -46.58 | 8.11 | 0.04 | 0.30 |
| Z1557187758 | Enamine | 0.66 | -5.64 | -43.56 | -43.09 | 8.44 | -43.34 | 8.42 | -50.53 | 6.34 | -45.65 | 7.73 | 0.04 | 0.40 |
| AP-263/43502943 | SPECS | 0.76 | -5.71 | -50.19 | -45.92 | 4.51 | -36.48 | 7.55 | -52.89 | 5.07 | -45.10 | 5.71 | 0.04 | 0.46 |
| V013-1426 | CHEMDIV | 0.52 | -5.93 | -42.69 | -46.96 | 7.09 | -54.52 | 6.60 | -32.75 | 5.86 | -44.75 | 6.51 | 0.04 | 0.50 |
| L250-1086 | CHEMDIV | 0.87 | -5.86 | -43.46 | -42.93 | 9.90 | -45.64 | 6.55 | -44.37 | 5.20 | -44.31 | 7.22 | 0.04 | 0.55 |
| AP-906/42709278 | SPECS | 0.60 | -5.45 | -35.83 | -39.87 | 5.17 | -45.00 | 5.65 | -47.58 | 9.64 | -44.15 | 6.82 | 0.04 | 0.57 |
| F864-0610 | CHEMDIV | 0.69 | -5.95 | -46.19 | -41.15 | 7.77 | -46.83 | 5.39 | -41.12 | 5.60 | -43.03 | 6.25 | 0.03 | 0.69 |
| S823-2733 | CHEMDIV | 0.72 | -5.97 | -45.92 | -58.37 | 5.35 | -41.28 | 8.11 | -28.36 | 6.22 | -42.67 | 6.56 | 0.03 | 0.73 |
| 4239-0454 | CHEMDIV | 0.65 | -5.87 | -44.81 | -35.90 | 8.62 | -39.94 | 4.78 | -51.79 | 4.37 | -42.54 | 5.92 | 0.03 | 0.75 |
| AU-059/43516942 | SPECS | 0.89 | -7.16 | -35.31 | -38.73 | 5.29 | -42.21 | 5.96 | -46.18 | 6.82 | -42.37 | 6.03 | 0.03 | 0.77 |
| Z2058595523 | Enamine | 0.51 | -5.98 | -34.24 | -34.42 | 11.32 | -43.04 | 8.07 | -49.48 | 6.19 | -42.32 | 8.52 | 0.03 | 0.77 |
| Z1139558802 | Enamine | 0.53 | -5.98 | -34.98 | -45.05 | 4.51 | -40.98 | 6.03 | -40.49 | 5.22 | -42.17 | 5.25 | 0.03 | 0.79 |
| M056-0414 | CHEMDIV | 0.76 | -5.70 | -48.73 | -37.53 | 8.73 | -39.60 | 6.63 | -45.66 | 8.49 | -40.93 | 7.95 | 0.03 | 0.93 |
| S823-7058 | CHEMDIV | 0.69 | -6.31 | -42.58 | -39.65 | 8.58 | -43.19 | 9.95 | -38.75 | 4.69 | -40.53 | 7.74 | 0.03 | 0.97 |
| Z2718693287 | Enamine | 0.53 | -5.83 | -42.08 | -40.44 | 4.78 | -36.51 | 6.24 | -37.32 | 6.51 | -38.09 | 5.84 | 0.02 | 1.24 |
| Z3687066063 | Enamine | 0.82 | -6.02 | -46.34 | -39.63 | 6.17 | -36.58 | 4.83 | -36.52 | 9.33 | -37.57 | 6.78 | 0.02 | 1.30 |
| Z2949240524 | Enamine | 0.51 | -5.54 | -38.07 | -31.75 | 11.73 | -44.89 | 5.78 | -35.34 | 6.10 | -37.33 | 7.87 | 0.02 | 1.33 |
| Z1759941366 | Enamine | 0.52 | -6.17 | -49.18 | -40.98 | 10.73 | -40.09 | 8.57 | -28.29 | 12.89 | -36.46 | 10.73 | 0.02 | 1.42 |
| Z2436226583 | Enamine | 0.63 | -5.54 | -40.21 | -39.14 | 4.70 | -28.68 | 7.81 | -40.68 | 8.86 | -36.17 | 7.12 | 0.02 | 1.45 |
| Z2752945308 | Enamine | 0.62 | -5.56 | -38.33 | -40.94 | 8.03 | -30.28 | 5.54 | -36.87 | 3.89 | -36.03 | 5.82 | 0.02 | 1.47 |
| Z4521554399 | Enamine | 0.68 | -5.73 | -39.61 | -37.81 | 4.04 | -34.92 | 6.35 | -35.07 | 7.87 | -35.93 | 6.09 | 0.01 | 1.48 |
| AU-059/02895030 | SPECS | 0.86 | -5.67 | -31.86 | -39.51 | 7.60 | -27.25 | 7.87 | -39.00 | 6.26 | -35.25 | 7.24 | 0.01 | 1.56 |
| AJ-030/14523202 | SPECS | 0.68 | -6.00 | -38.60 | -34.76 | 8.18 | -27.22 | 11.39 | -38.05 | 6.08 | -33.34 | 8.55 | 0.01 | 1.77 |
| AJ-030/12105275 | SPECS | 0.62 | -5.33 | -31.30 | -28.01 | 11.41 | -29.58 | 5.63 | -39.93 | 7.60 | -32.51 | 8.21 | 0.01 | 1.86 |
| CHEMBL3617209 | CHEMBL | 0.83 | -3.90 | -18.75 | -28.73 | 6.31 | -39.10 | 5.51 | -23.13 | 8.25 | -30.32 | 6.69 | 0.00 | 2.10 |
| AB-323/13887094 | SPECS | 0.83 | -6.39 | -40.59 | -24.17 | 8.64 | -21.95 | 9.88 | -27.24 | 4.78 | -24.45 | 7.76 | 0.00 | 2.76 |

Table S 7: The predicted anticancer activity, docking scores, post-virtual screening workflow average MM/GBSA scores, and the average MM/GBSA scores of the 3 short MD runs performed on the top 25 molecules obtained from each library in the XPF protein’s binding site 1

| **Ligand ID** | **Library** | **Anticancer Activity Prediction** | **Docking Score (kcal/mol)** | **Post-VSW MM/GBSA (kcal/mol)** | **MM/GBSA Run 1 (kcal/mol)** | **Standard Deviation Run 1** | **MM/GBSA Run 2 (kcal/mol)** | **Standard Deviation Run 2** | **MM/GBSA Run 3 (kcal/mol)** | **Standard Deviation Run 3** | **MMGBSA Average (kcal/mol)** | **Standard Deviation Average** | **Normal Distribution** | **Z-score** |
| --- | --- | --- | --- | --- | --- | --- | --- | --- | --- | --- | --- | --- | --- | --- |
| Z1780715778 | Enamine | 0.67 | -7.34 | -76.95 | -78.91 | 6.00 | -79.07 | 5.93 | -76.50 | 5.42 | -78.16 | 5.78 | 0.00 | -2.15 |
| S751-0309 | CHEMDIV | 0.76 | -7.68 | -69.28 | -79.88 | 6.90 | -84.20 | 4.43 | -68.15 | 9.49 | -77.41 | 6.94 | 0.01 | -2.07 |
| AF-399/33695064 | SPECS | 0.71 | -6.738 | -62.6 | -70.694 | 8.06 | -86.02 | 11.06 | -64.409 | 6.63 | -73.71 | 8.58 | 0.01 | -1.64 |
| S693-0564 | CHEMDIV | 0.75 | -7.26 | -69.54 | -73.36 | 5.62 | -76.00 | 6.09 | -68.66 | 5.79 | -72.67 | 5.83 | 0.01 | -1.53 |
| Z44552459 | Enamine | 0.68 | -7.27 | -62.71 | -65.05 | 6.60 | -68.85 | 8.88 | -81.47 | 6.64 | -71.79 | 7.37 | 0.02 | -1.43 |
| AN-465/42784427 | SPECS | 0.66 | -6.73 | -75.18 | -78.545 | 4.80 | -69.476 | 6.76 | -67.273 | 12.08 | -71.76 | 7.88 | 0.02 | -1.42 |
| Z32612232 | Enamine | 0.58 | -7.16 | -75.89 | -69.03 | 4.32 | -72.41 | 7.17 | -73.79 | 5.26 | -71.74 | 5.58 | 0.02 | -1.42 |
| AJ-030/14523202 | SPECS | 0.68 | -7.317 | -63.82 | -64.288 | 6.60 | -76.12 | 8.20 | -72.324 | 6.70 | -70.91 | 7.17 | 0.02 | -1.33 |
| Z223669298 | Enamine | 0.75 | -7.40 | -72.05 | -76.99 | 5.40 | -74.35 | 5.14 | -61.08 | 5.51 | -70.81 | 5.35 | 0.02 | -1.32 |
| AN-989/14207008 | SPECS | 0.76 | -7.364 | -65.9 | -70.23 | 4.92 | -69.413 | 5.39 | -67.42 | 6.13 | -69.02 | 5.48 | 0.02 | -1.11 |
| AG-205/37160013 | SPECS | 0.58 | -6.605 | -74.31 | -71.758 | 5.19 | -66.951 | 11.23 | -67.673 | 10.48 | -68.79 | 8.97 | 0.03 | -1.09 |
| AN-465/42784380 | SPECS | 0.68 | -6.62 | -68.91 | -65.872 | 5.80 | -70.026 | 7.61 | -65.271 | 7.74 | -67.06 | 7.05 | 0.03 | -0.89 |
| Y040-3226 | CHEMDIV | 0.71 | -7.26 | -51.27 | -66.38 | 7.78 | -67.70 | 6.59 | -64.00 | 5.64 | -66.03 | 6.67 | 0.03 | -0.77 |
| K809-0207 | CHEMDIV | 0.67 | -7.45 | -58.99 | -64.63 | 6.75 | -67.87 | 4.05 | -64.24 | 4.47 | -65.58 | 5.09 | 0.03 | -0.72 |
| S694-2126 | CHEMDIV | 0.60 | -7.94 | -67.45 | -69.97 | 8.69 | -65.03 | 6.10 | -61.60 | 8.67 | -65.53 | 7.82 | 0.04 | -0.72 |
| AN-465/42837318 | SPECS | 0.68 | -6.649 | -73.51 | -65.047 | 7.31 | -66.858 | 4.55 | -64.561 | 5.37 | -65.49 | 5.74 | 0.04 | -0.71 |
| 8013-5770 | CHEMDIV | 0.69 | -7.71 | -62.95 | -67.51 | 5.42 | -65.88 | 7.59 | -62.63 | 9.69 | -65.34 | 7.57 | 0.04 | -0.69 |
| AH-487/41802359 | SPECS | 0.57 | -6.684 | -79.36 | -61.003 | 9.77 | -60.35 | 7.96 | -72.563 | 6.12 | -64.64 | 7.95 | 0.04 | -0.61 |
| AG-205/33120026 | SPECS | 0.75 | -6.87 | -62.98 | -67.072 | 7.54 | -67.273 | 6.46 | -58.326 | 5.91 | -64.22 | 6.64 | 0.04 | -0.57 |
| Z53062251 | Enamine | 0.69 | -7.02 | -75.92 | -65.27 | 9.01 | -65.65 | 6.32 | -61.57 | 7.88 | -64.16 | 7.74 | 0.04 | -0.56 |
| Z1870253693 | Enamine | 0.71 | -6.93 | -51.81 | -72.70 | 6.21 | -53.18 | 4.34 | -65.66 | 6.92 | -63.85 | 5.83 | 0.04 | -0.52 |
| 8016-9938 | CHEMDIV | 0.80 | -7.66 | -63.34 | -70.52 | 6.37 | -72.24 | 9.31 | -48.64 | 15.26 | -63.80 | 10.31 | 0.04 | -0.52 |
| Z1368889688 | Enamine | 0.60 | -7.01 | -68.26 | -58.38 | 8.47 | -74.13 | 6.98 | -58.83 | 9.15 | -63.78 | 8.20 | 0.04 | -0.52 |
| C730-0387 | CHEMDIV | 0.65 | -7.32 | -63.17 | -69.04 | 8.47 | -55.65 | 7.59 | -64.69 | 4.37 | -63.13 | 6.81 | 0.04 | -0.44 |
| P163-0721 | CHEMDIV | 0.65 | -7.47 | -63.64 | -57.61 | 10.69 | -61.28 | 6.10 | -70.02 | 5.85 | -62.97 | 7.55 | 0.04 | -0.42 |
| Z1432963431 | Enamine | 0.69 | -7.31 | -54.06 | -64.48 | 5.53 | -62.58 | 7.86 | -61.65 | 7.19 | -62.90 | 6.86 | 0.04 | -0.42 |
| AQ-390/42486772 | SPECS | 0.69 | -7.253 | -55.75 | -65.05 | 6.62 | -57.973 | 6.08 | -65.186 | 4.97 | -62.74 | 5.89 | 0.04 | -0.40 |
| AE-848/42025028 | SPECS | 0.5 | -7.076 | -63.07 | -66.169 | 4.86 | -57.754 | 6.29 | -63.479 | 10.85 | -62.47 | 7.33 | 0.04 | -0.37 |
| P163-0686 | CHEMDIV | 0.67 | -7.43 | -62.49 | -65.16 | 6.41 | -61.89 | 8.58 | -60.16 | 6.39 | -62.41 | 7.13 | 0.04 | -0.36 |
| L470-1156 | CHEMDIV | 0.69 | -8.02 | -57.37 | -76.03 | 8.75 | -53.63 | 7.43 | -57.45 | 8.16 | -62.37 | 8.11 | 0.04 | -0.36 |
| AN-465/43421612 | SPECS | 0.52 | -6.681 | -68.84 | -71.746 | 4.88 | -57.313 | 5.86 | -57.977 | 5.56 | -62.35 | 5.43 | 0.04 | -0.35 |
| Z98619973 | Enamine | 0.58 | -7.15 | -74.71 | -63.58 | 4.72 | -64.25 | 4.81 | -58.90 | 7.13 | -62.24 | 5.56 | 0.04 | -0.34 |
| Y041-5407 | CHEMDIV | 0.60 | -7.60 | -64.17 | -62.21 | 4.99 | -66.24 | 6.33 | -57.75 | 9.79 | -62.07 | 7.04 | 0.04 | -0.32 |
| Z2061548941 | Enamine | 0.71 | -7.60 | -59.32 | -60.71 | 6.53 | -66.73 | 6.88 | -57.71 | 5.18 | -61.71 | 6.20 | 0.04 | -0.28 |
| AE-562/43458559 | SPECS | 0.6 | -6.682 | -65.18 | -58.475 | 5.94 | -61.848 | 4.89 | -64.686 | 8.63 | -61.67 | 6.48 | 0.04 | -0.28 |
| Z953541600 | Enamine | 0.64 | -6.92 | -62.84 | -60.15 | 5.29 | -58.01 | 5.98 | -66.21 | 5.36 | -61.45 | 5.54 | 0.04 | -0.25 |
| AO-081/13877025 | SPECS | 0.66 | -6.55 | -77.16 | -57.825 | 7.55 | -60.935 | 4.70 | -65.05 | 9.64 | -61.27 | 7.29 | 0.04 | -0.23 |
| AG-690/36722057 | SPECS | 0.51 | -7.543 | -57.12 | -56.258 | 5.90 | -60.056 | 4.39 | -66.971 | 5.47 | -61.10 | 5.25 | 0.04 | -0.21 |
| AN-329/40315446 | SPECS | 0.53 | -7.071 | -55.73 | -55.197 | 3.69 | -56.743 | 6.15 | -68.274 | 5.25 | -60.07 | 5.03 | 0.05 | -0.10 |
| AN-329/43385617 | SPECS | 0.55 | -6.779 | -59.49 | -59.119 | 4.85 | -64.009 | 7.84 | -56.352 | 6.74 | -59.83 | 6.48 | 0.05 | -0.07 |
| 8014-9180 | CHEMDIV | 0.64 | -7.40 | -60.00 | -70.44 | 8.00 | -56.40 | 8.74 | -51.42 | 7.23 | -59.42 | 7.99 | 0.05 | -0.02 |
| Z1014134436 | Enamine | 0.65 | -7.08 | -69.49 | -66.71 | 7.89 | -54.07 | 9.25 | -57.48 | 7.36 | -59.42 | 8.17 | 0.05 | -0.02 |
| AG-690/11629600 | SPECS | 0.74 | -7.283 | -59.92 | -60.156 | 4.66 | -57.086 | 6.30 | -61.001 | 3.48 | -59.41 | 4.81 | 0.05 | -0.02 |
| SC97-0091 | CHEMDIV | 0.64 | -7.47 | -68.79 | -61.45 | 7.01 | -51.38 | 6.39 | -65.17 | 6.30 | -59.34 | 6.57 | 0.05 | -0.01 |
| Z979050590 | Enamine | 0.55 | -6.99 | -71.85 | -63.78 | 7.85 | -65.38 | 6.79 | -48.62 | 9.29 | -59.26 | 7.98 | 0.05 | 0.00 |
| 8018-2012 | CHEMDIV | 0.58 | -7.72 | -58.24 | -57.03 | 9.13 | -59.84 | 6.76 | -53.94 | 5.77 | -56.93 | 7.22 | 0.04 | 0.26 |
| AN-038/12879021 | SPECS | 0.5 | -6.61 | -62.55 | -59.347 | 8.52 | -55.879 | 4.74 | -54.845 | 6.74 | -56.69 | 6.67 | 0.04 | 0.29 |
| S695-1993 | CHEMDIV | 0.71 | -8.39 | -70.87 | -58.18 | 7.11 | -54.64 | 9.19 | -56.14 | 8.04 | -56.32 | 8.11 | 0.04 | 0.33 |
| Z448395586 | Enamine | 0.65 | -7.24 | -59.56 | -55.45 | 4.24 | -54.84 | 7.28 | -55.20 | 5.23 | -55.16 | 5.59 | 0.04 | 0.46 |
| C730-0097 | CHEMDIV | 0.60 | -7.71 | -52.48 | -61.95 | 5.18 | -51.56 | 8.57 | -51.55 | 4.95 | -55.02 | 6.23 | 0.04 | 0.48 |
| Z1400862286 | Enamine | 0.61 | -7.58 | -55.76 | -45.74 | 10.16 | -57.22 | 4.98 | -60.51 | 4.95 | -54.49 | 6.70 | 0.04 | 0.54 |
| Z1259180721 | Enamine | 0.71 | -7.44 | -50.89 | -58.10 | 4.77 | -50.99 | 5.92 | -53.93 | 4.35 | -54.34 | 5.01 | 0.04 | 0.56 |
| C730-0194 | CHEMDIV | 0.80 | -7.30 | -56.82 | -45.30 | 7.53 | -50.13 | 4.60 | -67.43 | 6.64 | -54.29 | 6.26 | 0.04 | 0.56 |
| G745-0718 | CHEMDIV | 0.65 | -7.70 | -64.12 | -57.88 | 7.48 | -53.32 | 6.63 | -51.48 | 8.67 | -54.23 | 7.59 | 0.04 | 0.57 |
| Z1445214197 | Enamine | 0.57 | -6.92 | -61.03 | -49.86 | 4.14 | -54.85 | 9.30 | -55.39 | 6.80 | -53.36 | 6.75 | 0.04 | 0.67 |
| CHEMBL3617209 | CHEMBL | 0.83 | -4.19 | -43.98 | -51.60 | 4.91 | -50.23 | 4.77 | -56.74 | 6.46 | -52.86 | 5.38 | 0.03 | 0.72 |
| Z1208157951 | Enamine | 0.76 | -7.14 | -58.93 | -54.68 | 5.42 | -48.60 | 5.87 | -54.61 | 4.11 | -52.63 | 5.13 | 0.03 | 0.75 |
| 8015-7688 | CHEMDIV | 0.59 | -7.41 | -56.32 | -52.93 | 5.18 | -49.20 | 5.14 | -55.56 | 5.59 | -52.56 | 5.30 | 0.03 | 0.76 |
| S564-0206 | CHEMDIV | 0.71 | -7.39 | -65.77 | -56.59 | 5.79 | -54.30 | 6.76 | -46.07 | 8.86 | -52.32 | 7.14 | 0.03 | 0.78 |
| 8016-9939 | CHEMDIV | 0.66 | -7.80 | -61.44 | -54.71 | 7.60 | -49.22 | 8.63 | -52.82 | 5.20 | -52.25 | 7.14 | 0.03 | 0.79 |
| AK-968/11532066 | SPECS | 0.57 | -7.182 | -55.08 | -52.123 | 5.08 | -59.225 | 5.01 | -45.224 | 7.10 | -52.19 | 5.73 | 0.03 | 0.80 |
| AM-879/15551036 | SPECS | 0.8 | -6.821 | -55.2 | -54 | 5.09 | -46.514 | 6.64 | -55.682 | 5.66 | -52.07 | 5.79 | 0.03 | 0.81 |
| Z2848694734 | Enamine | 0.50 | -7.38 | -63.79 | -51.55 | 4.02 | -55.33 | 4.79 | -49.09 | 7.54 | -51.99 | 5.45 | 0.03 | 0.82 |
| Z2177387705 | Enamine | 0.58 | -7.03 | -62.79 | -55.64 | 6.98 | -55.19 | 5.88 | -45.05 | 8.08 | -51.96 | 6.98 | 0.03 | 0.83 |
| Z1730024826 | Enamine | 0.88 | -7.01 | -54.24 | -53.57 | 6.71 | -50.64 | 8.61 | -51.05 | 9.54 | -51.75 | 8.28 | 0.03 | 0.85 |
| 8017-8335 | CHEMDIV | 0.68 | -7.87 | -60.39 | -40.79 | 7.39 | -49.88 | 5.87 | -64.33 | 5.44 | -51.66 | 6.23 | 0.03 | 0.86 |
| 8018-3138 | CHEMDIV | 0.74 | -7.32 | -50.29 | -47.09 | 10.77 | -57.93 | 6.96 | -47.90 | 6.80 | -50.97 | 8.18 | 0.03 | 0.94 |
| Z1397034446 | Enamine | 0.71 | -6.96 | -58.24 | -52.24 | 5.87 | -46.68 | 5.92 | -53.64 | 3.26 | -50.85 | 5.02 | 0.03 | 0.95 |
| C730-0096 | CHEMDIV | 0.70 | -7.63 | -54.60 | -50.93 | 5.94 | -43.39 | 8.69 | -57.72 | 7.82 | -50.68 | 7.49 | 0.03 | 0.97 |
| AK-968/15363910 | SPECS | 0.56 | -7.005 | -50.32 | -48.809 | 8.80 | -48.935 | 6.44 | -50.521 | 7.85 | -49.42 | 7.70 | 0.02 | 1.11 |
| Z763434512 | Enamine | 0.64 | -7.33 | -51.48 | -49.22 | 2.58 | -41.49 | 5.61 | -49.76 | 4.44 | -46.82 | 4.21 | 0.02 | 1.41 |
| AK-968/41025827 | SPECS | 0.51 | -6.736 | -46.92 | -47.578 | 5.12 | -45.281 | 6.59 | -47.103 | 4.88 | -46.65 | 5.53 | 0.02 | 1.43 |
| Z927307644 | Enamine | 0.70 | -7.07 | -60.38 | -53.41 | 3.44 | -52.46 | 4.66 | -32.65 | 13.04 | -46.17 | 7.04 | 0.02 | 1.48 |
| Z786105464 | Enamine | 0.74 | -6.92 | -55.89 | -39.31 | 8.83 | -55.35 | 6.53 | -41.17 | 8.07 | -45.28 | 7.81 | 0.01 | 1.58 |
| AB-323/13887094 | SPECS | 0.83 | -6.955 | -59.77 | -51.402 | 6.48 | -43.729 | 6.16 | -34.78 | 10.87 | -43.30 | 7.84 | 0.01 | 1.81 |
| AR-422/41337951 | SPECS | 0.66 | -6.639 | -38.13 | -31.14 | 4.74 | -32.871 | 3.88 | -29.659 | 4.61 | -31.22 | 4.41 | 0.00 | 3.18 |

Table S 8: The predicted anticancer activity, docking scores, post-virtual screening workflow average MM/GBSA scores, and the average MM/GBSA scores of the 3 short MD runs performed on the top 25 molecules obtained from each library in the XPF protein’s binding site 2

| **Ligand ID** | **Library** | **Anticancer Activity Prediction** | **Docking Score (kcal/mol)** | **Post-VSW MM/GBSA (kcal/mol)** | **MM/GBSA Run 1 (kcal/mol)** | **Standard Deviation Run 1** | **MM/GBSA Run 2 (kcal/mol)** | **Standard Deviation Run 2** | **MM/GBSA Run 3 (kcal/mol)** | **Standard Deviation Run 3** | **MMGBSA Average (kcal/mol)** | **Standard Deviation Average** | **Normal Distribution** | **Z-score** |
| --- | --- | --- | --- | --- | --- | --- | --- | --- | --- | --- | --- | --- | --- | --- |
| F818-0233 | CHEMDIV | 0.76 | -5.35 | -50.98 | -60.34 | 8.64 | -60.11 | 6.56 | -48.59 | 5.26 | -56.34 | 6.82 | 0.00 | -2.12 |
| Y020-2805 | CHEMDIV | 0.70 | -5.72 | -50.37 | -48.49 | 4.99 | -58.11 | 6.05 | -59.22 | 6.87 | -55.27 | 5.97 | 0.01 | -2.01 |
| Z605272712 | Enamine | 0.70 | -5.88 | -41.79 | -53.09 | 9.00 | -53.14 | 5.90 | -59.30 | 6.76 | -55.18 | 7.22 | 0.01 | -2.00 |
| AP-970/42837256 | SPECS | 0.70 | -4.49 | -46.51 | -52.92 | 5.44 | -49.89 | 5.65 | -45.43 | 5.80 | -49.42 | 5.63 | 0.01 | -1.42 |
| AP-124/43382853 | SPECS | 0.60 | -4.34 | -38.57 | -54.83 | 10.56 | -39.51 | 6.59 | -51.27 | 9.03 | -48.53 | 8.73 | 0.02 | -1.33 |
| 8020-2489 | CHEMDIV | 0.65 | -5.04 | -44.85 | -49.79 | 6.09 | -39.06 | 7.05 | -55.51 | 6.88 | -48.12 | 6.67 | 0.02 | -1.29 |
| AG-690/11426248 | SPECS | 0.67 | -4.76 | -48.23 | -42.75 | 7.72 | -49.93 | 9.13 | -50.51 | 5.46 | -47.73 | 7.44 | 0.02 | -1.25 |
| AQ-390/10770038 | SPECS | 0.58 | -5.59 | -50.38 | -49.34 | 6.30 | -47.83 | 5.34 | -45.73 | 5.22 | -47.63 | 5.62 | 0.02 | -1.24 |
| 8014-2464 | CHEMDIV | 0.85 | -5.07 | -43.79 | -48.08 | 6.62 | -47.19 | 7.15 | -44.52 | 5.42 | -46.60 | 6.40 | 0.02 | -1.13 |
| J001-0937 | CHEMDIV | 0.64 | -5.10 | -41.36 | -48.31 | 5.86 | -41.52 | 8.25 | -48.89 | 6.89 | -46.24 | 7.00 | 0.02 | -1.10 |
| 8018-6778 | CHEMDIV | 0.63 | -5.05 | -42.99 | -45.29 | 4.41 | -46.75 | 5.09 | -45.87 | 4.34 | -45.97 | 4.61 | 0.02 | -1.07 |
| 8018-4534 | CHEMDIV | 0.67 | -5.07 | -41.09 | -51.35 | 6.81 | -46.42 | 5.05 | -39.62 | 3.52 | -45.80 | 5.13 | 0.02 | -1.05 |
| 8020-3757 | CHEMDIV | 0.66 | -5.91 | -46.51 | -42.04 | 7.06 | -59.28 | 9.43 | -33.81 | 7.04 | -45.04 | 7.84 | 0.03 | -0.97 |
| AQ-390/10769005 | SPECS | 0.56 | -6.06 | -48.16 | -48.04 | 5.59 | -49.47 | 5.42 | -36.60 | 5.62 | -44.71 | 5.54 | 0.03 | -0.94 |
| Z851136910 | Enamine | 0.84 | -5.21 | -38.46 | -45.85 | 8.15 | -37.78 | 7.89 | -49.79 | 6.58 | -44.47 | 7.54 | 0.03 | -0.92 |
| AO-567/41986932 | SPECS | 0.67 | -5.35 | -41.65 | -45.80 | 4.50 | -42.28 | 5.90 | -44.79 | 4.91 | -44.29 | 5.10 | 0.03 | -0.90 |
| F818-0061 | CHEMDIV | 0.85 | -5.53 | -46.43 | -44.60 | 4.47 | -49.41 | 6.70 | -34.62 | 6.11 | -42.88 | 5.76 | 0.03 | -0.76 |
| Z1730024826 | Enamine | 0.88 | -5.36 | -42.27 | -43.60 | 4.75 | -38.29 | 3.90 | -46.09 | 7.07 | -42.66 | 5.24 | 0.03 | -0.73 |
| AN-465/15537065 | SPECS | 0.63 | -4.29 | -23.69 | -38.10 | 7.72 | -46.45 | 8.68 | -42.74 | 7.78 | -42.43 | 8.06 | 0.03 | -0.71 |
| Z2234066329 | Enamine | 0.77 | -5.50 | -46.02 | -39.44 | 6.40 | -41.20 | 5.82 | -46.53 | 6.18 | -42.39 | 6.13 | 0.03 | -0.71 |
| 8013-0081 | CHEMDIV | 0.53 | -4.98 | -44.25 | -49.48 | 4.95 | -50.85 | 6.63 | -25.14 | 7.56 | -41.82 | 6.38 | 0.03 | -0.65 |
| AS-871/42707293 | SPECS | 0.82 | -4.68 | -43.12 | -40.62 | 5.37 | -44.69 | 6.41 | -39.72 | 7.96 | -41.67 | 6.58 | 0.03 | -0.63 |
| AP-263/09761017 | SPECS | 0.55 | -4.35 | -48.89 | -39.76 | 4.96 | -46.83 | 6.77 | -38.21 | 6.55 | -41.60 | 6.09 | 0.03 | -0.63 |
| Y020-0895 | CHEMDIV | 0.82 | -5.18 | -36.28 | -48.87 | 7.42 | -41.04 | 7.41 | -34.37 | 8.55 | -41.43 | 7.79 | 0.03 | -0.61 |
| Z851136976 | Enamine | 0.89 | -4.97 | -39.70 | -42.74 | 5.59 | -40.26 | 6.38 | -39.10 | 4.92 | -40.70 | 5.63 | 0.04 | -0.53 |
| Z1368903771 | Enamine | 0.61 | -6.19 | -35.78 | -46.00 | 5.53 | -37.39 | 3.45 | -38.10 | 4.83 | -40.49 | 4.61 | 0.04 | -0.51 |
| D399-0519 | CHEMDIV | 0.69 | -5.59 | -36.80 | -35.30 | 7.66 | -46.48 | 7.62 | -39.59 | 6.25 | -40.46 | 7.18 | 0.04 | -0.51 |
| 8014-7125 | CHEMDIV | 0.59 | -6.01 | -47.30 | -44.57 | 6.31 | -37.51 | 10.71 | -37.09 | 6.62 | -39.72 | 7.88 | 0.04 | -0.44 |
| C143-0022 | CHEMDIV | 0.78 | -5.41 | -33.93 | -37.11 | 4.23 | -37.78 | 4.41 | -44.06 | 6.65 | -39.65 | 5.10 | 0.04 | -0.43 |
| Z2761811684 | Enamine | 0.65 | -5.36 | -38.60 | -39.17 | 7.14 | -35.04 | 5.13 | -44.50 | 7.83 | -39.57 | 6.70 | 0.04 | -0.42 |
| C224-0537 | CHEMDIV | 0.51 | -5.96 | -48.49 | -45.93 | 6.94 | -45.75 | 8.10 | -26.74 | 9.05 | -39.47 | 8.03 | 0.04 | -0.41 |
| AH-487/41733581 | SPECS | 0.60 | -4.27 | -33.20 | -40.17 | 5.30 | -40.05 | 6.76 | -38.14 | 6.91 | -39.45 | 6.32 | 0.04 | -0.41 |
| Z806732044 | Enamine | 0.68 | -5.79 | -41.38 | -43.24 | 5.58 | -37.75 | 4.59 | -35.82 | 6.66 | -38.93 | 5.61 | 0.04 | -0.36 |
| Z1231798086 | Enamine | 0.82 | -5.24 | -39.91 | -45.78 | 6.75 | -29.65 | 5.72 | -39.70 | 8.19 | -38.38 | 6.89 | 0.04 | -0.30 |
| Z2798534309 | Enamine | 0.75 | -5.54 | -37.01 | -36.92 | 5.95 | -33.46 | 5.90 | -43.92 | 4.49 | -38.10 | 5.45 | 0.04 | -0.27 |
| AN-465/43411388 | SPECS | 0.69 | -4.69 | -36.42 | -41.55 | 4.94 | -29.59 | 5.60 | -41.11 | 5.56 | -37.41 | 5.36 | 0.04 | -0.20 |
| S574-0050 | CHEMDIV | 0.74 | -5.03 | -40.36 | -42.68 | 5.63 | -33.04 | 4.64 | -36.37 | 6.00 | -37.36 | 5.42 | 0.04 | -0.20 |
| AK-968/41017880 | SPECS | 0.72 | -4.59 | -40.32 | -48.87 | 8.76 | -31.64 | 5.27 | -30.33 | 5.23 | -36.94 | 6.42 | 0.04 | -0.15 |
| AN-465/43460906 | SPECS | 0.70 | -5.01 | -31.19 | -34.11 | 8.34 | -39.09 | 7.80 | -36.69 | 5.06 | -36.63 | 7.06 | 0.04 | -0.12 |
| Z3141417228 | Enamine | 0.66 | -5.26 | -34.48 | -39.62 | 5.08 | -29.79 | 4.81 | -39.13 | 4.42 | -36.18 | 4.77 | 0.04 | -0.08 |
| Z1670866678 | Enamine | 0.61 | -4.84 | -33.58 | -37.67 | 11.15 | -33.30 | 6.67 | -36.90 | 8.33 | -35.96 | 8.72 | 0.04 | -0.05 |
| AN-465/41672854 | SPECS | 0.51 | -4.27 | -45.88 | -31.62 | 7.53 | -33.66 | 8.11 | -41.40 | 6.53 | -35.56 | 7.39 | 0.04 | -0.01 |
| 3332-4017 | CHEMDIV | 0.68 | -5.16 | -43.43 | -39.09 | 6.51 | -33.99 | 4.92 | -32.85 | 6.19 | -35.31 | 5.88 | 0.04 | 0.01 |
| Z441455848 | Enamine | 0.77 | -5.02 | -35.69 | -31.18 | 6.03 | -30.75 | 5.85 | -43.23 | 4.46 | -35.05 | 5.45 | 0.04 | 0.04 |
| Z1385552890 | Enamine | 0.54 | -4.86 | -47.86 | -33.67 | 7.27 | -26.79 | 15.93 | -42.52 | 5.45 | -34.33 | 9.55 | 0.04 | 0.11 |
| 8563-0830 | CHEMDIV | 0.72 | -5.04 | -42.91 | -39.06 | 7.67 | -35.44 | 5.69 | -22.34 | 10.91 | -32.28 | 8.09 | 0.04 | 0.32 |
| C200-9369 | CHEMDIV | 0.73 | -5.61 | -39.71 | -40.67 | 5.17 | -36.60 | 7.10 | -16.73 | 10.24 | -31.33 | 7.50 | 0.04 | 0.41 |
| AH-034/12055535 | SPECS | 0.67 | -4.36 | -20.70 | -42.44 | 7.88 | -23.92 | 4.28 | -27.54 | 8.08 | -31.30 | 6.75 | 0.04 | 0.42 |
| Z227966836 | Enamine | 0.81 | -5.16 | -21.64 | -29.88 | 7.75 | -37.22 | 12.27 | -25.36 | 3.76 | -30.82 | 7.93 | 0.04 | 0.47 |
| 8013-1758 | CHEMDIV | 0.79 | -5.25 | -38.35 | -28.92 | 8.47 | -31.40 | 3.19 | -30.76 | 6.11 | -30.36 | 5.92 | 0.04 | 0.51 |
| AB-323/13887107 | SPECS | 0.66 | -5.00 | -39.16 | -35.43 | 5.16 | -27.58 | 5.77 | -27.01 | 6.60 | -30.00 | 5.85 | 0.03 | 0.55 |
| AQ-149/43100460 | SPECS | 0.50 | -4.73 | -24.72 | -12.69 | 8.63 | -41.53 | 7.06 | -35.52 | 6.64 | -29.92 | 7.44 | 0.03 | 0.56 |
| AR-299/42656355 | SPECS | 0.74 | -5.77 | -37.10 | -39.69 | 7.98 | -15.65 | 13.83 | -33.23 | 6.25 | -29.52 | 9.35 | 0.03 | 0.60 |
| Z968561690 | Enamine | 0.80 | -5.20 | -36.16 | -21.24 | 5.39 | -28.73 | 5.47 | -35.69 | 4.44 | -28.55 | 5.10 | 0.03 | 0.69 |
| Z2147835526 | Enamine | 0.66 | -4.98 | -22.57 | -27.45 | 6.08 | -28.80 | 5.25 | -27.44 | 6.67 | -27.90 | 6.00 | 0.03 | 0.76 |
| Z2279068644 | Enamine | 0.54 | -5.02 | -29.93 | -37.68 | 5.13 | -25.65 | 5.40 | -19.29 | 12.59 | -27.54 | 7.71 | 0.03 | 0.80 |
| Z3016339205 | Enamine | 0.52 | -5.20 | -30.65 | -20.51 | 11.15 | -31.51 | 5.46 | -30.38 | 8.83 | -27.47 | 8.48 | 0.03 | 0.80 |
| Y200-5748 | CHEMDIV | 0.62 | -5.26 | -24.96 | -32.60 | 8.08 | -22.85 | 4.15 | -25.76 | 4.89 | -27.07 | 5.71 | 0.03 | 0.85 |
| C211-0065 | CHEMDIV | 0.69 | -5.29 | -23.07 | -25.23 | 4.82 | -29.18 | 6.69 | -22.50 | 8.47 | -25.64 | 6.66 | 0.02 | 0.99 |
| AP-906/42698498 | SPECS | 0.51 | -5.41 | -24.26 | -25.94 | 4.47 | -32.02 | 9.74 | -16.54 | 6.29 | -24.83 | 6.83 | 0.02 | 1.07 |
| Z3056901989 | Enamine | 0.67 | -5.28 | -39.90 | -29.40 | 4.89 | -20.17 | 6.35 | -24.67 | 5.88 | -24.75 | 5.71 | 0.02 | 1.08 |
| Z1603490185 | Enamine | 0.75 | -5.13 | -25.06 | -30.01 | 6.74 | -17.82 | 5.30 | -26.06 | 6.50 | -24.63 | 6.18 | 0.02 | 1.09 |
| 0527-0155 | CHEMDIV | 0.72 | -5.60 | -30.93 | -7.77 | 9.57 | -34.97 | 6.98 | -30.12 | 6.38 | -24.29 | 7.64 | 0.02 | 1.13 |
| Z2149357454 | Enamine | 0.53 | -4.87 | -34.80 | -37.39 | 6.95 | -14.29 | 10.87 | -20.88 | 5.54 | -24.19 | 7.79 | 0.02 | 1.14 |
| Z608598956 | Enamine | 0.58 | -4.87 | -32.42 | -9.99 | 9.05 | -27.04 | 5.92 | -34.44 | 6.25 | -23.82 | 7.07 | 0.02 | 1.17 |
| Z1983688267 | Enamine | 0.83 | -5.66 | -27.11 | -34.94 | 7.78 | -13.96 | 12.60 | -20.96 | 7.11 | -23.29 | 9.16 | 0.02 | 1.23 |
| AM-900/14299001 | SPECS | 0.55 | -4.51 | -18.27 | -27.08 | 3.27 | -24.67 | 5.69 | -17.61 | 5.83 | -23.12 | 4.93 | 0.02 | 1.24 |
| AB-323/13887094 | SPECS | 0.83 | -4.83 | -33.13 | -20.38 | 5.87 | -26.50 | 5.18 | -21.16 | 7.38 | -22.68 | 6.14 | 0.02 | 1.29 |
| D668-0239 | CHEMDIV | 0.68 | -5.20 | -24.72 | -17.04 | 7.77 | -22.13 | 5.21 | -28.74 | 10.75 | -22.64 | 7.91 | 0.02 | 1.29 |
| AU-059/02895030 | SPECS | 0.86 | -4.54 | -27.67 | -10.92 | 8.20 | -26.37 | 7.72 | -28.04 | 6.01 | -21.78 | 7.31 | 0.02 | 1.38 |
| AG-690/33038031 | SPECS | 0.89 | -4.43 | -21.42 | -22.17 | 5.62 | -23.08 | 4.69 | -15.16 | 8.10 | -20.14 | 6.13 | 0.01 | 1.55 |
| AS-813/43501638 | SPECS | 0.86 | -4.58 | -22.98 | -18.41 | 5.63 | -19.54 | 6.86 | -21.44 | 8.35 | -19.80 | 6.95 | 0.01 | 1.58 |
| AH-487/13096054 | SPECS | 0.58 | -4.71 | -20.23 | -17.31 | 3.83 | -15.19 | 9.21 | -19.52 | 7.03 | -17.34 | 6.69 | 0.01 | 1.83 |
| CHEMBL3617209 | CHEMBL | 0.83 | -3.00 | -28.61 | -37.69 | 7.49 | -27.95 | 7.79 | -36.29 | 6.85 | -33.98 | 7.38 | 0.04 | 0.15 |
| 8019-7921 | CHEMDIV | 0.59 | -5.04 | -21.52 | -11.25 | 7.34 | -13.84 | 8.76 | -18.50 | 6.68 | -14.53 | 7.59 | 0.00 | 2.11 |
| Z2442352299 | Enamine | 0.69 | -5.43 | -24.82 | -23.40 | 5.42 | -3.85 | 8.43 | -9.40 | 9.52 | -12.22 | 7.79 | 0.00 | 2.35 |

Table S 9: The predicted anticancer activity, docking scores, post-virtual screening workflow average MM/GBSA scores, and the average MM/GBSA scores of the 3 short MD runs performed on the top 25 molecules obtained from each library in the XPF protein’s binding site 3

| **Ligand ID** | **Library** | **Anticancer Activity Prediction** | **Docking Score (kcal/mol)** | **Post-VSW MM/GBSA (kcal/mol)** | **MM/GBSA Run 1 (kcal/mol)** | **Standard Deviation Run 1** | **MM/GBSA Run 2 (kcal/mol)** | **Standard Deviation Run 2** | **MM/GBSA Run 3 (kcal/mol)** | **Standard Deviation Run 3** | **MMGBSA Average (kcal/mol)** | **Standard Deviation Average** | **Normal Distribution** | **Z-score** |
| --- | --- | --- | --- | --- | --- | --- | --- | --- | --- | --- | --- | --- | --- | --- |
| AG-205/32457039 | SPECS | 0.71 | -7.58 | -81.65 | -89.91 | 5.66 | -86.13 | 12.13 | -87.47 | 5.49 | -87.84 | 7.76 | 0.00 | -2.19 |
| AN-988/40787604 | SPECS | 0.70 | -7.51 | -82.64 | -86.42 | 6.49 | -93.97 | 10.56 | -79.40 | 7.30 | -86.60 | 8.12 | 0.00 | -2.08 |
| Y500-0018 | CHEMDIV | 0.71 | -7.82 | -81.60 | -77.81 | 6.77 | -82.02 | 6.45 | -87.61 | 6.32 | -82.48 | 6.51 | 0.01 | -1.73 |
| AH-487/40936989 | SPECS | 0.53 | -7.09 | -76.90 | -100.64 | 11.80 | -69.61 | 5.71 | -73.16 | 8.48 | -81.14 | 8.66 | 0.01 | -1.61 |
| AK-968/13035103 | SPECS | 0.61 | -7.04 | -73.30 | -87.61 | 8.04 | -77.02 | 8.51 | -78.01 | 6.65 | -80.88 | 7.73 | 0.01 | -1.59 |
| K264-0192 | CHEMDIV | 0.59 | -7.37 | -83.54 | -77.61 | 8.00 | -81.27 | 5.22 | -81.23 | 7.63 | -80.04 | 6.95 | 0.01 | -1.52 |
| AO-476/42169428 | SPECS | 0.70 | -7.69 | -64.48 | -80.03 | 9.74 | -76.49 | 5.58 | -81.73 | 12.95 | -79.42 | 9.42 | 0.01 | -1.46 |
| V005-2543 | CHEMDIV | 0.69 | -7.67 | -69.72 | -75.14 | 6.60 | -84.29 | 9.27 | -76.56 | 4.75 | -78.67 | 6.87 | 0.01 | -1.40 |
| AH-487/40937032 | SPECS | 0.51 | -8.05 | -81.98 | -72.47 | 7.46 | -81.60 | 6.98 | -78.55 | 8.38 | -77.54 | 7.60 | 0.01 | -1.30 |
| AN-329/40366520 | SPECS | 0.71 | -8.16 | -90.51 | -68.83 | 10.82 | -88.66 | 7.42 | -75.09 | 15.28 | -77.53 | 11.17 | 0.01 | -1.30 |
| C791-0768 | CHEMDIV | 0.70 | -7.51 | -70.07 | -74.87 | 5.34 | -71.09 | 5.73 | -84.38 | 8.61 | -76.78 | 6.56 | 0.02 | -1.24 |
| K784-8026 | CHEMDIV | 0.75 | -8.98 | -77.11 | -70.37 | 12.01 | -74.55 | 7.21 | -84.50 | 11.42 | -76.47 | 10.21 | 0.02 | -1.21 |
| L780-0154 | CHEMDIV | 0.58 | -7.53 | -72.87 | -73.77 | 6.72 | -78.16 | 7.36 | -75.49 | 5.16 | -75.80 | 6.41 | 0.02 | -1.15 |
| K284-4652 | CHEMDIV | 0.68 | -7.40 | -74.48 | -74.68 | 7.01 | -81.27 | 8.65 | -70.06 | 6.99 | -75.34 | 7.55 | 0.02 | -1.11 |
| AO-476/40672162 | SPECS | 0.64 | -7.25 | -71.23 | -69.61 | 6.73 | -75.75 | 7.35 | -79.17 | 8.88 | -74.84 | 7.65 | 0.02 | -1.07 |
| K784-7930 | CHEMDIV | 0.64 | -8.50 | -74.46 | -85.89 | 5.02 | -68.22 | 9.36 | -69.80 | 9.79 | -74.64 | 8.06 | 0.02 | -1.05 |
| V029-3266 | CHEMDIV | 0.67 | -8.06 | -81.27 | -68.63 | 9.11 | -72.90 | 5.26 | -80.28 | 6.61 | -73.94 | 6.99 | 0.02 | -0.99 |
| AO-081/40926667 | SPECS | 0.50 | -7.67 | -68.46 | -74.65 | 7.50 | -69.72 | 5.58 | -73.23 | 9.10 | -72.53 | 7.39 | 0.02 | -0.87 |
| Z436695436 | Enamine | 0.86 | -7.11 | -58.73 | -72.88 | 5.14 | -72.84 | 4.16 | -68.72 | 4.68 | -71.48 | 4.66 | 0.03 | -0.78 |
| AJ-292/41694654 | SPECS | 0.61 | -7.35 | -61.98 | -63.25 | 7.80 | -73.46 | 7.79 | -77.59 | 12.03 | -71.44 | 9.21 | 0.03 | -0.78 |
| AK-968/15603857 | SPECS | 0.64 | -7.00 | -57.73 | -61.52 | 7.49 | -70.94 | 6.22 | -80.50 | 9.59 | -70.99 | 7.77 | 0.03 | -0.74 |
| V028-5882 | CHEMDIV | 0.69 | -7.86 | -68.12 | -69.45 | 4.44 | -65.56 | 5.35 | -70.79 | 5.46 | -68.60 | 5.08 | 0.03 | -0.54 |
| G117-0449 | CHEMDIV | 0.70 | -7.40 | -62.96 | -69.74 | 6.86 | -66.96 | 6.31 | -65.23 | 8.50 | -67.31 | 7.22 | 0.03 | -0.42 |
| AK-968/15359228 | SPECS | 0.73 | -6.97 | -55.34 | -69.73 | 6.86 | -62.21 | 4.52 | -69.92 | 7.16 | -67.29 | 6.18 | 0.03 | -0.42 |
| G566-1099 | CHEMDIV | 0.63 | -7.70 | -54.14 | -59.53 | 10.53 | -69.78 | 7.85 | -69.70 | 6.14 | -66.34 | 8.17 | 0.03 | -0.34 |
| AE-848/11147686 | SPECS | 0.69 | -7.11 | -71.22 | -69.68 | 5.62 | -69.81 | 6.20 | -58.27 | 6.04 | -65.92 | 5.95 | 0.03 | -0.31 |
| AO-080/43378377 | SPECS | 0.51 | -8.24 | -61.71 | -68.16 | 5.06 | -65.43 | 5.55 | -62.49 | 5.19 | -65.36 | 5.27 | 0.03 | -0.26 |
| Z1815548201 | Enamine | 0.71 | -7.32 | -62.03 | -60.02 | 4.58 | -68.21 | 9.40 | -67.58 | 6.04 | -65.27 | 6.67 | 0.03 | -0.25 |
| AG-667/14764021 | SPECS | 0.71 | -6.99 | -68.12 | -65.54 | 6.65 | -65.02 | 6.28 | -64.00 | 7.41 | -64.86 | 6.78 | 0.03 | -0.21 |
| AF-399/41813307 | SPECS | 0.79 | -6.97 | -60.38 | -67.39 | 6.62 | -62.44 | 7.72 | -64.35 | 6.22 | -64.73 | 6.85 | 0.03 | -0.20 |
| Z1417633398 | Enamine | 0.66 | -7.27 | -60.99 | -63.87 | 5.58 | -63.75 | 7.84 | -64.75 | 4.32 | -64.12 | 5.92 | 0.03 | -0.15 |
| AG-690/15430988 | SPECS | 0.59 | -7.25 | -60.09 | -62.43 | 5.15 | -61.47 | 6.03 | -66.29 | 5.71 | -63.40 | 5.63 | 0.03 | -0.09 |
| Z96925162 | Enamine | 0.54 | -7.15 | -66.51 | -61.79 | 5.45 | -66.26 | 7.47 | -61.95 | 4.92 | -63.33 | 5.95 | 0.03 | -0.08 |
| AO-022/43514109 | SPECS | 0.61 | -7.86 | -58.18 | -57.87 | 6.07 | -71.25 | 9.61 | -60.45 | 8.90 | -63.19 | 8.19 | 0.03 | -0.07 |
| Z18519788 | Enamine | 0.65 | -7.39 | -70.65 | -63.60 | 9.24 | -67.74 | 11.16 | -57.61 | 7.23 | -62.98 | 9.21 | 0.03 | -0.05 |
| Z31792170 | Enamine | 0.64 | -7.44 | -64.15 | -62.94 | 6.29 | -65.11 | 7.33 | -60.89 | 6.22 | -62.98 | 6.61 | 0.03 | -0.05 |
| Z1230258891 | Enamine | 0.80 | -7.10 | -61.93 | -61.90 | 6.13 | -59.51 | 6.53 | -67.06 | 13.64 | -62.82 | 8.77 | 0.03 | -0.04 |
| Z2711367105 | Enamine | 0.63 | -7.36 | -57.59 | -64.50 | 5.51 | -61.32 | 6.20 | -62.30 | 5.70 | -62.70 | 5.80 | 0.03 | -0.03 |
| AO-476/43250154 | SPECS | 0.66 | -6.98 | -64.01 | -63.39 | 13.12 | -54.32 | 9.56 | -67.44 | 10.59 | -61.72 | 11.09 | 0.03 | 0.06 |
| 8016-3092 | CHEMDIV | 0.51 | -8.12 | -58.08 | -69.36 | 5.06 | -42.09 | 8.75 | -73.00 | 4.16 | -61.49 | 5.99 | 0.03 | 0.08 |
| Z1172157111 | Enamine | 0.61 | -7.12 | -59.42 | -57.50 | 16.01 | -59.36 | 8.27 | -67.53 | 5.84 | -61.46 | 10.04 | 0.03 | 0.08 |
| G715-1380 | CHEMDIV | 0.78 | -7.88 | -64.44 | -56.31 | 6.22 | -64.46 | 3.41 | -59.91 | 5.72 | -60.23 | 5.12 | 0.03 | 0.18 |
| AG-205/36494057 | SPECS | 0.67 | -7.17 | -67.50 | -59.38 | 6.22 | -56.08 | 6.44 | -63.81 | 7.98 | -59.76 | 6.88 | 0.03 | 0.22 |
| Z32268864 | Enamine | 0.85 | -7.19 | -59.86 | -59.11 | 3.90 | -64.36 | 6.07 | -55.40 | 4.39 | -59.62 | 4.78 | 0.03 | 0.24 |
| Z370543180 | Enamine | 0.61 | -7.30 | -58.61 | -56.36 | 8.51 | -56.17 | 8.47 | -66.23 | 6.93 | -59.59 | 7.97 | 0.03 | 0.24 |
| D233-0675 | CHEMDIV | 0.80 | -7.90 | -64.63 | -48.97 | 7.72 | -69.28 | 7.14 | -57.55 | 9.31 | -58.60 | 8.05 | 0.03 | 0.32 |
| J107-0623 | CHEMDIV | 0.82 | -7.37 | -54.28 | -63.05 | 4.48 | -55.53 | 4.45 | -56.66 | 5.33 | -58.41 | 4.75 | 0.03 | 0.34 |
| S823-0933 | CHEMDIV | 0.72 | -7.38 | -58.86 | -58.37 | 5.88 | -56.99 | 6.06 | -58.72 | 7.05 | -58.03 | 6.33 | 0.03 | 0.37 |
| AA-768/32246044 | SPECS | 0.61 | -7.29 | -73.05 | -54.91 | 8.89 | -58.04 | 10.35 | -58.74 | 9.05 | -57.23 | 9.43 | 0.03 | 0.44 |
| AO-022/43513318 | SPECS | 0.84 | -7.76 | -55.58 | -52.69 | 10.75 | -62.14 | 5.00 | -56.62 | 5.79 | -57.15 | 7.18 | 0.03 | 0.45 |
| Z1663256901 | Enamine | 0.77 | -7.31 | -46.35 | -60.07 | 4.30 | -59.93 | 7.01 | -50.02 | 7.13 | -56.67 | 6.15 | 0.03 | 0.49 |
| Z297691288 | Enamine | 0.64 | -7.20 | -58.62 | -61.19 | 6.01 | -61.33 | 9.05 | -47.22 | 10.12 | -56.58 | 8.40 | 0.03 | 0.50 |
| Z1486498158 | Enamine | 0.72 | -7.22 | -49.19 | -55.31 | 4.29 | -56.47 | 7.05 | -57.68 | 5.55 | -56.49 | 5.63 | 0.03 | 0.51 |
| Y043-8615 | CHEMDIV | 0.55 | -7.58 | -59.96 | -56.69 | 4.83 | -53.20 | 8.98 | -58.06 | 6.54 | -55.98 | 6.78 | 0.03 | 0.55 |
| AO-476/40575229 | SPECS | 0.67 | -7.08 | -54.28 | -54.32 | 5.60 | -52.31 | 6.08 | -59.80 | 9.20 | -55.48 | 6.96 | 0.03 | 0.59 |
| Z151787152 | Enamine | 0.87 | -7.25 | -55.17 | -56.73 | 9.09 | -57.80 | 10.19 | -49.87 | 7.79 | -54.80 | 9.02 | 0.03 | 0.65 |
| Z415676756 | Enamine | 0.76 | -7.14 | -54.61 | -53.12 | 7.16 | -55.18 | 5.29 | -55.74 | 6.28 | -54.68 | 6.25 | 0.03 | 0.66 |
| Z1637283691 | Enamine | 0.62 | -7.24 | -46.29 | -59.11 | 6.71 | -55.51 | 6.33 | -49.40 | 7.83 | -54.67 | 6.95 | 0.03 | 0.66 |
| Z27894777 | Enamine | 0.70 | -7.63 | -53.04 | -50.42 | 5.83 | -60.52 | 5.86 | -50.65 | 4.53 | -53.87 | 5.41 | 0.03 | 0.73 |
| C686-0424 | CHEMDIV | 0.64 | -7.39 | -65.46 | -65.51 | 6.80 | -47.51 | 9.77 | -47.71 | 5.83 | -53.58 | 7.46 | 0.03 | 0.75 |
| Z803576812 | Enamine | 0.82 | -7.30 | -52.89 | -67.15 | 8.08 | -41.34 | 7.67 | -52.15 | 4.63 | -53.55 | 6.79 | 0.03 | 0.76 |
| Z409848340 | Enamine | 0.64 | -7.12 | -56.34 | -45.86 | 7.64 | -55.62 | 5.83 | -58.92 | 7.37 | -53.47 | 6.95 | 0.03 | 0.76 |
| Z1213680306 | Enamine | 0.62 | -7.24 | -54.88 | -60.87 | 7.00 | -39.80 | 6.37 | -58.90 | 6.38 | -53.19 | 6.58 | 0.03 | 0.79 |
| Z1575716539 | Enamine | 0.62 | -7.11 | -59.31 | -53.89 | 11.13 | -57.82 | 9.87 | -47.41 | 6.77 | -53.04 | 9.26 | 0.02 | 0.80 |
| Y044-5004 | CHEMDIV | 0.79 | -7.46 | -49.10 | -61.37 | 5.64 | -39.61 | 8.12 | -53.08 | 3.43 | -51.35 | 5.73 | 0.02 | 0.95 |
| Z3215926529 | Enamine | 0.75 | -7.22 | -56.34 | -53.79 | 6.48 | -51.92 | 6.94 | -46.60 | 8.27 | -50.77 | 7.23 | 0.02 | 1.00 |
| AN-652/43162802 | SPECS | 0.67 | -7.12 | -60.61 | -46.76 | 11.40 | -52.55 | 6.57 | -52.78 | 7.03 | -50.70 | 8.33 | 0.02 | 1.00 |
| Z17356689 | Enamine | 0.70 | -7.33 | -54.04 | -51.20 | 5.60 | -45.70 | 5.19 | -54.62 | 7.32 | -50.51 | 6.03 | 0.02 | 1.02 |
| Y043-6251 | CHEMDIV | 0.84 | -7.43 | -58.81 | -52.73 | 7.99 | -43.87 | 8.79 | -52.70 | 5.41 | -49.76 | 7.39 | 0.02 | 1.08 |
| AQ-086/43467809 | SPECS | 0.83 | -6.97 | -43.86 | -51.89 | 4.24 | -45.23 | 5.91 | -52.13 | 4.88 | -49.75 | 5.01 | 0.02 | 1.08 |
| 8020-2885 | CHEMDIV | 0.57 | -8.76 | -49.35 | -39.90 | 7.53 | -47.98 | 3.65 | -60.68 | 4.51 | -49.52 | 5.23 | 0.02 | 1.10 |
| Y043-7355 | CHEMDIV | 0.81 | -7.72 | -52.35 | -58.46 | 6.23 | -44.96 | 9.95 | -37.59 | 8.08 | -47.00 | 8.08 | 0.01 | 1.32 |
| 8020-3093 | CHEMDIV | 0.69 | -7.89 | -57.82 | -46.78 | 7.83 | -45.74 | 7.41 | -46.46 | 6.15 | -46.32 | 7.13 | 0.01 | 1.38 |
| Z1870254469 | Enamine | 0.61 | -7.41 | -50.74 | -39.85 | 7.17 | -41.72 | 4.45 | -43.80 | 7.22 | -41.79 | 6.28 | 0.01 | 1.77 |
| CHEMBL3617209 | CHEMBL | 0.83 | -2.65 | -38.50 | -40.97 | 11.53 | -34.66 | 6.00 | -29.65 | 6.43 | -31.32 | 7.99 | 0.00 | 2.67 |
| 0527-0155 | CHEMDIV | 0.72 | -8.28 | -47.32 | -32.39 | 13.40 | -15.17 | 9.61 | -40.23 | 7.47 | -29.26 | 10.16 | 0.00 | 2.84 |

Table S 10: The ADME/Toxicity values of the 28 hit molecules obtained from this study in addition to the reference molecule “CHEMBL3617209” performed by MetaDrug/MetaCore.

| **Name** | **AMES (TP)** | **BBB, log ratio (TP)** | **Cytotoxicity model, -log GI50 (M) (TP)** | **MRTD (TP)** | **Prot-bind, log t (TP)** | **Prot-bind, % (TP)** | **G-LogP (TP)** | **WSol, log mg/L (TP)** | **Anemia (TP)** | **Carcinogenicity (TP)** | **Carcinogenicity Mouse Female (TP)** | **Carcinogenicity Mouse Male (TP)** | **Carcinogenicity Rat Female (TP)** | **Carcinogenicity Rat Male (TP)** | **Cardiotoxicity (TP)** | **Genotoxicity (TP)** | **Hepatotoxicity (TP)** | **Nephrotoxicity (TP)** | **Neurotoxicity (TP)** | **Liver Cholestasis (TP)** | **Liver Lipid Accumulation (TP)** | **Liver Necrosis (TP)** | **Liver Weight Gain (TP)** | **Kidney Necrosis (TP)** | **Kidney Weight Gain (TP)** | **Nephron Injury (TP)** | **SkinSens, EC3 (TP)** | **Nasal pathology (TP)** | **Testicular toxicity (TP)** | **Pulmonary toxicity (TP)** | **Epididymis toxicity (TP)** |
| --- | --- | --- | --- | --- | --- | --- | --- | --- | --- | --- | --- | --- | --- | --- | --- | --- | --- | --- | --- | --- | --- | --- | --- | --- | --- | --- | --- | --- | --- | --- | --- |
| **Z807707434** | 0.65 (58.73) | -0.66 (55.17) | 4.89 (53.80) | 0.56 (60.14) | -0.12 (55.17) | 44.55 (61.07) | 1.43 | 2.05 | 0.51 (59.59) | 0.39 (64.93) | 0.25 (64.93) | 0.49 (61.59) | 0.14 (64.93) | 0.31 (64.93) | 0.10 (50.43) | 0.34 (60.94) | 0.36 (61.07) | 0.33 (59.59) | 0.39 (59.59) | 0.44 (59.59) | 0.32 (50.43) | 0.32 (43.44) | 0.23 (50.43) | 0.38 (58.73) | 0.24 (61.07) | 0.24 (59.59) | 13.07 (36.64) | 0.08 (55.97) | 0.36 (60.00) | 0.09 (63.16) | 0.30 (60.00) |
| **D264-0862** | 0.43 (37.41) | -0.70 (33.69) | 5.37 (36.86) | 0.14 (45.64) | 0.04 (33.69) | 86.96 (39.02) | 3.19 | 0.75 | 0.15 (45.64) | 0.30 (40.06) | 0.47 (40.06) | 0.56 (40.06) | 0.26 (40.06) | 0.18 (40.06) | 0.10 (33.90) | 0.30 (40.06) | 0.27 (39.24) | 0.12 (45.64) | 0.12 (37.18) | 0.38 (39.02) | 0.24 (34.66) | 0.35 (39.02) | 0.16 (37.53) | 0.13 (34.82) | 0.12 (41.46) | 0.21 (41.46) | 23.65 (30.80) | 0.26 (37.39) | 0.45 (39.18) | 0.15 (41.10) | 0.17 (37.39) |
| **G605-0598** | 0.30 (26.11) | -0.51 (33.66) | 5.21 (30.62) | 0.38 (31.42) | -0.22 (33.66) | 90.12 (33.66) | 2.47 | 0.3 | 0.26 (32.70) | 0.16 (30.77) | 0.26 (30.58) | 0.27 (30.58) | 0.19 (30.77) | 0.13 (30.77) | 0.03 (29.34) | 0.41 (30.58) | 0.20 (36.09) | 0.09 (36.09) | 0.24 (30.18) | 0.46 (33.66) | 0.36 (31.21) | 0.60 (31.40) | 0.57 (28.99) | 0.08 (29.87) | 0.19 (30.14) | 0.18 (33.99) | 31.73 (28.46) | 0.03 (37.80) | 0.18 (37.80) | 0.10 (35.29) | 0.15 (30.18) |
| **Z74543901** | 0.48 (31.45) | -0.53 (30.85) | 4.57 (33.50) | 0.93 (30.98) | -0.50 (30.85) | 69.27 (29.79) | 2.63 | 2.52 | 0.51 (30.98) | 0.46 (36.94) | 0.50 (29.57) | 0.58 (36.94) | 0.37 (29.61) | 0.76 (36.94) | 0.42 (29.34) | 0.60 (29.68) | 0.62 (36.94) | 0.44 (31.88) | 0.65 (28.76) | 0.59 (29.79) | 0.61 (32.32) | 0.16 (30.56) | 0.66 (36.94) | 0.62 (29.65) | 0.60 (31.88) | 0.66 (31.54) | 15.01 (28.16) | 0.37 (42.41) | 0.52 (42.41) | 0.76 (36.94) | 0.40 (42.41) |
| **V008-1978** | 0.63 (33.00) | -0.45 (36.19) | 5.32 (42.59) | 0.24 (37.82) | 0.44 (36.19) | 68.89 (37.82) | 2.44 | 1.52 | 0.36 (35.83) | 0.03 (39.37) | 0.04 (44.74) | 0.09 (44.74) | 0.09 (39.37) | 0.08 (39.37) | 0.44 (35.83) | 0.09 (36.19) | 0.15 (39.37) | 0.10 (40.58) | 0.08 (38.67) | 0.03 (38.85) | 0.42 (39.37) | 0.41 (37.12) | 0.25 (40.58) | 0.21 (37.12) | 0.02 (50.17) | 0.23 (40.58) | 65.95 (24.03) | 0.22 (34.63) | 0.44 (36.19) | 0.13 (40.58) | 0.24 (36.19) |
| **F294-0607** | 0.17 (43.00) | -0.99 (48.34) | 4.70 (45.68) | 0.22 (48.34) | 0.25 (48.34) | 78.65 (48.34) | 3.24 | 1.76 | 0.69 (48.34) | 0.14 (48.34) | 0.08 (48.34) | 0.13 (47.19) | 0.18 (47.19) | 0.13 (47.19) | 0.41 (43.75) | 0.37 (48.34) | 0.17 (48.34) | 0.12 (48.34) | 0.10 (43.75) | 0.20 (46.03) | 0.39 (37.89) | 0.65 (48.34) | 0.43 (43.75) | 0.12 (41.30) | 0.06 (47.19) | 0.19 (48.34) | 28.44 (31.47) | 0.18 (38.49) | 0.31 (47.19) | 0.36 (38.49) | 0.23 (47.19) |
| **V019-9483** | 0.36 (34.55) | -0.81 (36.66) | 5.65 (40.51) | 0.52 (40.30) | 0.07 (36.66) | 81.53 (40.30) | 2.98 | 1.7 | 0.34 (36.76) | 0.05 (39.32) | 0.06 (38.98) | 0.04 (38.30) | 0.07 (38.84) | 0.05 (38.84) | 0.36 (33.40) | 0.25 (38.30) | 0.19 (40.18) | 0.14 (38.98) | 0.25 (38.26) | 0.19 (39.21) | 0.21 (36.36) | 0.48 (36.34) | 0.07 (39.63) | 0.40 (38.98) | 0.08 (38.19) | 0.19 (38.98) | 54.37 (25.45) | 0.38 (37.52) | 0.45 (38.26) | 0.05 (35.02) | 0.26 (38.26) |
| **AF-399/33695064** | 0.78 (45.19) | -0.20 (57.64) | 4.98 (47.39) | 0.03 (48.39) | 0.00 (57.64) | 75.63 (43.85) | 2.31 | 1.82 | 0.38 (44.98) | 0.22 (55.66) | 0.23 (54.09) | 0.27 (54.09) | 0.13 (54.09) | 0.06 (54.09) | 0.13 (43.16) | 0.26 (55.95) | 0.20 (52.36) | 0.24 (52.49) | 0.33 (52.36) | 0.18 (48.39) | 0.21 (52.36) | 0.55 (43.50) | 0.34 (45.99) | 0.16 (42.72) | 0.16 (50.40) | 0.24 (52.49) | 16.85 (45.33) | 0.10 (45.28) | 0.14 (55.95) | 0.06 (46.74) | 0.07 (55.95) |
| **F818-0233** | 0.37 (38.81) | 0.15 (33.81) | 4.78 (41.41) | 0.80 (31.78) | -0.30 (33.81) | 67.87 (38.22) | 2.21 | 1.55 | 0.69 (38.22) | 0.57 (43.39) | 0.44 (43.39) | 0.53 (43.39) | 0.37 (43.39) | 0.32 (43.39) | 0.12 (36.71) | 0.53 (43.39) | 0.51 (37.82) | 0.61 (37.82) | 0.59 (36.71) | 0.41 (37.40) | 0.37 (22.89) | 0.79 (37.82) | 0.92 (32.59) | 0.85 (38.14) | 0.58 (33.81) | 0.80 (37.82) | 11.06 (32.02) | 0.22 (30.17) | 0.31 (33.81) | 0.30 (27.27) | 0.37 (33.81) |
| **Z2482664935** | 0.40 (48.97) | -0.35 (47.27) | 5.01 (54.59) | 0.14 (62.77) | -0.16 (47.27) | 77.22 (62.77) | 1.49 | 2.16 | 0.25 (53.05) | 0.10 (62.77) | 0.21 (61.74) | 0.31 (62.77) | 0.07 (62.77) | 0.07 (62.77) | 0.10 (40.38) | 0.33 (58.39) | 0.14 (62.77) | 0.10 (53.16) | 0.27 (53.05) | 0.05 (53.05) | 0.26 (48.51) | 0.62 (46.35) | 0.38 (58.39) | 0.10 (49.66) | 0.07 (52.55) | 0.22 (53.16) | 27.55 (29.77) | 0.04 (50.33) | 0.25 (50.33) | 0.05 (61.74) | 0.29 (50.00) |
| **Y500-0018** | 0.34 (48.61) | -0.58 (43.85) | 5.08 (48.35) | 0.38 (56.79) | -0.06 (43.85) | 79.78 (46.00) | 1.76 | 0.57 | 0.13 (40.55) | 0.14 (59.75) | 0.21 (45.99) | 0.40 (48.61) | 0.08 (45.99) | 0.14 (45.99) | 0.17 (51.69) | 0.30 (45.69) | 0.26 (53.76) | 0.30 (53.76) | 0.38 (45.99) | 0.36 (45.05) | 0.25 (37.42) | 0.77 (51.69) | 0.72 (48.95) | 0.32 (46.86) | 0.09 (51.69) | 0.56 (53.76) | 16.25 (42.18) | 0.10 (52.82) | 0.15 (52.82) | 0.08 (50.31) | 0.36 (43.85) |
| **AG-205/36628032** | 0.52 (26.98) | -0.65 (32.23) | 4.84 (30.55) | 0.15 (32.23) | 0.07 (32.23) | 59.21 (29.81) | 2.54 | 0.69 | 0.30 (36.01) | 0.29 (34.12) | 0.40 (34.12) | 0.32 (34.12) | 0.24 (34.12) | 0.20 (34.12) | 0.71 (32.23) | 0.51 (34.12) | 0.19 (33.98) | 0.28 (36.01) | 0.15 (30.22) | 0.29 (31.80) | 0.48 (31.90) | 0.75 (30.80) | 0.58 (30.45) | 0.19 (30.69) | 0.21 (34.03) | 0.31 (34.03) | 29.43 (22.92) | 0.20 (31.93) | 0.36 (31.93) | 0.54 (33.83) | 0.36 (31.93) |
| **ZC46-0199** | 0.20 (33.09) | -0.78 (36.62) | 4.94 (37.32) | 0.30 (42.77) | 0.21 (36.62) | 88.69 (36.62) | 2.72 | 1.59 | 0.18 (57.09) | 0.07 (42.31) | 0.05 (36.26) | 0.12 (37.22) | 0.02 (37.22) | 0.01 (37.22) | 0.30 (41.56) | 0.20 (40.53) | 0.16 (41.55) | 0.16 (57.09) | 0.08 (41.18) | 0.09 (41.18) | 0.39 (37.25) | 0.45 (57.09) | 0.31 (41.55) | 0.10 (45.75) | 0.09 (42.77) | 0.24 (41.55) | 32.31 (30.04) | 0.07 (36.73) | 0.14 (44.15) | 0.07 (43.50) | 0.07 (44.15) |
| **Z605272712** | 0.84 (39.01) | -0.67 (37.46) | 4.92 (43.88) | 0.24 (44.49) | 0.12 (37.62) | 48.69 (43.10) | 1.54 | 2.13 | 0.14 (42.92) | 0.17 (52.75) | 0.31 (52.75) | 0.34 (52.75) | 0.07 (52.75) | 0.05 (52.75) | 0.12 (41.46) | 0.29 (46.12) | 0.18 (49.77) | 0.22 (48.06) | 0.18 (43.15) | 0.28 (44.49) | 0.18 (41.70) | 0.43 (42.50) | 0.27 (41.18) | 0.12 (42.94) | 0.05 (48.06) | 0.22 (46.90) | 16.52 (29.86) | 0.02 (44.76) | 0.08 (48.11) | 0.09 (47.16) | 0.07 (48.11) |
| **ZC46-0211** | 0.20 (36.36) | -0.80 (35.71) | 5.28 (37.85) | 0.26 (39.95) | 0.19 (35.71) | 76.86 (36.57) | 2.76 | 1.65 | 0.21 (52.19) | 0.07 (41.20) | 0.05 (39.42) | 0.09 (41.20) | 0.01 (41.20) | 0.01 (41.20) | 0.29 (40.05) | 0.15 (39.01) | 0.11 (42.24) | 0.13 (52.19) | 0.08 (39.42) | 0.09 (42.04) | 0.30 (40.11) | 0.54 (52.19) | 0.34 (42.24) | 0.16 (44.98) | 0.09 (39.95) | 0.25 (42.24) | 45.63 (27.95) | 0.14 (36.59) | 0.03 (42.18) | 0.07 (38.63) | 0.06 (42.18) |
| **K786-1161** | 0.35 (37.12) | -0.63 (39.39) | 5.16 (41.76) | 0.26 (39.36) | 0.39 (39.39) | 76.44 (39.39) | 3.97 | 0.48 | 0.24 (39.55) | 0.03 (41.74) | 0.07 (41.57) | 0.14 (41.74) | 0.02 (41.74) | 0.02 (41.74) | 0.29 (42.65) | 0.20 (41.57) | 0.12 (43.00) | 0.13 (42.45) | 0.16 (43.75) | 0.02 (43.75) | 0.40 (36.16) | 0.62 (42.03) | 0.21 (43.00) | 0.05 (40.88) | 0.03 (46.42) | 0.34 (43.75) | 33.33 (26.91) | 0.30 (39.45) | 0.04 (40.88) | 0.05 (40.70) | 0.18 (40.88) |
| **Y501-9249** | 0.91 (42.42) | -0.94 (34.69) | 5.32 (36.89) | 0.62 (39.51) | -0.39 (34.69) | 53.34 (39.51) | 1.14 | 2.75 | 0.49 (39.51) | 0.68 (42.42) | 0.25 (35.22) | 0.39 (42.42) | 0.33 (38.43) | 0.29 (38.43) | 0.14 (37.64) | 0.62 (35.22) | 0.39 (37.64) | 0.40 (39.10) | 0.34 (29.80) | 0.42 (32.72) | 0.37 (35.55) | 0.58 (36.61) | 0.45 (34.55) | 0.61 (34.18) | 0.38 (31.33) | 0.46 (39.10) | 10.95 (31.44) | 0.17 (38.59) | 0.40 (38.59) | 0.21 (33.19) | 0.33 (37.64) |
| **AQ-405/42300197** | 0.68 (60.26) | -0.68 (38.13) | 5.42 (43.86) | 0.31 (72.82) | 0.05 (38.13) | 69.49 (72.82) | 2.68 | 0.44 | 0.17 (36.15) | 0.03 (68.83) | 0.02 (39.32) | 0.01 (39.00) | 0.05 (41.33) | 0.02 (41.33) | 0.67 (33.11) | 0.03 (39.00) | 0.18 (77.82) | 0.34 (77.82) | 0.16 (41.33) | 0.12 (38.98) | 0.40 (35.94) | 0.28 (36.32) | 0.16 (56.54) | 0.32 (42.09) | 0.11 (69.33) | 0.22 (73.58) | 61.71 (25.91) | 0.32 (41.33) | 0.29 (68.20) | 0.05 (39.33) | 0.30 (68.20) |
| **F684-0404** | 0.23 (35.91) | -1.04 (44.57) | 4.91 (44.64) | 0.15 (44.57) | 0.11 (44.57) | 87.90 (44.57) | 4.15 | 1.89 | 0.63 (43.96) | 0.21 (46.05) | 0.14 (43.96) | 0.11 (43.96) | 0.30 (46.05) | 0.34 (46.05) | 0.39 (38.91) | 0.28 (43.96) | 0.19 (47.49) | 0.18 (46.13) | 0.07 (42.20) | 0.27 (48.13) | 0.44 (31.51) | 0.60 (42.80) | 0.49 (42.59) | 0.32 (42.80) | 0.05 (43.96) | 0.25 (40.56) | 54.58 (29.92) | 0.17 (35.93) | 0.40 (44.49) | 0.36 (36.18) | 0.13 (43.96) |
| **0527-0155** | 0.79 (37.98) | -1.23 (31.61) | 4.85 (41.50) | 0.97 (38.92) | -0.47 (31.61) | 36.57 (38.32) | 0.41 | 3.11 | 0.14 (38.32) | 0.60 (38.65) | 0.47 (38.65) | 0.54 (38.65) | 0.48 (35.94) | 0.42 (38.65) | 0.52 (38.05) | 0.55 (34.01) | 0.39 (38.65) | 0.50 (38.32) | 0.38 (38.05) | 0.53 (33.17) | 0.30 (37.25) | 0.77 (37.55) | 0.85 (37.64) | 0.88 (38.05) | 0.55 (38.05) | 0.53 (37.65) | 8.07 (31.25) | 0.22 (33.93) | 0.39 (37.64) | 0.23 (37.71) | 0.49 (32.65) |
| **AH-487/40936989** | 0.34 (39.37) | -0.27 (37.23) | 4.79 (41.30) | 0.33 (43.91) | -0.10 (37.23) | 83.98 (39.63) | 2.78 | 1.05 | 0.54 (39.63) | 0.24 (51.92) | 0.21 (43.23) | 0.31 (43.23) | 0.40 (51.92) | 0.29 (51.92) | 0.03 (43.91) | 0.36 (43.23) | 0.26 (43.91) | 0.11 (47.30) | 0.21 (46.46) | 0.23 (41.79) | 0.21 (27.24) | 0.68 (38.10) | 0.40 (43.02) | 0.14 (38.67) | 0.17 (39.38) | 0.28 (46.46) | 30.19 (28.28) | 0.04 (37.65) | 0.18 (43.91) | 0.08 (36.56) | 0.22 (43.91) |
| **AN-988/40787604** | 0.39 (32.81) | -0.56 (31.58) | 4.76 (38.59) | 0.27 (32.93) | -0.18 (31.58) | 89.83 (32.47) | 1.78 | 1.76 | 0.35 (31.92) | 0.20 (38.11) | 0.30 (35.24) | 0.24 (35.24) | 0.28 (35.24) | 0.24 (35.75) | 0.18 (31.56) | 0.25 (34.58) | 0.12 (35.21) | 0.18 (36.19) | 0.03 (34.96) | 0.16 (35.21) | 0.40 (35.21) | 0.63 (36.19) | 0.46 (36.19) | 0.15 (31.75) | 0.04 (33.69) | 0.34 (34.38) | 31.73 (28.12) | 0.16 (39.94) | 0.48 (39.94) | 0.07 (34.81) | 0.35 (34.96) |
| **Y020-2805** | 0.34 (32.35) | 0.36 (46.48) | 4.93 (42.33) | 0.31 (39.26) | -0.22 (46.48) | 65.82 (39.77) | 2.58 | 2 | 0.33 (41.90) | 0.44 (46.48) | 0.45 (46.48) | 0.65 (46.48) | 0.38 (39.26) | 0.53 (46.48) | 0.17 (41.90) | 0.35 (39.90) | 0.37 (55.32) | 0.30 (55.32) | 0.64 (36.45) | 0.43 (36.31) | 0.41 (33.92) | 0.78 (46.48) | 0.77 (35.33) | 0.42 (31.28) | 0.14 (40.45) | 0.59 (55.32) | 12.81 (30.81) | 0.10 (36.68) | 0.23 (46.48) | 0.08 (46.48) | 0.56 (39.90) |
| **K219-1359** | 0.27 (36.86) | -0.25 (40.15) | 4.79 (44.69) | 0.65 (39.44) | -0.38 (40.15) | 75.81 (40.15) | 2.25 | 2.45 | 0.28 (38.97) | 0.22 (42.56) | 0.27 (35.56) | 0.28 (42.56) | 0.10 (36.86) | 0.09 (35.56) | 0.03 (43.06) | 0.35 (35.56) | 0.27 (39.24) | 0.24 (36.10) | 0.54 (35.74) | 0.51 (36.86) | 0.21 (36.86) | 0.41 (43.06) | 0.36 (37.20) | 0.35 (36.10) | 0.40 (32.38) | 0.29 (36.10) | 13.29 (22.59) | 0.05 (30.60) | 0.12 (42.59) | 0.15 (37.42) | 0.17 (36.86) |
| **CHEMBL3617209** | 0.34 (29.81) | -0.75 (28.68) | 4.74 (35.81) | 0.78 (31.33) | -0.35 (28.68) | 52.80 (30.28) | 1.8 | 2.56 | 0.39 (28.87) | 0.60 (31.66) | 0.43 (31.66) | 0.54 (31.66) | 0.50 (31.66) | 0.30 (31.66) | 0.06 (29.11) | 0.44 (31.66) | 0.48 (31.66) | 0.32 (29.53) | 0.52 (29.23) | 0.59 (30.28) | 0.34 (30.00) | 0.38 (28.61) | 0.29 (30.00) | 0.48 (29.33) | 0.71 (29.11) | 0.47 (30.98) | 18.93 (21.56) | 0.18 (31.21) | 0.38 (31.21) | 0.21 (29.11) | 0.45 (31.21) |
| **Z1450326974** | 0.37 (26.42) | -0.19 (25.79) | 5.30 (33.70) | 0.34 (33.20) | -0.20 (25.79) | 68.89 (27.03) | 2.76 | 1.7 | 0.22 (28.53) | 0.40 (33.04) | 0.27 (30.99) | 0.36 (27.27) | 0.23 (30.99) | 0.13 (30.99) | 0.03 (26.33) | 0.46 (30.84) | 0.12 (30.99) | 0.10 (30.32) | 0.41 (27.41) | 0.40 (30.32) | 0.24 (26.64) | 0.44 (32.76) | 0.40 (30.97) | 0.11 (26.37) | 0.06 (29.96) | 0.21 (30.32) | 14.86 (19.86) | 0.03 (23.89) | 0.11 (25.50) | 0.07 (25.83) | 0.15 (25.40) |
| **Z1780715778** | 0.44 (35.66) | -0.42 (35.03) | 5.38 (40.76) | 0.29 (43.01) | -0.12 (35.03) | 68.62 (43.01) | 1.42 | 0.75 | 0.28 (43.01) | 0.23 (48.57) | 0.22 (44.78) | 0.27 (48.57) | 0.25 (48.57) | 0.15 (48.57) | 0.26 (52.68) | 0.20 (44.78) | 0.11 (44.12) | 0.09 (43.48) | 0.12 (44.12) | 0.07 (43.30) | 0.30 (43.39) | 0.55 (42.63) | 0.25 (40.53) | 0.13 (42.63) | 0.12 (43.48) | 0.22 (43.48) | 32.92 (29.43) | 0.11 (38.10) | 0.06 (44.12) | 0.07 (43.11) | 0.03 (44.12) |
| **F687-1384** | 0.25 (45.54) | -0.68 (53.21) | 5.19 (48.15) | 0.40 (60.00) | 0.24 (53.21) | 80.78 (53.21) | 3.27 | 1.59 | 0.38 (38.25) | 0.02 (58.82) | 0.03 (39.59) | 0.03 (44.83) | 0.03 (47.12) | 0.02 (44.83) | 0.27 (46.63) | 0.19 (59.64) | 0.13 (59.64) | 0.17 (53.26) | 0.05 (45.16) | 0.08 (43.52) | 0.32 (34.83) | 0.19 (46.32) | 0.07 (60.00) | 0.16 (46.63) | 0.04 (49.09) | 0.06 (46.74) | 50.79 (31.32) | 0.24 (45.34) | 0.24 (52.83) | 0.05 (45.34) | 0.05 (47.37) |
| **S751-0309** | 0.39 (36.87) | -0.05 (50.18) | 4.97 (46.22) | -0.02 (50.18) | 0.03 (50.18) | 82.10 (50.18) | 2.62 | 1.39 | 0.25 (47.86) | 0.05 (51.87) | 0.05 (48.57) | 0.09 (48.57) | 0.04 (48.57) | 0.04 (48.57) | 0.19 (48.36) | 0.26 (46.19) | 0.10 (51.19) | 0.06 (50.18) | 0.18 (50.00) | 0.05 (50.00) | 0.28 (50.00) | 0.16 (47.86) | 0.07 (42.77) | 0.05 (45.41) | 0.02 (50.00) | 0.04 (50.18) | 36.87 (47.37) | 0.18 (49.79) | 0.05 (49.79) | 0.04 (52.36) | 0.03 (48.57) |

Table S 11: MetaDrug/MetaCore parameters of ADME QSAR Models

| **Property** | **Model Description** |
| --- | --- |
| BBB, log ratio | Blood-brain barrier penetration model. The data is presented as logarithmic values representing the ratio of metabolite concentrations in the brain to those in the plasma. A cutoff point of -0.3 is utilized, where higher values signify a greater likelihood of metabolite penetration into the brain Model specifications: Sample size (N) = 107, Coefficient of determination (R^2) = 0.89, Root Mean Square Error (RMSE) = 0.26 (Clarivate Analytics, 2023). |
| G-LogP | Lipophilicity, represented as the logarithm of the compound's octanol-water distribution coefficient. Cutoffs are set between -0.4 and 5.6. Compounds with values exceeding 5.6 are considered excessively hydrophobic. Model specifications: Sample size (N) = 13474, Coefficient of determination (R^2) = 0.95, Root Mean Square Error (RMSE) = 0.21(Clarivate Analytics, 2023). |
| Prot-bind, % | Human serum protein binding expressed as a percentage. The cutoff point is set at 50%. Compounds with a binding percentage exceeding 95% are considered highly bound, while those below 50% are classified as low binding metabolites. Model specifications: Sample size (N) = 265, Coefficient of determination (R^2) = 0.909, Root Mean Square Error (RMSE) = 10.11(Clarivate Analytics, 2023). |
| Prot-bind, log t | Affinity to human serum albumin is represented by the logarithm of the retention time. The cutoff point is at 0, where positive values indicate higher protein binding, while negative values suggest lower protein binding. The acceptable level of binding varies depending on the project's requirements. The model is constructed based on the retention times of compounds analyzed via HPLC using an immobilized HSA column. Retention times are expressed as logarithmic values. Model specifications: Sample size (N) = 95, Coefficient of determination (R^2) = 0.904, Root Mean Square Error (RMSE) = 0.2 (Clarivate Analytics, 2023). |
| WSol, log mg/L | Water solubility at 25 degrees Celsius is expressed as the logarithm of milligrams per liter (mg/L). Cutoffs range from 2 to 4, with the acceptable level of solubility varying according to the project's requirements. Model specifications: Sample size (N) = 2871, Coefficient of determination (R^2) = 0.91, Root Mean Square Error (RMSE) = 0.54 (Clarivate Analytics, 2023). |

Table S 12: MetaDrug/MetaCore Parameters of Prediction of Toxic Effects

| **Property** | **Model Description** |
| --- | --- |
| AMES | The potential to exhibit mutagenicity (AMES positive) is assessed on a scale ranging from 0 to 1. A value of 1 indicates AMES positivity (mutagenicity), while a value of 0 signifies AMES negativity (non-mutagenicity). The cutoff point is set at 0.5, where values closer to zero are considered preferable. The AMES assay is conducted based on the reversal of mutations in the histidine operon within the bacterium Salmonella enterica Serovar Typhimurium. Model specifications: Sample size (N) = 1780, Coefficient of determination (R^2) = 0.69, Root Mean Square Error (RMSE) = 0.29 (Clarivate Analytics, 2023). |
| Anemia | The potential to induce anemia is assessed, with a cutoff point set at 0.5. Values exceeding 0.5 suggest the presence of potentially toxic compounds. The training set comprises chemicals and drugs known to cause anemia in vivo. Model description: Training set size (N) = 324, Test set size (N) = 51, Sensitivity = 0.82, Specificity = 0.90, Accuracy = 0.86, Matthews Correlation Coefficient (MCC) = 0.72 (Clarivate Analytics, 2023). |
| Carcinogenicity | The capacity to induce carcinogenicity in rats and mice is evaluated, with a cutoff threshold set at 0.5. Values surpassing 0.5 suggest the presence of potentially hazardous compounds. The training dataset is composed of chemicals and drugs known to elicit carcinogenic effects in vivo. Model organisms include mice and rats. Model description: Training set size (N) = 1210, Test set size (N) = 185, Sensitivity = 0.96, Specificity = 0.90, Accuracy = 0.93, Matthews Correlation Coefficient (MCC) = 0.86 (Clarivate Analytics, 2023). |
| Carcinogenicity Mouse Female | The propensity to induce carcinogenicity specifically in female mice is under evaluation, with a cutoff set at 0.5. Values exceeding 0.5 suggest the presence of potentially harmful compounds. The training dataset comprises chemicals and drugs known to provoke carcinogenic effects in vivo. The model organisms considered are solely female mice. Model description: Training set size (N) = 640, Test set size (N) = 94, Sensitivity = 0.90, Specificity = 0.93, Accuracy = 0.92, Matthews Correlation Coefficient (MCC) = 0.83 (Clarivate Analytics, 2023). |
| Carcinogenicity Mouse Male | The capacity to induce carcinogenicity specifically in male mice is being assessed, with a cutoff set at 0.5. Values surpassing 0.5 indicate the presence of potentially harmful compounds. The training dataset comprises chemicals and drugs known to elicit carcinogenic effects in vivo. The model organisms considered are solely male mice. Model description: Training set size (N) = 584, Test set size (N) = 93, Sensitivity = 0.91, Specificity = 0.88, Accuracy = 0.89, Matthews Correlation Coefficient (MCC) = 0.78 (Clarivate Analytics, 2023). |
| Carcinogenicity Rat Female | The likelihood of inducing carcinogenicity specifically in female rats is being evaluated, with a cutoff value set at 0.5. Values exceeding 0.5 suggest the presence of potentially harmful compounds. The training dataset comprises chemicals and drugs known to induce carcinogenic effects in vivo. The model organisms considered are solely female rats. Model description: Training set size (N) = 667, Test set size (N) = 120, Sensitivity = 0.90, Specificity = 0.96, Accuracy = 0.93, Matthews Correlation Coefficient (MCC) = 0.86 (Clarivate Analytics, 2023). |
| Carcinogenicity Rat Male | The potential to induce carcinogenicity specifically in male rats is being examined, with a cutoff value set at 0.5. Values surpassing 0.5 suggest the presence of potentially harmful compounds. The training dataset comprises chemicals and drugs known to induce carcinogenic effects in vivo. The model organisms considered are solely male rats. Model description: Training set size (N) = 715, Test set size (N) = 117, Sensitivity = 0.92, Specificity = 0.88, Accuracy = 0.90, Matthews Correlation Coefficient (MCC) = 0.79 (Clarivate Analytics, 2023). |
| Cardiotoxicity | The likelihood of inducing cardiotoxicity is under evaluation, with a cutoff set at 0.5. Values exceeding 0.5 indicate the potential presence of toxic compounds. The training dataset includes chemicals and drugs known to cause cardiotoxic effects in vivo across mouse, rat, and human models. Model description: Training set size (N) = 143, Test set size (N) = 30, Sensitivity = 0.80, Specificity = 1.00, Accuracy = 0.90, Matthews Correlation Coefficient (MCC) = 0.82 (Clarivate Analytics, 2023). |
| Cytotoxicity model, -log GI50 (M) | The growth inhibition of the MCF7 cell line (human Caucasian breast adenocarcinoma) is measured using the pGI50 metric. A cutoff of 6 is applied, where values falling between 6 and 8 indicate the presence of a potentially toxic metabolite. Lower values are preferred, with those less than 6 considered more desirable, and values less than 3 indicating decreased toxicity. Model description: Sample size (N) = 1474, Coefficient of determination (R^2) = 0.9, Root Mean Square Error (RMSE) = 0.05 (Clarivate Analytics, 2023). |
| Epididymis toxicity | The likelihood of inducing epididymis toxicity is under examination, with a cutoff set at 0.5. Values exceeding 0.5 suggest the potential presence of toxic compounds. The training dataset comprises chemicals and drugs known to induce epididymis toxicity in vivo across mouse, rat, and human models. Model description: Training set size (N) = 252, Test set size (N) = 42, Sensitivity = 0.90, Specificity = 0.86, Accuracy = 0.88, Matthews Correlation Coefficient (MCC) = 0.76 (Clarivate Analytics, 2023). |
| Genotoxicity | The potential to induce genotoxicity is being evaluated, with a cutoff set at 0.5. Values surpassing 0.5 suggest the presence of potentially toxic compounds. The training dataset comprises chemicals and drugs known to cause genotoxicity in vivo across mouse and rat models (Manderville, 2005). Model description: Training set size (N) = 372, Test set size (N) = 86, Sensitivity = 0.75, Specificity = 0.84, Accuracy = 0.79, Matthews Correlation Coefficient (MCC) = 0.59 (Clarivate Analytics, 2023). |
| Hepatotoxicity | The potential to induce hepatotoxicity is being assessed, with a cutoff set at 0.5. Values exceeding 0.5 suggest the presence of potentially toxic compounds. The training dataset comprises chemicals and drugs known to cause hepatotoxicity in vivo across mouse, rat, and human models. Model description: Training set size (N) = 1380, Test set size (N) = 231, Sensitivity = 0.73, Specificity = 0.88, Accuracy = 0.81, Matthews Correlation Coefficient (MCC) = 0.62 (Clarivate Analytics, 2023). |
| Kidney Necrosis | The potential to induce kidney necrosis is being evaluated, with a cutoff set at 0.5. Values exceeding 0.5 suggest the presence of potentially toxic compounds. The training dataset comprises chemicals and drugs known to cause renal necrosis in vivo across mouse, rat, and human models. Model description: Training set size (N) = 221, Test set size (N) = 42, Sensitivity = 0.96, Specificity = 1.00, Accuracy = 0.98, Matthews Correlation Coefficient (MCC) = 0.95 (Clarivate Analytics, 2023). |
| Kidney Weight Gain | The potential for inducing kidney weight gain is under examination, with a cutoff set at 0.5. Values surpassing 0.5 indicate the presence of potentially toxic compounds. The training dataset comprises chemicals and drugs known to cause kidney weight gain in vivo across mouse and rat models. Model description: Training set size (N) = 240, Test set size (N) = 49, Sensitivity = 0.95, Specificity = 1.00, Accuracy = 0.98, Matthews Correlation Coefficient (MCC) = 0.96 (Clarivate Analytics, 2023). |
| Liver Cholestasis | The potential to induce liver cholestasis is under evaluation, with a cutoff set at 0.5. Values surpassing 0.5 suggest the potential presence of toxic compounds. The training dataset comprises chemicals and drugs known to cause cholestasis in vivo across mouse, rat, and human models. Model description: Training set size (N) = 218, Test set size (N) = 35, Sensitivity = 0.79, Specificity = 0.67, Accuracy = 0.74, Matthews Correlation Coefficient (MCC) = 0.46 (Clarivate Analytics, 2023). |
| Liver Lipid Accumulation | The capacity to induce liver lipid accumulation is under evaluation, with a cutoff set at 0.5. Values exceeding 0.5 suggest the potential presence of potentially toxic compounds. The training dataset comprises chemicals and drugs known to cause lipid accumulation in vivo across mouse, rat, and human models. Model description: Training set size (N) = 172, Test set size (N) = 28, Sensitivity = 0.80, Specificity = 0.85, Accuracy = 0.82, Matthews Correlation Coefficient (MCC) = 0.64 (Clarivate Analytics, 2023). |
| Liver Necrosis | The potential to induce liver necrosis is being evaluated, with a cutoff set at 0.5. Values exceeding 0.5 suggest the presence of potentially toxic compounds. The training dataset comprises chemicals and drugs known to cause hepatic necrosis in vivo across mouse, rat, and human models. Model description: Training set size (N) = 300, Test set size (N) = 57, Sensitivity = 0.91, Specificity = 0.91, Accuracy = 0.91, Matthews Correlation Coefficient (MCC) = 0.82 (Clarivate Analytics, 2023). |
| Liver Weight Gain | The potential to induce liver weight gain is under evaluation, with a cutoff set at 0.5. Values exceeding 0.5 indicate the potential presence of compounds capable of altering liver weight. The training dataset comprises chemicals and drugs known to cause liver weight gain in vivo across mouse and rat models. Model description: Training set size (N) = 292, Test set size (N) = 52, Sensitivity = 1.00, Specificity = 1.00, Accuracy = 1.00, Matthews Correlation Coefficient (MCC) = 1.00 (Clarivate Analytics, 2023). |
| MRTD | The Maximum Recommended Therapeutic Dose (MRTD) is expressed in logarithmic milligrams per kilogram body mass per day (log mg/kg-bm/day), with a range from -5 to 3. A cutoff point of 0.5 is applied. Chemicals with higher log MRTDs may be categorized as mildly toxic compounds, while those with lower log MRTDs may be classified as highly toxic compounds. Model description: Sample size (N) = 1209, Coefficient of determination (R^2) = 0.86, Root Mean Square Error (RMSE) = 0.42 (Clarivate Analytics, 2023). |
| Nasal pathology | The likelihood of inducing nasal pathology is being assessed, with a cutoff set at 0.5. Values exceeding 0.5 indicate the potential presence of toxic compounds. The training dataset comprises chemicals and drugs known to cause nasal pathology in vivo across mouse, rat, and human models. Model description: Training set size (N) = 246, Test set size (N) = 47, Sensitivity = 1.00, Specificity = 0.93, Accuracy = 0.96, Matthews Correlation Coefficient (MCC) = 0.92 (Clarivate Analytics, 2023). |
| Nephron Injury | The possibility of inducing nephron injury is being examined, with a cutoff set at 0.5. Values surpassing 0.5 suggest the potential presence of toxic compounds. The training dataset comprises chemicals and drugs known to cause nephron injury in vivo across mouse, rat, and human models. Model description: Training set size (N) = 598, Test set size (N) = 109, Sensitivity = 0.91, Specificity = 1.00, Accuracy = 0.96, Matthews Correlation Coefficient (MCC) = 0.93 (Clarivate Analytics, 2023). |
| Nephrotoxicity | The potential to induce nephrotoxicity is under examination, with a cutoff set at 0.5. Values exceeding 0.5 suggest the potential presence of toxic compounds. The training dataset comprises chemicals and drugs known to cause nephrotoxicity in vivo across mouse, rat, and human models. Model description: Training set size (N) = 847, Test set size (N) = 154, Sensitivity = 0.90, Specificity = 0.84, Accuracy = 0.87, Matthews Correlation Coefficient (MCC) = 0.74 (Clarivate Analytics, 2023). |
| Neurotoxicity | The likelihood of inducing neurotoxicity is being assessed, with a cutoff set at 0.5. Values exceeding 0.5 suggest the potential presence of toxic compounds. The training dataset comprises chemicals and drugs known to cause neurotoxicity in vivo across mouse, rat, and human models. Model description: Training set size (N) = 175, Test set size (N) = 34, Sensitivity = 0.94, Specificity = 0.94, Accuracy = 0.94, Matthews Correlation Coefficient (MCC) = 0.88 (Clarivate Analytics, 2023). |
| Pulmonary toxicity | The likelihood of inducing pulmonary toxicity is being evaluated. The training dataset includes chemicals and drugs known to cause pulmonary toxicity in vivo across mouse, rat, and human models. A cutoff value of 0.5 is applied, with values exceeding 0.5 indicating potentially toxic compounds. Model description: Training set size (N) = 482, Test set size (N) = 87, Sensitivity = 0.89, Specificity = 0.88, Accuracy = 0.89, Matthews Correlation Coefficient (MCC) = 0.77 (Clarivate Analytics, 2023). |
| SkinSens, EC3 | The skin sensitization potential is represented by the effective concentration 3 (EC3) expressed as a percentage. Compounds with values exceeding 10 are considered weak to moderate sensitizers. Model description: Sample size (N) = 89, Coefficient of determination (R^2) = 0.67, Root Mean Square Error (RMSE) = 22.56 (Clarivate Analytics, 2023). |
| Testicular toxicity | The likelihood of inducing testicular toxicity is being evaluated, with a training dataset comprising chemicals and drugs known to cause testicular toxicity in vivo across mouse, rat, and human models. A cutoff value of 0.5 is applied, with values exceeding 0.5 indicating potentially toxic compounds. Model description: Training set size (N) = 439, Test set size (N) = 88, Sensitivity = 0.81, Specificity = 0.85, Accuracy = 0.83, Matthews Correlation Coefficient (MCC) = 0.66 (Clarivate Analytics, 2023). |

**References:**

Clarivate Analytics (2023). Website: <https://portal.genego.com> [accessed 5 July 2023]
